# *tert*-Butyl(2-oxo-2*H*-pyran-5-yl)carbamate as the First Chameleon Diene Bearing an Electron-Donating Substituent

**DOI:** 10.3390/molecules27175666

**Published:** 2022-09-02

**Authors:** Yasser M. Omar, Giulia Santucci, Kamyar Afarinkia

**Affiliations:** 1 Institute of Cancer Therapeutics, University of Bradford, Bradford BD7 1DP, UK; 2 Department Pharmaceutical Organic Chemistry, Faculty of Pharmacy, Assiut University, Assiut 71526, Egypt; 3 School of Biomedical Sciences, University of West London, London W5 5RF, UK

**Keywords:** Diels-Alder, cycloaddition, 2(*H*)-pyran-2-one, electron demand

## Abstract

The 2(*H*)-pyran-2-one bearing electron-donating *tert*-butylcarbamate (BocNH-) group at the 5- position is a “chameleon” diene and undergoes efficient Diels–Alder cycloadditions with alkene dienophiles with both electron-rich and electron-deficient substituents. Cycloadditions afford the 5-substituted bicyclic lactone cycloadducts regardless of the electronic nature of the dienophile. However, cycloadditions with electronically matched electron-deficient dienophiles proceed faster than those with electronically mismatched electron-rich dienophiles.

## 1. Introduction

**Scheme 1:**
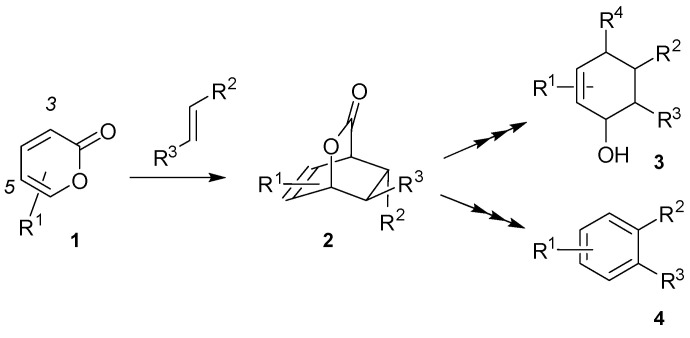
Applications of 2(*H*)-pyran-2-one in chemical synthesis.

The Diels–Alder (DA) cycloaddition of 2(*H*)pyran-2-ones [1,2,3,4,5], e.g., **1**, is a powerful and versatile methodology in synthetic organic chemistry and is widely used in the preparation of complex molecules [1,2,3,4,5,6,7,8,9,10,11,12,13,14,15,16,17,18,19,20,21,22,23,24,25,26,27,28,29,30,31,32,33,34,35,36,37,38,39]. In particular, cycloadditions of 2(*H*)pyran-2-one dienes to alkene dienophiles affords bridged bicyclic lactones, e.g., **2** which can then be transformed in few steps to highly substituted, six-membered rings, e.g., **3** (Scheme 1) [1,2,3,4,5,6,7,8,9,10,11,12,13,14,15,16,17,18,19,20,21,22,23,24,25,26,27,28,29,30,31,32,33,34,35,36,37,38,39]. Under more forcing conditions, typically at higher temperatures, cycloadditions to alkyne dienophiles and subsequent aromatization via loss of bridging CO_2_ leads to substituted benzenes, e.g., **4** [40,41,42,43,44,45,46,47].

**Scheme 2:**
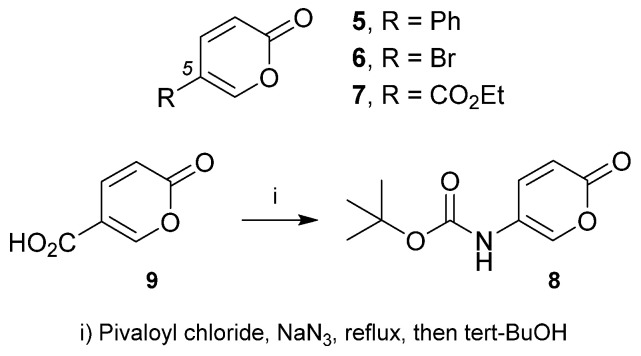
Preparation of *tert*-butyl(2-oxo-2H-pyran-5-yl)carbamate.

We have previously reported on the cycloadditions of a number of 5-substituted 2(*H*)pyran-2-ones such as 5-aryl-2(*H*)pyran-2-ones [48], **5**, 5-halo-2(*H*)pyran-2-ones [49,50,51,52,53], **6**, and 5-carboethoxy-2(*H*)pyran-2-one (ethyl coumalate), **7 [6]** (Scheme 2). Previously, we [6,7,8,9,10,11], as well as other researchers [9,10,11,12,54,55,56,57,58,59,60], have reported on the application of the cycloadditions of these dienes in synthesis. A unique and highly unprecedented feature of dienes **5** and **6** is that they are electronically unbiased. In other words, they undergo facile and efficient thermal DA cycloadditions with alkene dienophiles bearing electron-withdrawing, electron-donating, and electron-neutral substituents to mostly afford the 5-endo cycloadducts. In addition to our extensive reports on dienophiles of different electron demands that undergo cycloadditions to **5** and **6**, we have also provided a computational rationale for the observed ambident nature of the 2(*H*)pyran-2-one dienes [50,51].

It is so far unclear if the ambident nature of 5-substituted 2(*H*)pyran-2-one dienes is limited to any specific substituents. It could be argued that for **5** and **6**, the electronic bias of either the aryl or halogen substituents at the 5 position of 2(*H*)pyran-2-one is mild. So, for both **5** and **6**, the 5-substituents do not significantly influence the electronic demand of the 2(*H*)pyran-2-one diene. A more strongly electron donating or withdrawing substituent at that position may cancel the ambident nature of these dienes. To test this hypothesis, we set out to investigate the outcome of the cycloadditions of 2(*H*)pyran-2-ones with an unequivocally electron donating 5-substituent. 

Here, we report on the preparation and Diels–Alder cycloadditions of 5-(BocNH)-2(*H*)pyran-2-one, **8**, an example of a 2(*H*)pyran-2-one with an electron-donating substituent at its 5- position. We also demonstrate that this pyrone is an ambident diene that undergoes cycloaddition with electron-rich, electron-deficient, and electron-neutral alkene dienophiles. 

## 2. Results

**Scheme 3:**
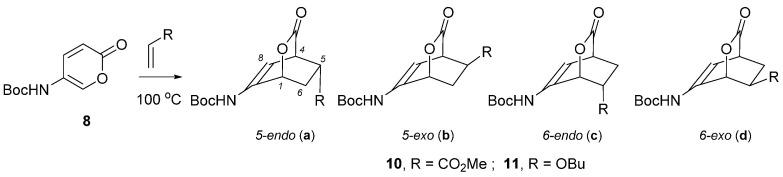
All four cycloaddition products between 8 methyl acrylate and butyl vinyl ether.

(BocNH)-2(*H*)pyran-2-one, **8,** was prepared as a white stable solid by a modification of a reported procedure through a Curtius reaction of coumalic acid, **9** (Scheme 2) [61]. We then set out to investigate the thermal Diels–Alder cycloadditions of **8** with two dienophiles, methyl acrylate as an example of an electron-deficient dienophile, and butyl vinyl ether as an example of an electron-rich dienophile to afford cycloadducts **10** and **11** (Scheme 3, R = -CO_2_Me or -OBu, all possible cycloadducts are shown for completeness). 

Cycloadditions with methyl acrylate were performed by heating a solution of **8** in excess dienophile at 100 °C and proceeded smoothly to afford cycloadducts **10**. Analysis of the crude reaction mixtures at time intervals by lcms and NMR revealed these to comprise only the unreacted starting material and cycloadduct products. Therefore, we were able to easily follow the course of the reactions. At the end of the reaction, cycloadduct products were isolated and purified by column chromatography. The yield and ratio of isolated cycloadducts are given below (Table 1). The reaction afforded only two of the four possible cycloadducts, those with 5-*endo* and 5-*exo* configuration, in a 9:1 ratio (negligible presence of other cyloadducts was also confirmed from analysis of crude NMR). The configurations of the cycloadducts, both in isolated samples and in the crude reaction mixture, can be clearly and unequivocally assigned from the analysis of their NMR spectra according to the well-precedented criteria set out earlier by Posner [1] and us [49,51] (see Supplementary Materials). The criteria are highly reliable and are based on the NMR analysis of well over 50 isolated and fully characterized cycloadducts, the configuration of a number which are unequivocally corroborated by X-ray crystallography [51].

Cycloadditions with electron-rich butyl vinyl ether were similarly performed by heating a solution of **8** at 100 °C in excess dienophile to afford cycloadducts **11** (Scheme 3, Table 1). Analysis of the crude reaction mixtures at time intervals by lcms and NMR again revealed that the reactions proceeded cleanly, affording only two cycloadducts. At the end of the reaction, the cycloadduct products were isolated and purified by column chromatography. The yield and ratio of isolated cycloadducts are given below (Table 1). The reaction affords only two of the four possible cycloadducts, with a 5-*endo* and 5-*exo* configuration, in an 8:2 ratio (the absence of other cycloadducts was also confirmed from the analysis of crude NMR).

The formation of the *endo* cycloadduct as the major product is entirely expected and consistent with a secondary orbital interaction to stabilize its transition state (see later). The formation of the 5-substituted cycloadduct in the reaction with methyl acrylate is also expected, assuming the directing effects of an electron-donating BocNH- group at the 5 position of 2(*H*)pyran-2-one diene. However, the formation of the 5-substituted cycloadduct in the reaction with butyl vinyl ether is wholly expected.

In fact, the only difference between the two reactions appeared to be their rate. Whilst cycloaddition with methyl acrylate was facile and quick, cycloaddition with butyl vinyl ether was sluggish and slow, although it afforded a good yield eventually. 

To better understand the difference between the two reactions, we decided to measure and compare the rates of cycloadditions. Since, for both cycloadditions, the dienophile is used in very large excess, reactions become de facto first order in 5-(BocNH)-2(H)pyran-2-one, **8**.

**Figure 1:**
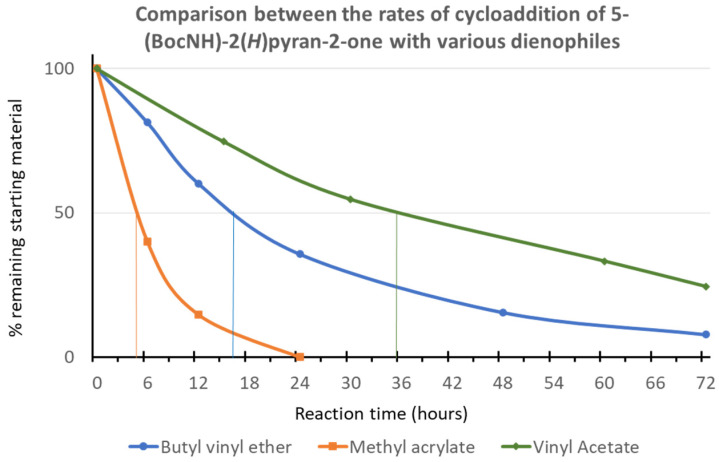
Comparison between the rates of cycloaddition of 8 with dienophiles at 100 °C.

We determined the rate of formation of cycloadduct **10**, from the reaction between **8** and methyl acrylate and the rate of formation of cycloadduct **11**, from the reaction between **8** and butyl vinyl ether (Figure 1). The results confirmed our observation that the cycloaddition of **8** with methyl acrylate (initial rate = 24 mmol/h, t_½_ = 5 h, pseudo-first-order rate constant = 38.5 × 10^−6^ s^−1^) was considerably faster than the cycloaddition to butyl vinyl ether (initial rate = 7.3 mmol/h, t_½_ = 16.5 h, pseudo-first-order rate constant = 11.7 × 10^−6^ s^−1^) when reactions are carried out with the same initial concentrations and reaction temperature. As a side note, the ratio of the cycloadducts remained roughly unchanged throughout the reactions (see Supplementary Materials), confirming that the reactions are under kinetic control.

The faster rate of cycloaddition of **8** to methyl acrylate than to butyl vinyl ether, would be consistent with 5-(BocNH)-2(*H*)pyran-2-one acting as the diene partner in a normal electron demand cycloaddition with methyl acrylate.

Finally, to investigate the scope of the cycloaddition, we carried out the reaction of 5-(BocNH)-2(*H*)pyran-2-one, **8**, with a range of other dienophiles including methyl metacrylate, acrylonitrile, and styrene. All reactions proceeded smoothly to cleanly afford the cycloadducts **12**-**16,** respectively (Table 1). The only exception was in the cycloaddition to vinyl acetate.

**Scheme 4:**
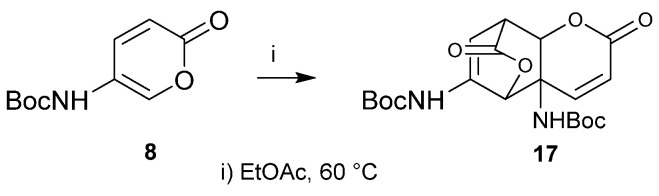
Dimerization of compound **8**.

Under similar conditions to the one used for other dienophiles, the reaction between 5-(BocNH)-2(H)pyran-2-one, **8**, and vinyl acetate proceeds very slowly and affords the expected cycloadduct **15** only as a minor product. The chromatographic isolation of the major product in this reaction afforded a white amorphous solid which was not amenable to X-ray crystal structure determination. However, based on its spectroscopic data, the byproduct is proposed to be a [4+2] cycloadduct **17**, a dimer of **8** (Scheme 4). The ready formation of a dimer during the cycloadditions of pyrones is precedented. Unsubstituted 2(*H*)pyran-2-one is reported to undergo dimerization under photochemical and high-pressure conditions [62,63,64]. To the best of our knowledge, however, this is the first example of the dimerization of pyrones under thermal conditions. Spectroscopic data support the structure of the dimer to be **17** (see Supplementary Materials), mainly due to the presence of two coupled olefinic protons at 6.02 and 6.47 ppm, indicative of a CH=CH-C=O system. 

Before we could analyze the cycloaddition of **8** with vinyl acetate, we had to find a means of limiting the dimerization and formation of **10** and maximizing the formation of the cycloadducts.

We first established that the formation of **17** is irreversible. When a sample of **17** was heated at 100 °C in a large excess of methyl acrylate as a dienophiles trap, we detected no cycloadduct **10**, or any **8,** in the crude reaction mixture by NMR and lcms. We concluded that **17** does not break down to **8**, so its formation must be irreversible under these reaction conditions. 

We then compared the rates of the formation of cycloadduct **15**, a product of the reaction of **8** with vinyl acetate and dimer **17**. We prepared two solutions of equal concentration (50 mg/mL or 0.24 M). One contained **8** in vinyl acetate and the other contained **8** in ethyl acetate. Ethyl acetate was chosen as its physiochemical properties (bp, polarity, etc.) are roughly similar to those of vinyl acetate. The solutions were transferred to two sealed tubes and heated at 100 °C under identical conditions. We used NMR and lcms on small aliquots withdrawn from each reaction to quantify the formation of products over a period of time and used that data to compare the rates of reactions and found them to be similar. 

Since the cycloaddition was carried out in large excess of vinyl acetate, the reaction becomes a de facto unimolecular reaction and the rate of cycloaddition is directly proportionate to the concentration of 5-(BocNH)-2(*H*)pyran-2-one, **8**. In contrast, the dimerization of **8** is bimolecular. To confirm this, we doubled the concentration of **8** in the above rate experiments (100 mg/mL or 0.48 M). We observed that whilst the initial rate of cycloaddition doubled, in line with a de facto unimolecular reaction, the initial rate for dimerization quadrupled, confirming the reaction is bimolecular. 

Based on these observations, we concluded that carrying out the reactions under more dilute conditions would reduce the formation of dimer **17**, whilst proportionally increasing the amount of cycloadduct, **15**. Hence, by carrying out cycloadditions of **8** and vinyl acrylate in concentrations around 0.10 M, we were thus able to effectively reduce the yield of **17** at the end of the reaction to negligible. 

As was the case in the cycloadditions of methyl acrylate, cycloadduct products were isolated at the end of the reaction and purified by column chromatography. A similar analysis of the crude reaction mixtures and purified cycloadducts by NMR allowed us to determine the regio- and stereoselectivity of the cycloadditions. Surprisingly, the cycloaddition between **8** and vinyl acetate afforded three cycloadducts which have the 5-*endo*, 5-*exo,* and 6-*endo* configuration in a 25 : 63 : 12 ratio. In other words, whilst the cycloaddition is still regioselective and favors the 5-substituted cycloadduct, the stereoselectivity is reversed and instead of the *endo* isomer, the *exo* isomer is favored. We have previously shown that *exo* isomers are more thermodynamically stable than *endo* isomers, although the latter are favored on kinetic grounds [1]. However, the shift from *endo* to *exo* isomer being major in this cycloaddition is not due to thermodynamic factors, since the ratio of the isomers does not change upon prolonged heating of the reaction mixture. This suggests that the preference for the *exo* isomer is not a result of the cycloaddition being reversible. However, the exact reason why the *exo* isomer is the favored cycloadduct in the cycloadditions of vinyl acetate remains unclear. We should, however, note that in the cycloadditions of 5-Br-2(*H*)pyran-2-one, **6**, we observed poor stereoselectivity for some dienophiles which could not be rationalized even with computational models [50,51].

We also measured the rate of cycloaddition as described above. This confirmed that the cycloaddition with vinyl acetate is slower (initial rate = 3.4 mmol/h, t_½_ = 36 h, pseudo-first-order rate constant = 5.35 × 10^−^^6^ s^−^^1^) than both the cycloadditions to methyl acrylate and butyl vinyl ether (Figure 1). The slow rate of reaction between **8** and vinyl acetate can explain why the dimerization of **8** becomes a major byproduct in that cycloaddition. Presumably, the slow rate of cycloaddition allows the normally slow dimerization to compete with the cycloaddition, whereas in the other cycloadditions, the rates for dimerization are negligible.

Finally, to confirm that the cycloaddition to vinyl acetate proceeds more slowly than that to butyl vinyl ether, we carried out a competition experiment. 5-(BocNH)-2(*H*)pyran-2-one was heated with a large excess of a 1:1 v/v mixture of vinyl acetate and butyl vinyl ether. The reaction mixture was analyzed after 24, 48, and 72 h by NMR for the relative ratio of the cycloadducts from the two dienophiles. At all-time points, cycloadducts to butyl vinyl ether were predominant, confirming that the cycloaddition to butyl vinyl ether is faster than that to vinyl acetate.

## 3. Discussions and Conclusions

**Figure 2:**
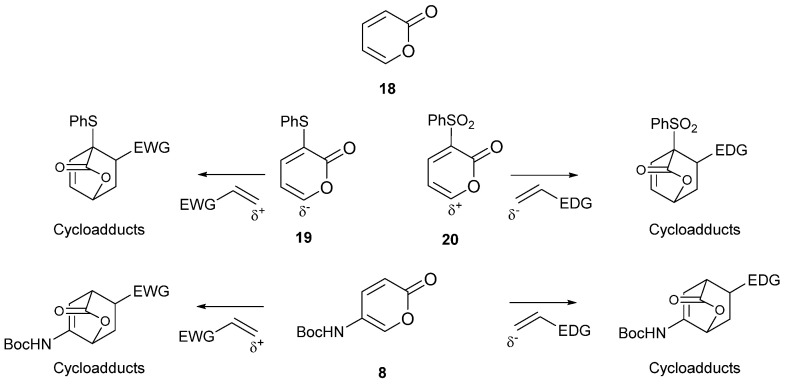
Electron demand in the cycloadditions of 3-phenylsulfenyl-2(*H*)pyran-2-one and 3-phenylsulfonyl-2(*H*)pyran-2-one.

Ring substituents play a critical role in the cycloadditions of 2(*H*)pyran-2-ones. Generally speaking, the unsubstituted ring 2(*H*)pyran-2-one, **18** (Figure 2), undergoes cycloadditions only under harsh conditions and with alkynes to afford benzenes following the tandem loss of CO_2_ [1,2,3,4,5,40,41,42,43,44,45,46,47]. Examples of cycloadditions of 2(*H*)pyran-2-one, **18,** with alkenes to afford bridged bicyclic lactones do exist, but are rare [65,66,67,68,69,70]. The introduction of substituents to the ring does allow the cycloaddition to proceed under relatively mild thermal conditions. Prior to our work, the function of substituents was considered to be that of altering the electronic demand of the 2(*H*)pyran-2-one and matching it with that of the dienophile. This matching results in lowering the activation energy for the reactions by reducing the HOMO-LUMO energy gaps, as we have shown before [50,51]. This will, in turn, result in increasing the rate of cycloadditions between electronically matched 2(*H*)pyran-2-ones and dienophiles. Furthermore, the presence of substituents on 2(*H*)pyran-2-one is also associated with the regioselectivity of the cycloadditions by matching electron density at the reaction centers (Figure 2). This is best exemplified by the work of Posner who demonstrated that 3-phenylsulfenyl-2(*H*)pyran-2-one, **19**, reacts with electron-deficient dienes [71] whereas 3-phenylsulfonyl-2(*H*)pyran-2-one, **20**, reacts with electron-rich dienes (Figure 2) [40,41,72]. In both of these examples, substituents favor the formation of the 5-substituted cycloadducts which is consistent with the electronic features of a normal electron demand for **19** and an inverse electron demand for **20** (Figure 2).

The results outlined here suggest that the cycloaddition of 5-substituted 2(*H*)pyran-2-ones does not follow a similar pattern. It is expected that the presence of the electron-donating BocNH- at the 5- position lowers the HOMO-LUMO energy gap with methyl acrylate, which in turn reduces the activation energy for the cycloaddition, resulting in a fast reaction. Indeed, the rate of the reaction of **8** with its electronically matched dienophile, methyl acrylate, is quite fast (t_½_ = 5 h), compared with the rate of reaction with its electronically mismatched dienophile, butyl vinyl ether (t_½_ = 16.5 h). However, the observation that the cycloaddition to vinyl acetate is slower (t_½_ = 36 h) than that to both methyl acrylate and butyl vinyl ether, strongly hints that the reactivity of this diene does not directly correlate to the electron density of the dienophile. 

The other unexpected feature of these cycloadditions is that they always afford the 5-substituted cycloadduct as the major regioisomer, regardless of the substituent on the dienophile. The observation that the electron-donating BocNH- favors the formation of the 5-substituted cycloadducts with methyl acrylate is consistent with the electronic features of **8**. However, the observation that it also favors the formation of the 5-substituted cycloadducts with butyl vinyl ether is wholly inconsistent with the expected electronic features of **8**. 

Considering the fact that 2(*H*)pyran-2-ones with less electronically discerning 5-substituents such as aryl and halogens are also chameleon dienes [48,49,50,51,52,53], our observations point to the possibility that 2(*H*)pyran-2-ones with any substituent at its 5- position may be capable of exhibiting chameleon properties. To demonstrate this, we plan to carry out a detailed study of the role of electron-withdrawing substituents at the 5- position of 2(*H*)pyran-2-ones, and whether these are also chameleon dienes, and will report our findings in due course. Other groups have sporadically reported (and our preliminary observations also confirm) that coumalates such as **7** undergo thermal cycloadditions with dienophiles bearing electron-donating [73,74,75], electron-neutral [76,77], and electron-withdrawing [78,79,80] dienophiles. However, a systematic study of their aptitude towards both electron-rich and electron-deficient dienes has not yet been carried out.

Clearly, understanding the highly unprecedented chameleon-like properties of 5-substituted 2(*H*)pyran-2-ones would be quite useful in understanding the role of electron demand in Diels–Alder cycloadditions. More importantly, however, identification of the range of substituted 2(*H*)pyran-2-one dienes that lack electron demand would be useful in extending the applicability of the pyrone cycloaddition methodology in target synthesis, particularly in diversity-oriented synthesis.

## Figures and Tables

**Table 1:** Results from cyloadditions of 5-(BocNH)-2(H)pyran-2-one, **8,** with various dienophiles.

Dienophile	Yield * (%)	Cycloadduct	5-endo:5-exo:6-endo:6-exo
Methyl acrylate	79	10	91:9:0:0
Methyl metacrylate	72	12	93:7:0:0
Acrylonitrile	76	13	50:50:0:0
Styrene	67	14	70:30:0:0
Vinyl acetate	65	15	25:63:12:0
Vinylene carbonate	51	16	67:33: 0:0
Butyl vinyl ether	54	11	80:20:0:0

* Combined isolated yield of all stereoisomers following chromatography.

## Data Availability

Data is contained within the article or Supplementary Material.

## Supplementary Materials

The following Supplementary Materials can be downloaded at: https://www.mdpi.com/article/10.3390/molecules27175666/s1, Experimental procedures and copies of spectroscopic characterisation of all new compounds.
