# Supplementary material for: tert-Butyl(2-oxo-2H-pyran-5-yl)carbamate as the First Chameleon Diene Bearing an Electron-Donating Substituent
Source: Molecules. 2022 Sep 2;27(17):5666. doi: 10.3390/molecules27175666 (PMC9458009; doi:10.3390/molecules27175666)
Supplement: Supplementary file 1 [file molecules-27-05666-s001.zip › molecules-1846458-supplementary.pdf]

Supplementary Materials

tert-butyl(2-oxo-2H-pyran-5-yl)carbamate as the first electronically unbiased diene bearing an electron donating substituent

*Yasser M. Omar[a,b], Giulia Santucci[a], and Kamyar Afarinkia[a]**

k.afarinkia@bradford.ac.uk

[a] Institute of Cancer Therapeutics, University of Bradford, Bradford BD7 1DP, United Kingdom.

[b] Department Pharmaceutical Organic Chemistry, Faculty of Pharmacy, Assiut University, Assiut 71526, Egypt.

[c] School of Biomedical Sciences, University of West London, London W5 5RF, United Kingdom.

Table of content

Experimental procedures for cycloadditions and characterisation of compounds **8** and **10**-**17**	2
Experimental procedures for rate determination	7
Guide to determining endo and exo isomers	8
Copies of the Spectra for compounds **8**, all isomers of **10**-**16**, and compound **17**	9
Proposed assignment of ^{1}H and ^{13}C NMR signals for compound **17**	43

Experimental procedures for cycloadditions

General: Sealed tubes were purchased from sigma (cat no Z564583). Merck 9385 silica gel 60 (40-63 µm) was used for column chromatography. Melting points (Mp) were recorded on an Eisco apparatus and are uncorrected. Analtech TLC Uniplates™ # Z513059 (2000 micron) were used for purification by preparative tlc. Proton and carbon nuclear magnetic resonance (^{1}H and ^{13}C NMR) spectra were recorded in deuterochloroform (except for compound **16** which was recorded in acetone-d_6 and compound **17** which was recorded in DMSO-d_6) using a Bruker AMX400 spectrometer operating at 400 MHz and 101 MHz, respectively. Infrared (IR) spectra were recorded on a Perkin-Elmer FTIR spectrometer using KBr discs. Routine and high resolution mass spectra were run on a Micro mass Quattro Ultima and using a Thermo Orbitrap LTQ XL spectrometer in the electron impact (ESI or EI) mode.

5-(BocNH)-2(H)pyran-2-one, **8**: Pivaloyl chloride (5.73 mL, 47.5 mmol) was added dropwise over 15 minutes to a stirred solution of coumalic acid (5.02 g, 35.8 mmol) and triethylamine (5.50 mL, 39.4 mmol) in anhydrous acetone (35 mL) maintained at 0° C under a dry nitrogen atmosphere. After 30 minutes, a solution of sodium azide (3.47 g, 53.4 mmol) in a minimum amount of distilled water (less than 10mL) was added dropwise whilst maintaining the reaction at 0° C. After 1 hour, the dark red reaction mixture was poured into ice-cold water (50 mL) and was extracted with toluene (8 x 60 mL). The combined organic extracts were dried over $MgSO_4$ and then filtered. This solution was added dropwise via a pressure equalising funnel to a refluxing and stirred solution of toluene (100 mL) and tert-butanol (20 mL) over 1.5 hours. After the addition was complete, the reaction mixture was refluxed for a further 1 hour. All volatile materials were removed under reduced pressure. The resulting residue was purified by column chromatography, using 30% v/v ethyl acetate in hexane as eluent to afford the title compound as a white solid (1.0 g, 13%). Mp 128-130 °C; R_f = 0.25 (30% EtOAc in Hexane); ^{1}H NMR δ 7.77 (s, 1H, H_6), 7.28 (dd, *J* = 9.9, 2.8 Hz, 1H, H_4), 6.26 (dd, *J* = 9.9, 1.1 Hz, 1H, H_3), 6.15 (s, 1H, NH), 1.42 (s, 9H, $C(CH_3)_3$) ; ^{13}C NMR δ 160.9 (O=C_2-O), 153.0 (O=C-N), 142.4 (C_6), 141.4 (C_4), 120.6 (C_5), 116.3 (C_3), 81.6 ($\underline{C}(CH_3)_3$), 28.2 ($C(\underline{C}H_3)_3$); IR 3277 (N-H), 2978 (C-H), 1699 (C=O pyrone), 1625 (C=O Boc). MS m/z 234 $(M+Na)^+$, 212 $(MH)^+$, 156 $[MH -CH_2{=}CMe_2]^+$, 112 [MH -CO_2, -$CH_2{=}CMe_2]^+$; HRMS for $[MH]^+$ calculated 212.0923, found 212.0947.

Cycloaddition with methyl acrylate: A stirred solution of 5-NHBoc-2-pyrone (100 mg, 0.47 mmol) and a few crystals of butylated hydroxytoluene, BHT (antipolymerisation reagent), in methyl acrylate (4 mL) in a sealed tube was immersed in an oil bath maintained at 100 °C for 24 hours. After cooling, all volatiles were removed under reduced pressure, and the residue was subjected to silica gel column chromatography, using 50% v/v diethyl ether in hexane as eluent to afford pure 5-*endo* cycloadduct (105 mg, 75%), as well as mixed fractions of the 5-*endo* and 5-*exo* cycloadducts. The mixed fraction was purified using preparative TLC to give pure 5-*exo* isomer (5 mg, 4 %).

5-*Endo* cycloadduct **10a**: Mp 122-124 °C; R_f = 0.15 (50% diethyl ether in hexane); ^{1}H NMR δ 6.22 (s, 1H, NH), 5.84 (d, *J* = 4.3 Hz, 1H, H_8), 5.21 (s, 1H, H_1), 3.77 (dd, *J* = 6.6, 2.8 Hz, 1H, H_4), 3.65 (s, 3H, OCH_3), 2.98 (ddd, *J* = 9.7, 4.1, 2.7 Hz, 1H, H_{5exo}), 2.38 (ddd, *J* = 13.7, 9.7, 3.9 Hz, 1H, H_{6exo}), 2.25 (ddd, *J* = 13.9, 4.0, 1.6 Hz, 1H, H_{6endo}), 1.40 (s, 9H, $C(CH_3)_3$); ^{13}C NMR δ 172.6 (C_3), 171.9 (O=C-O), 151.9 (O=C-N), 139.9 (C_7), 103.5 (C_8), 81.6 ($\underline{C}(CH_3)_3$), 75.4 (C_1), 52.6 (OCH_3), 42.0 (C_4), 37.4 (C_5), 29.3 (C_6), 28.2 [$C(\underline{C}H_3)_3$]; IR 3331 (N-H), 2980 (C-H), 1770 (C=O ester), 1744 (C=O

ester), 1655 (C=O Boc). MS m/z 320 $(M+Na)^+$, 298 $(MH)^+$, 242 $[MH -CH_2=CMe_2]^+$, 198 [MH -CO_2, -$CH_2=CMe_2]^+$; HRMS for $[MH]^+$ calculated 298.1291, found 298.1292.

5-*Exo* cycloadduct **10b**: Viscous liquid; R_f = 0.09 (50% diethyl ether in hexane); ^1H NMR δ 6.39 (s, 1H, NH), 6.12 (d, *J* = 6.9 Hz, 1H, H_8), 5.13 (ddd, *J* = 4.0, 2.7, 1.7 Hz, 1H, H_1), 3.73 (s, 3H, OCH_3), 3.65 (dd, *J* = 7.0, 2.4 Hz, 1H, H_4), 2.84 (ddd, *J* = 10.8, 5.4, 2.4 Hz, 1H, H_{5endo}), 2.51 (ddd, *J* = 13.9, 5.4, 3.8 Hz, 1H, H_{6exo}), 2.12 (ddd, *J* = 13.9, 10.8, 1.7 Hz, 1H, H_{6endo}), 1.47 (s, 9H, $C(CH_3)_3$); ^{13}C NMR δ 172.7 (C^3), 171.6 (O=C-O), 152.1 (O=C-N), 139.9 (C_7), 105.3 (C_8), 81.8 ($\underline{C}(CH_3)_3$), 75.3 (C_1), 52.7 (OCH_3), 42.3 (C_4), 40.1 (C_5), 29.4 (C_6), 28.2 ($C(\underline{C}H_3)_3$); IR 3325 (N-H), 2978 (C-H), 1770 (C=O ester), 1740 (C=O ester), 1657 (C=O Boc). m/z 320 $(M+Na)^+$, 298 $[MH]^+$, 242 $[MH -CH_2=CMe_2]^+$, 198 [MH -CO_2, - $CH_2=CMe_2]^+$; HRMS for $[MH]^+$ calculated 298.1291, found 298.1300.

<u>*Cycloaddition with butyl vinyl ether:*</u> A stirred solution of 5-NHBoc-2-pyrone (100 mg, 0.47 mmol) in methyl acrylate (4 mL) in a sealed tube was immersed in an oil bath maintained at 100 °C for 3 days. All volatiles were removed under reduced pressure. The residue was dissolved in a minimum quantity of hot diethyl ether, and a few drops of hexane were added until the solution turned turbid and left for recrystallisation. The solid was filtered to afford pure 5-*exo* cycloadduct as a white solid (8 mg, 5%). The supernatant was subjected to silica gel chromatography, using 50% v/v diethyl ether in hexane as eluent to afford a mixture of 5-*endo* and 5-*exo* cycloadducts (5:1) (72 mg, 49%).

5-*Endo* cycloadduct **11a**: Viscous liquid (mixture); R_f = 0.28 (50% diethyl ether in hexane); ^1H NMR δ 6.13 (s, 1H, NH), 5.75 (d, *J* = 5.2 Hz, 1H, H_8), 5.20 (s, 1H, H_1), 3.89 (ddd, *J* = 8.1, 3.3, 2.4 Hz, 1H, H_{5exo}), 3.73 (dd, *J* = 6.4, 2.9 Hz, 1H, H_4), 3.40 (dt, *J* = 9.2, 6.6 Hz, 1H, $OC\underline{H}H$), 3.30 (dt, *J* = 9.2, 6.6 Hz, 2H, $OCH\underline{H}$), 2.50 (ddd, *J* = 14.1, 7.8, 3.9 Hz, 1H, H_{6exo}), 1.65 (ddd, *J* = 14.2, 2.4, 1.65 Hz, 1H, H_{6endo}), 1.47 – 1.42 (m, 2H, $H_{2'}$), 1.41 (s, 9H, $C(CH_3)_3$), 1.26 (dq, *J* = 14.4, 7.3 Hz, 2H, $H_{3'}$), 0.83 (t, *J* = 7.4 Hz, 3H, $H_{4'}$); ^{13}C NMR δ 172.7 (C_3), 152.1 (O=C-N), 138.7 (C_7), 102.7 (C_8), 81.6 ($\underline{C}Me_3$), 75.3 (C_1), 71.3 (C_5), 69.0 ($C_{1'}$), 44.9 (C_4), 35.1 (C_6), 31.7 ($C_{2'}$), 28.2 ($C(\underline{C}H_3)_3$), 19.3 ($C_{3'}$), 13.8 ($C_{4'}$); IR (KBr disk) 3321 (N-H), 3065 (C=C-H) 2933 (C-H), 1734 (C=O ester), 1658 (C=O Boc); MS m/z 334 [M+Na], 312 $[MH]^+$, 256 $[MH - CH_2=CMe_2]^+$, 212 [MH-CO_2, - $CH_2=CMe_2]^+$; HRMS for $[MH]^+$ calculated 312.18110, found 312.1797.

5-*Exo* cycloadduct **11b**: Mp 142-144 °C; R_f = 0.28 (50% diethyl ether in hexane); ^1H NMR δ 6.28 (s, 1H, NH), 5.86 (d, *J* = 4.5 Hz, 1H, H_8), 4.96 – 4.90 (m, 1H, H_1), 3.76 (dt, *J* = 8.9, 3.1 Hz, 1H, H_{5endo}), 3.64 (dd, *J* = 7.1, 3.2 Hz, 1H, H_4), 3.52 (dt, *J* = 8.9, 6.6 Hz, 1H, $OC\underline{H}H$), 3.29 (dt, *J* = 9.0, 6.5 Hz, 1H, $OCH\underline{H}$), 2.19 (ddd, *J* = 13.9, 8.8, 1.7 Hz, 1H, H_{6endo}), 1.90 (dt, *J* = 14.0, 3.6 Hz, 1H, H_{6exo}), 1.51 – 1.42 (m, 2H, $CH_{2'}$), 1.40 (s, 9H, $C(CH_3)_3$), 1.28 (dq, *J* = 14.4, 7.3 Hz, 2H, $H_{3'}$), 0.83 (t, *J* = 7.3 Hz, 3H, $H_{4'}$); ^{13}C NMR δ 171.9 (C_3), 152.1 (O=C-N), 140.3 (C_7), 102.4 (C_8), 81.6 ($\underline{C}Me_3$), 75.8 (C_1), 74.0 (C_5), 68.9 ($C_{1'}$), 44.7 (C_4), 34.0 (C_6), 31.8 ($C_{2'}$), 28.2 ($C(\underline{C}H_3)_3$), 19.3 ($C_{3'}$), 13.9 ($C_{4'}$); IR (KBr disk) 3260 (N-H), 3067 (C=C-H) 2961 (C-H), 1734 (C=O ester), 1657 (C=O Boc); MS m/z 334 [M+Na], 312 $[MH]^+$, 256 $[MH - CH_2=CMe_2]^+$, 212 [MH-CO_2, - $CH_2=CMe_2]^+$; HRMS for $[MH]^+$ calculated 312.18110, found 312.18506.

<u>*Cycloaddition with methyl methacrylate*</u>: A stirred solution of 5-NHBoc-2-pyrone (100 mg, 0.47 mmol) in methyl methacrylate (4 mL) in a sealed tube was immersed in an oil bath maintained at 100 °C for 24 hours. All volatiles were removed under reduced pressure, and the residue was subjected to silica gel column chromatography, using 50% v/v diethyl ether in hexane as eluent to afford pure 5-*endo* cycloadduct (100 mg, 68%) and mixed fractions, which are further purified using preparative TLC to give a pure 5-*exo* cycloadduct (6 mg, 4%).

5-*Endo* cycloadduct **12a**: Mp 109-110 °C; R_f = 0.23 (50% diethyl ether in hexane); ^1H NMR δ 6.13 (s, 1H, NH), 5.88 (d, *J* = 4.9 Hz, 1H, H_8), 5.10 (s, 1H, H_1), 3.64 (s, 3H, OCH_3), 3.56 (d, *J* = 6.6 Hz, 1H, H_4), 2.66 (dd, *J* = 13.9, 1.5 Hz, 1H, H_{6endo}), 1.91 (dd, *J* = 14.2, 3.7 Hz, 1H, H_{6exo}), 1.40 (s, 9H, $C(CH_3)_3$), 1.36 (s, 3H, C_5-CH_3); ^{13}C NMR δ 174.9 (C_3), 172.4 (O=C-O), 151.8 (O=C-N), 139.6 (C_7), 105.0 (C_8), 81.6 [$\underline{C}(CH_3)_3$], 75.8 (C_1), 52.8 (OCH_3), 48.3 (C_4), 44.8 (C_5), 37.3 (C_6), 28.2 [$C(\underline{C}H_3)_3$], 26.3 (C_5-$\underline{C}H_3$); IR 3329 (N-H), 2978 (C-H), 1766 (C=O ester), 1744 (C=O ester), 1657 (C=O Boc); MS m/z 334 [M+Na], 312 $[MH]^+$, 256 $[MH - CH_2{=}CMe_2]^+$, 212 $[MH - CO_2, - CH_2{=}CMe_2]^+$; HRMS for $[MH]^+$ calculated 312.1447, found 312.1451.

5-*Exo* cycloadduct **12b**: Viscous liquid; R_f = 0.13 (50% diethyl ether in hexane); ^1H NMR δ 6.20 (s, 1H, NH), 5.98 (d, *J* = 6.8 Hz, 1H, H_8), 4.99 (ddd, *J* = 4.1, 2.7, 1.5 Hz, 1H, H_1), 3.66 (s, 3H, OCH_3), 3.44 (d, *J* = 7.0 Hz, 1H, H_4), 2.89 (dd, *J* = 14.0, 4.1 Hz, 1H, H_{6exo}), 1.51 (dd, *J* = 14.0, 1.5 Hz, 1H, H_{6endo}), 1.41 (s, 9H, $C(CH_3)_3$), 1.25 (s, 3H, CH_3); ^{13}C NMR δ 175.4 (C_3), 172.2 (O=C-O), 152.0 (O=C-N), 139.0 (C_7), 103.8 (C_8), 80.4 [$\underline{C}(CH_3)_3$], 75.7 (C_1), 52.9 (OCH_3), 48.1 (C_4), 44.8 (C_5), 36.9 (C_6), 28.2 [$C(\underline{C}H_3)_3$], 24.4 (C_5-$\underline{C}H_3$); IR 3335 (N-H), 2978 (C-H), 1769 (C=O ester), 1740 (C=O ester), 1657 (C=O Boc); MS m/z 334 [M+Na], 312 $[MH]^+$, 256 $[MH - CH_2{=}CMe_2]^+$, 212 $[MH - CO_2, - CH_2{=}CMe_2]^+$; HRMS for $[MH]^+$ calculated 312.1447, found 312.1451.

<u>*Cycloaddition with acrylonitrile*</u>: A stirred solution of 5-NHBoc-2-pyrone (100 mg, 0.47 mmol) in acrylonitrile (4 mL) in a sealed tube was immersed in an oil bath maintained at 100 °C for 24 hours. All volatiles were removed under reduced pressure, and the residue was subjected to recrystallisation using diethyl ether/ hexane with two drops of ethyl acetate to afford the 5-*exo* product as a crystalline white solid (50 mg, 40%). The supernatant was evaporated, and the residue was subjected to silica gel column chromatography, using 50% v/v diethyl ether in hexane as eluent to afford pure 5-*endo* cycloadduct (45 mg, 36%), as well as mixed fractions.

5-*Endo* cycloadduct **13a**: Mp 158-160 °C; R_f = 0.08 (50% diethyl ether in hexane); ^1H NMR δ 6.33 (s, 1H, NH), 5.96 (dd, *J* = 6.7, 2.6 Hz, 1H, H_8), 5.4 3 (s, 1H, H_1), 3.67 (dd, *J* = 6.7, 2.7 Hz, 1H, H_4), 3.12 (ddd, *J* = 9.7, 3.7, 2.6 Hz, 1H, H_{5exo}), 2.59 (ddd, *J* = 13.7, 9.7, 3.8 Hz, 1H, H_{6exo}), 2.11 (ddd, *J* = 14.0, 3.7, 1.6 Hz, 1H, H_{6endo}), 1.43 (s, 9H, $C(CH_3)_3$); ^{13}C NMR δ 170.2 (C_3), 151.9 (O=C-N), 141.1 (C_7), 119.3 (CN), 103.1 (C_8), 82.2 ($\underline{C}(CH_3)_3$), 74.2 (C_1), 41.7 (C_4), 31.3 (C_6), 28.2 ($C(\underline{C}H_3)_3$), 23.8 (C_5); IR 3331 (N-H), 2980 (C-H), 2247 (CN), 1767 (C=O ester), 1655 (C=O Boc); MS m/z 287 [M+Na], 265 $[MH]^+$, 209 $[MH - CH_2{=}CMe_2]^+$, 165 $[MH - CO_2, - CH_2{=}CMe_2]^+$; HRMS for $[MH]^+$ calculated 265.1188, found 265.1189.

5-*Exo* cycloadduct **13b**: Mp 202-204 °C; R_f = 0.07 (50% diethyl ether in hexane); ^1H NMR δ 6.27 (s, 1H, NH), 6.02 (d, *J* = 5.4 Hz, 1H, H_8), 5.17 – 5.13 (m, 1H, H_1), 3.64 (dd, *J* = 7.0, 2.4 Hz, 1H, H_4), 2.84 (ddd, *J* = 10.9, 5.3, 2.4 Hz, 1H, H_{5endo}), 2.43 (ddd, *J* = 14.0, 5.4, 3.8 Hz, 1H, H_{6exo}), 2.29 (ddd, *J* = 13.9, 10.8, 1.7 Hz, 1H, H_{6endo}), 1.41 (s, 9H, $C(CH_3)_3$); ^{13}C NMR δ 169.2 (C_3), 151.9 (O=C-N), 140.1 (C_7), 119.0 (CN), 104.1 (C_8), 82.3 ($\underline{C}Me_3$), 74.5 (C_1), 42.3 (C_4), 31.1 (C_6), 28.1 ($C(\underline{C}H_3)_3$), 24.3 (C_5); IR 3294 (N-H), 2985 (C-H), 2241 (CN), 1743 (C=O ester), 1662 (C=O Boc); MS m/z 287 [M+Na], 265 $[MH]^+$, 209 $[MH - CH_2{=}CMe_2]^+$, 165 $[MH-CO_2, - CH_2{=}CMe_2]^+$; HRMS for $[MH]^+$ calculated 265.1188, found 265.1191.

<u>*Cycloaddition with styrene*</u>: A stirred solution of 5-NHBoc-2-pyrone (100 mg, 0.47 mmol) in styrene (2 mL) and a few crystals of BHT in a sealed tube was immersed in an oil bath maintained at 100 °C for 24 hours. The residue was subjected to silica gel column chromatography, using 50% v/v diethyl ether in hexane as eluent to afford a mixture of 5-*endo* and 5-*exo* cycloadduct (100 mg, 67%). The mixture of products was subjected to

recrystallisation using diethyl ether/hexane to afford a few crystals of the *endo* cycloadduct (10 mg, 7%).

5-*Endo* cycloadduct **14a**: Mp 87-90 °C; R_f = 0.27 (50% diethyl ether in hexane); ^{1}H NMR δ 7.26 – 7.20 (m, 2H, aromatic para), 7.19 – 7.13 (m, 1H, aromatic ortho), 7.12 – 7.05 (m, 2H, aromatic meta), 6.21 (s, 1H, NH), 5.74 (d, *J* = 6.4 Hz, 1H, H_8), 5.29 (s, 1H, H_1), 3.46 (dd, *J* = 6.7, 2.3 Hz, 1H, H_4), 3.35 (ddd, *J* = 9.7, 4.8, 2.4 Hz, 1H, H_{5exo}), 2.68 (ddd, *J* = 13.9, 9.7, 4.2 Hz, 1H, H_{6exo}), 1.99 (ddd, *J* = 13.9, 4.7, 1.4 Hz, 1H, H_{6endo}), 1.44 (s, 9H, $C(CH_3)_3$); ^{13}C NMR δ 173.8 (C_3), 152.2 (O=C-N), 141.6 (aromatic ortho), 139.5 (C_7), 128.6 (aromatic para), 127.7 (aromatic meta), 127.2 (aromatic ipso), 104.4 (C_8), 81.7 ($\underline{C}Me_3$), 76.4 (C_1), 46.5 (C_4), 37.6 (C_5), 34.0(C_6), 28.3 ($C(\underline{C}H_3)_3$); IR 3319 (N-H), 3030 (C=C-H) 2978 (C-H), 1748 (C=O ester), 1655 (C=O Boc); MS m/z 338 [M+Na], 316.5 $[MH]^+$, 260 $[MH - CH_2{=}CMe_2]^+$, 216 $[MH\text{-}CO_2, - CH_2{=}CMe_2]^+$; HRMS for $[MH]^+$ calculated 316.1549, found 316.1589.

5-*Exo* cycloadduct **14b** (identified from a mixture with 5-endo cycloadduct): ^{1}H NMR δ 7.17 (m, 5H, Phenyl group), 6.35 (s, 1H, NH), 6.16 (s, 1H, H_8), 5.14 (s, 1H, H_1), 3.33 (m overlapped with the H_4 of the endo isomer, 1H, H_4), 3.17 (ddd, J = 10.4, 5.8, 2.2 Hz, 1H, H_{5endo}), 2.34 (ddd, J = 13.9, 10.5, 1.7 Hz, 1H, H_{6endo}), 2.25 (ddd, J = 14.2, 5.9, 3.7 Hz, 1H, H_{6exo}), 1.43 (s, 9H, $C(CH_3)_3$).

Cycloaddition with vinyl acetate: A solution of 5-NH(Boc)-2(H)pyran-2-one **8** (380 mg, 1.79 mmol) in vinyl acetate (40 mL, large excess), was refluxed (75 °C) under a dry nitrogen atmosphere for 10 days. All volatile materials were removed under reduced pressure. Proton nuclear magnetic resonance of the crude residue indicated the presence of three isomeric cycloadducts in the ratio of 2 (5-endo): 5.25 (5-exo): 1 (6-endo). The residue was dissolved in a minimum quantity of hot diethyl ether, and a few drops of hexane were added until the solution turned turbid and left for recrystallisation. The solid was filtered to afford pure 5-exo cycloadduct as a white solid (320 mg, 60%). The supernatant was subjected to silica gel chromatography, using 50% v/v diethyl ether in hexane as eluent to afford Mix of 5-exo and 5-endo cycloadducts (1:2) (110 mg, 20%) and the 6-endo cycloadduct (30 mg, 2%).

5-*Endo* cycloadduct of **15a:** (identified from a mixture with 5-exo cycloadduct); 1H NMR δ 6.36 (s, 1H, NH), 5.92 (d, J = 4.9 Hz, 1H, H_8), 5.26 (broad s, 1H, H_1), 5.18 (dddd, J = 8.2, 3.3, 2.3, 0.9 Hz, 1H, H_{5exo}), 3.80 (ddd, J = 6.5, 3.4, 0.7 Hz, 1H, H_4), 2.75 (ddd, J = 14.7, 8.2, 3.8 Hz, 1H, , H_{6exo}), 2.06 (s, 3H, CH_3), 1.79 (dddd, J = 14.7, 2.4, 1.6, 0.7 Hz, 1H, H_{6endo}), 1.51 (s, 9H, $C(CH_3)_3$).

5-*Exo* cycloadduct of **15b**: Mp 159-161 °C; R_f = 0.12 (50% diethyl ether in hexane); ^{1}H NMR δ 6.32 (s, 1H, NH), 5.97 (d, *J* = 5.6 Hz, 1H, H_8), 5.17 – 5.11 (m, 1H, H_1), 5.08 (dtd, *J* = 9.1, 3.2, 0.6 Hz, 1H, H_{5endo}), 3.66 (dd, *J* = 7.1, 3.3 Hz, 1H, H_4), 2.45 (ddd, *J* = 14.5, 9.2, 1.6 Hz, 1H, H_{6endo}), 2.09 (s, 3H, CH_3), 2.04 (dt, *J* = 14.5, 3.3 Hz, 1H, H_{6exo}), 1.50 (s, 9H, $C(CH_3)_3$); ^{13}C NMR δ 171.0 (C_3), 170.5 (O=C-O), 151.9 (O=C-N), 140.7 (C_7), 101.8 (C_8), 81.9 ($\underline{C}(CH_3)_3$), 75.4 (C_1), 68.2 (C_5), 44.5 (C_4), 33.6 (C_6), 28.2 ($C(\underline{C}H_3)_3$), 21.0 (CH_3); IR (KBr disk) 3308 (N-H), 2990 (C-H), broad band 1744 (C=O ester), 1661 (C=O Boc). MS m/z 320 $(M+Na)^+$, 298 $(MH)^+$, 242 $[MH -CH_2{=}CMe_2]^+$, 198 $[MH -CO_2, - CH_2{=}CMe_2]^+$; HRMS for $[MH]^+$ calculated 298.12906, found 298.12952.

6-*Endo* cycloadduct of **15c**: Viscous liquid; R_f = 0.17 (50% diethyl ether in hexane); ^{1}H NMR δ 6.24 (s, 1H, NH), 6.22 (s, 1H, H_8), 5.24 (ddd, *J* = 8.0, 4.4, 3.3 Hz, 1H, H_{6exo}), 5.11 (dd, *J* = 4.4, 2.8 Hz, 1H, H_1), 3.35 (dt, *J* = 7.0, 2.8 Hz, 1H, H_4), 2.37 (ddd, *J* = 13.7, 8.3, 2.8 Hz, 1H, H_{5exo}), 1.97 (s, 3H, CH_3), 1.49 (dt, *J* = 13.6, 3.0 Hz, 1H, H_{5endo}), 1.42 (s, 9H, $C(CH_3)_3$); ^{13}C NMR (101 MHz, $CDCl_3$) δ 172.4 (C_3), 170.3 (O=C-O), 152.0 (O=C-N), 136.0 (C_7), 106.6 (C_8), 81.6 ($\underline{C}(CH_3)_3$), 74.8 (C_1), 68.2

(C_6), 38.2 (C_4), 29.5 (C_5), 28.2 (C(<u>C</u>H_3)$_3$), 20.8 (CH_3); IR 3329 (N-H), 2980 (C-H), broad band 1742 (C=O ester), 1657 (C=O Boc). MS m/z 320 $(M+Na)^+$, 298 $(MH)^+$, 242 $[MH -CH_2=CMe_2]^+$, 198 [MH $-CO_2$, - $CH_2=CMe_2]^+$; HRMS for $[MH]^+$ calculated 298.12906, found 298.12933.

<u>*Cycloaddition with Vinylene Carbonate*</u>: A stirred solution of 5-NHBoc-2-pyrone (150 mg, 0.47 mmol) in Vinylene Carbonate (2 mL) in a sealed tube was immersed in an oil bath maintained at 100 °C for 24 hours. The reaction mixture was subjected to silica gel column chromatography, using 60% v/v diethyl ether in hexane as eluent to afford 5-*endo* cycloadduct (72 mg, 34%) and 5-*exo* cycloadduct (35 mg, 17%).

5-*Endo* cycloadduct **16a**: Mp 188-189 °C; R_f = 0.18 (60% diethyl ether in hexane); ^{1}H NMR (Acetone-d_6) δ 8.49 (s, 1H, NH), 6.09 (d, *J* = 9.7 Hz, 1H, H_8), 5.51 (dd, *J* = 4.4, 2.7 Hz, 1H, H_1), 5.27 (dd, *J* = 7.7, 4.4 Hz, 1H, H_{6exo}), 5.16 (ddd, *J* = 7.7, 3.7, 0.9 Hz, 1H, H_{5exo}), 3.87 (dd, *J* = 6.8, 3.8 Hz, 1H, H_4), 1.33 (s, 9H, C(CH_3)$_3$); ^{13}C NMR (Acetone-d_6) δ 167.80, 153.55, 152.19, 137.33, 100.15, 80.53, 73.68, 72.50, 71.15, 43.26, 27.41; IR 3349 (N-H), 2968 (C-H), 1779 (C=O Carbonate), 1762 (C=O ester), 1662 (C=O Boc); MS m/z 320 $(M+Na)^+$, 298 $(MH)^+$, 242 $[MH -CH_2=CMe_2]^+$, 198 [MH $-CO_2$, $-CH_2=CMe_2]^+$; HRMS for $[M-H]^-$ calculated 296.07758, found 296.07816.

5-Exo cycloadduct **16b**: Mp decomposed stating at 186 °C; R_f = 0.09 (60% diethyl ether in hexane); ^{1}H NMR (Acetone-d_6) δ 8.45 (s, 1H, NH), 6.01 (dd, J = 7.1, 2.4 Hz, 1H, H_8), 5.43 (t, J = 2.2 Hz, 1H, H_1), 5.11 (dd, J = 8.5, 2.0 Hz, 1H, H_{6endo}), 5.04 (ddd, J = 8.5, 3.9, 0.6 Hz, 1H, H_{5endo}), 3.85 (dd, J = 7.1, 3.9 Hz, 1H, H_4), 1.33 (s, 9H, C(CH_3)$_3$); ^{13}C NMR (Acetone-d_6) δ 168.26, 153.46, 152.26, 138.19, 100.73, 80.68, 75.65, 73.62, 72.29, 43.73, 27.41; IR 3394 (N-H), 2926 (C-H), 1794 (C=O Carbonate), 1762 (C=O ester), 1657 (C=O Boc); MS m/z 320 $(M+Na)^+$, 242 [MH - $CH_2=CMe_2]^+$, 198 [MH $-CO_2$, - $CH_2=CMe_2]^+$; HRMS for $[M-H]^-$ calculated 296.07758, found 296.07770.

Dimer of 5-(BocNH)-2(H)pyran-2-one, **17**: A solution of 5-(BocNH)-2(H)pyran-2-one 7 (100 mg, 0.47 mmol) in ethyl acetate (2 mL) was heated at reflux (77 °C) under a dry nitrogen atmosphere for 15 hours. After cooling, the solid was filtered and air dried (88 mg, 88%).

Mp 175 °C; ^{1}H NMR (DMSO-d_6) δ 9.56 (s, 1H, NH), 7.81 (s, 1H, NH), 6.47 (1H, d, J = 10 Hz, C=C<u>H</u>-CO), 6.02 (1H, d, J = 10 Hz, C<u>H</u>=C-CO), 5.94 (1H, d, J = 7 Hz, H_7), 5.30 (1H, dd, J = 4.3, 2.7 Hz, H_1), 5.15 (1H, d, J = 4.2 Hz, H_5), 3.88 (1H, m, H_4), 1.40 (9H, s, 3xCH_3), 1.37 (9H, s, 3xCH_3); ^{13}C NMR (DMSO-d_6) δ 169.7 (CO_2 bridge aka C_3), 159.7 (CO_2), 155.3 (O=C-N), 152.5 (O=C-N), 140.8 (<u>C</u>=C-CO), 139.1 (N<u>C</u>=C aka C_7), 120.3 (C=<u>C</u>-CO), 101.4 (NC=<u>C</u> aka C_8), 80.6 (C_5), 80.0 (2xCMe_3), 75.3 (C_1), 52.4 (C_6), 46.5 (C_2), 28.4 (3xCH_3), 28.3 (3xCH_3); IR 3331 (N-H), 3256 (N-H), 2980 (C-H), 1771 (C=O pyrone), 1760 (C=O), 1635 (C=O Boc). MS m/z 322 [MH $-CO_2$, $-CH_2=CMe_2]^+$, 213 $(monomer+H)^+$, 113 [monomer-CO_2, - $CH_2=CMe_2]^+$; HRMS for MH $-CO_2$, $-CH_2=CMe_2]^+$ calculated 322.1170, found 322.1165.

Experimental procedures for rate determination A stirred solution of 5-NHBoc-2-pyrone (100 mg, 0.47 mmol) in the corresponding dienophile (4 mL) in a sealed tube was immersed in an oil bath maintained at 100 °C. At the required time interval, a 0.5 mL aliquot was withdrawn for analysis, stripped of all volatiles and analysed. Tables below show the relative fraction of each component in the reaction mixture as determined from integration of ^{1}H NMR.

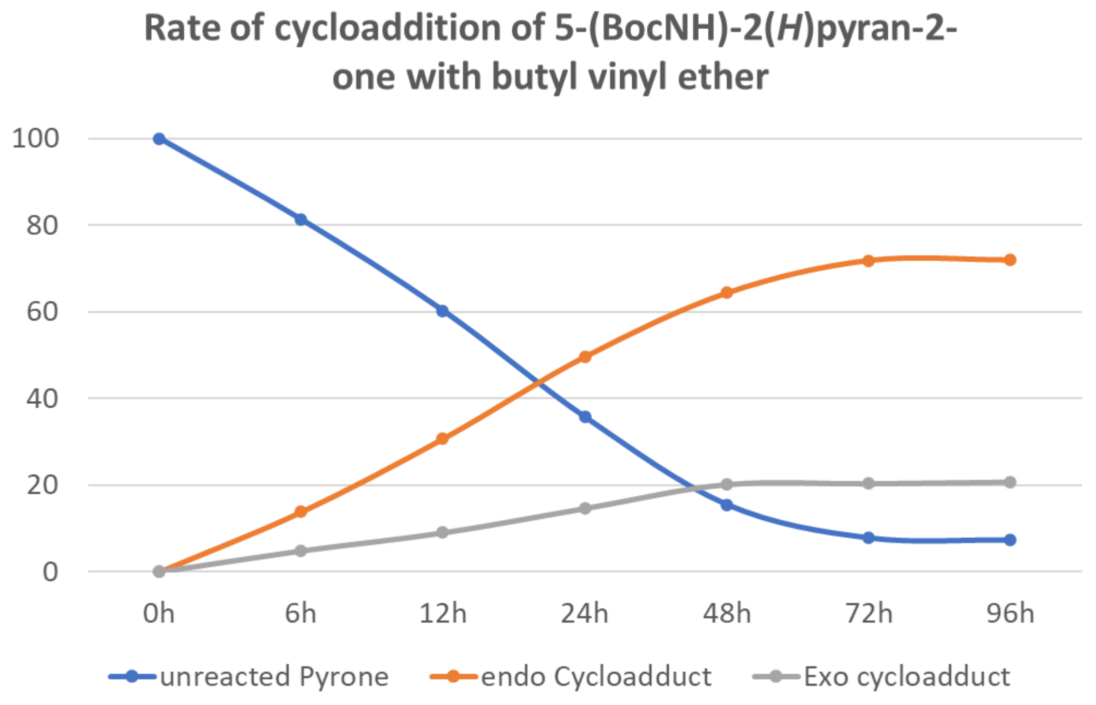

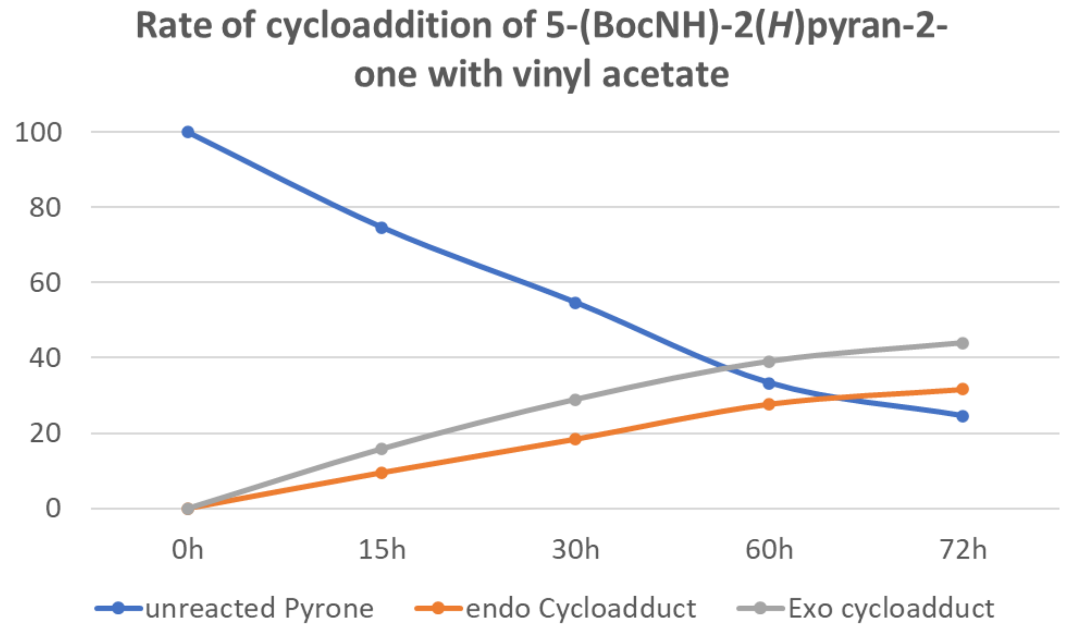

Guide to determining endo and exo isomers

R	5-endo isomer						5-exo isomer					
	$J_{4,5exo}$	$J_{5,6exo}$	$J_{5,6endo}$	$J_{1,6exo}$	$J_{1,6endo}$	δ_{5exo}	$J_{4,5endo}$	$J_{5,6exo}$	$J_{5,6endo}$	$J_{1,6exo}$	$J_{1,6endo}$	δ_{5endo}
CO_2Me	2.7	9.7	4.0	3.9	1.6	2.98	2.4	5.4	10.8	3.8	1.7	2.84
CN	2.6	9.7	3.7	3.8	1.6	3.12	2.4	5.4	10.8	3.8	1.7	2.84
Ph	2.4	9.7	4.8	4.2	1.4	3.35	2.2	5.8	10.4	3.7	1.7	3.17
OAc	3.4	8.2	2.4	3.8	1.6	5.18	3.3	3.3	9.2	3.4	1.6	5.08
O(CO)O	3.8	7.7	-	4.4	-	5.16	3.9	-	8.5	2.2	-	5.04
OBu	3.3	7.8	2.4	3.9	1.6	3.89	3.1	3.6	8.8	3.6	1.7	3.76

- In the *endo* cycloadduct, chemical shift of H_{5exo} > chemical shift of H_{5endo} in the *exo* cycloadduct.
- $J_{4,5exo}$ (in the *endo* cycloadduct) ≈ 2.4-3.8 Hz and is larger than $J_{4,5endo}$ (in the *exo* cycloadduct) ≈ 2.1-3.3 Hz
- The *endo* and *exo* protons at position 6 are distinguishable due to the size of their coupling to H_1: $J_{1,6exo}$ ≈ 3.3- 4.2 Hz is larger than $J_{1,6endo}$ ≈ 1.4-1.7 Hz
- In the *endo* cycloadduct, H_{6exo} has a large coupling (7.8-10.8 Hz) to H_{5exo} due to their *syn* relationship. In the *exo* cycloadduct, H_{6endo} has a larger coupling (7.8-10.8 Hz) to H_{5endo} due to their *syn* relationship.

^{1}H & ^{13}C NMR of **8.**

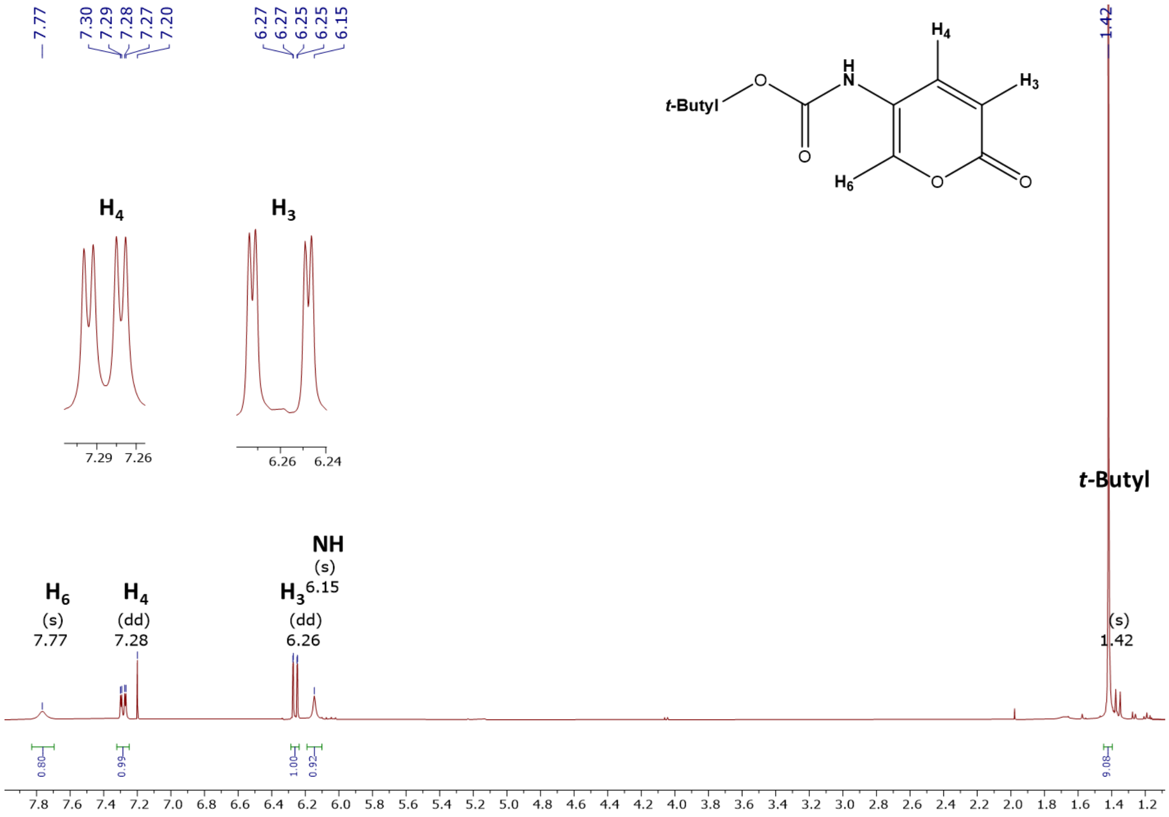

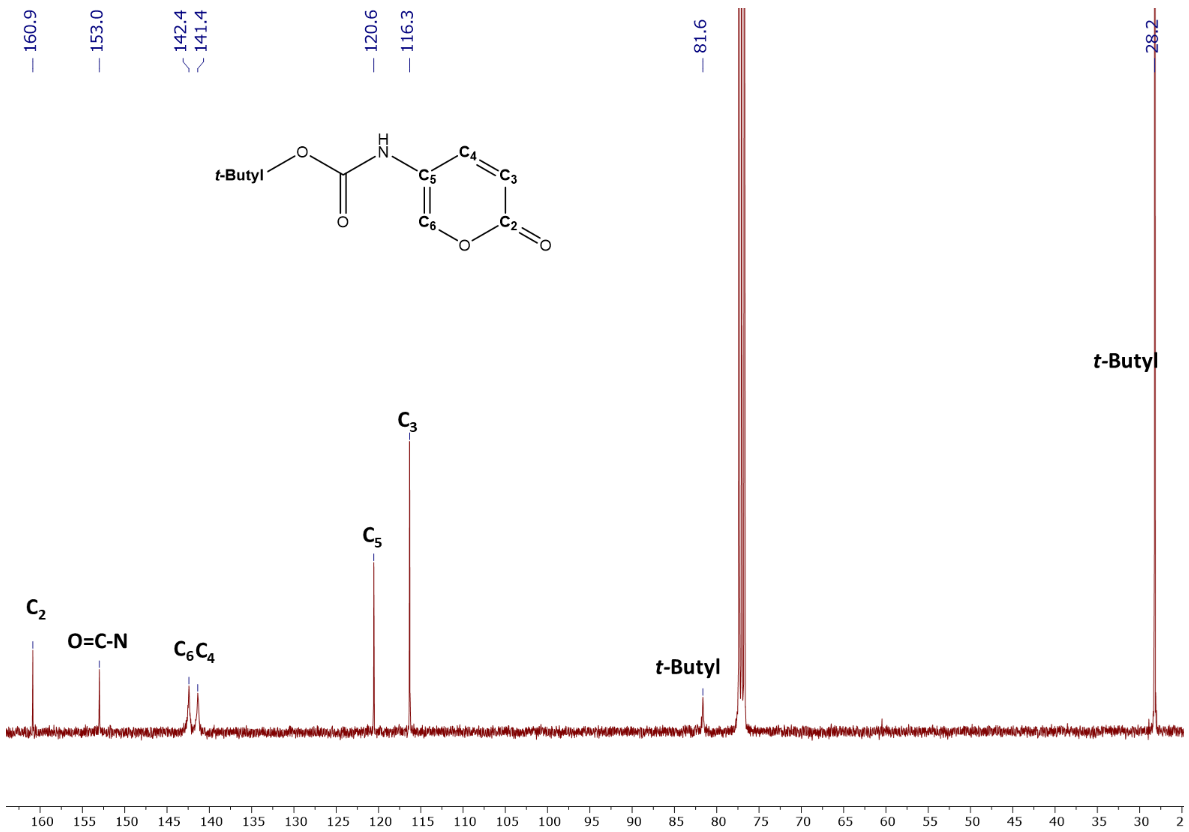

COSY & HSQC of **8.**

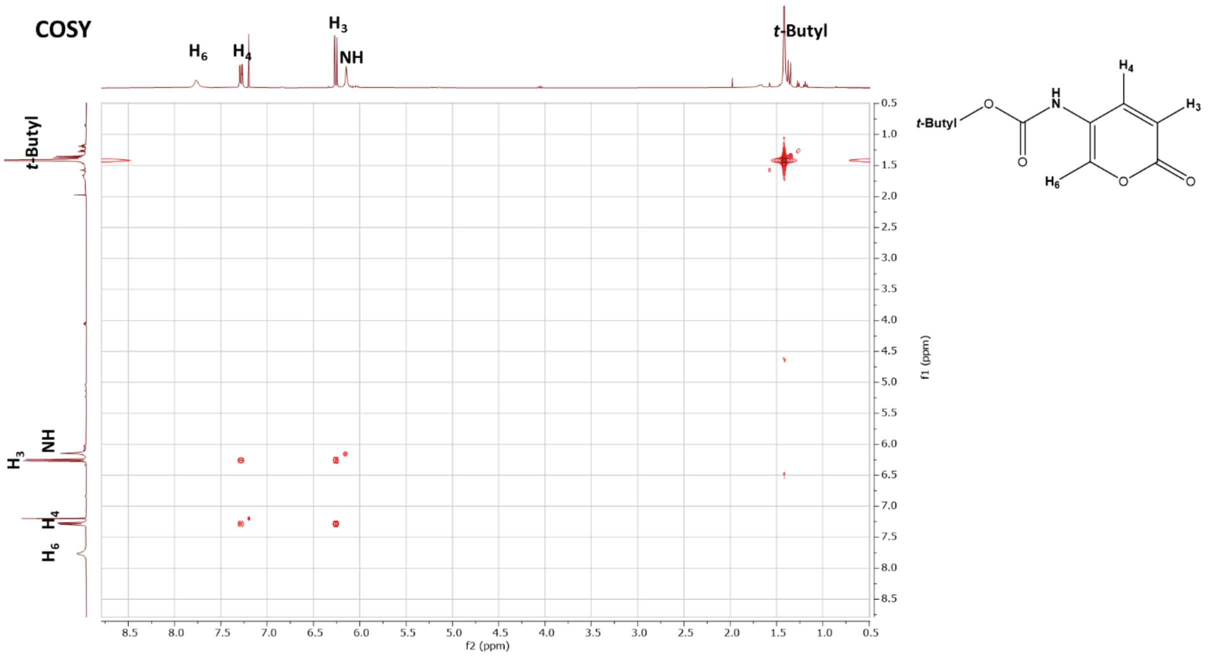

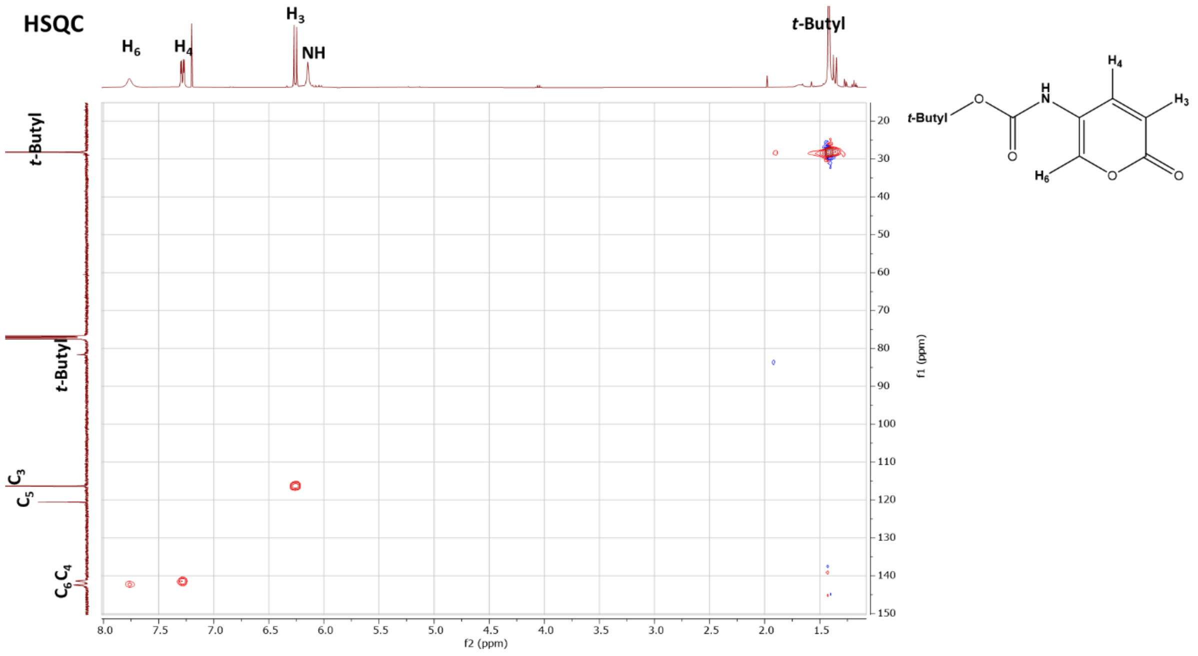

^{1}H & ^{13}C NMR of **10a.**

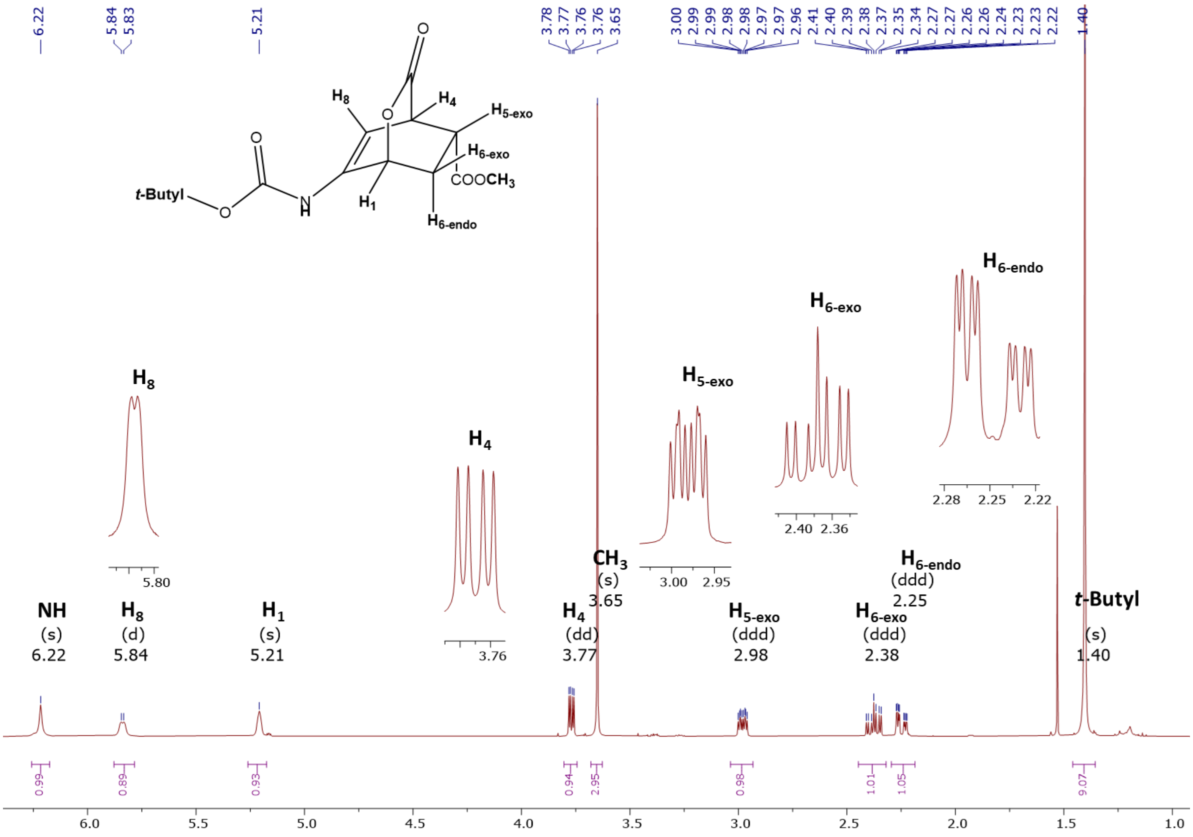

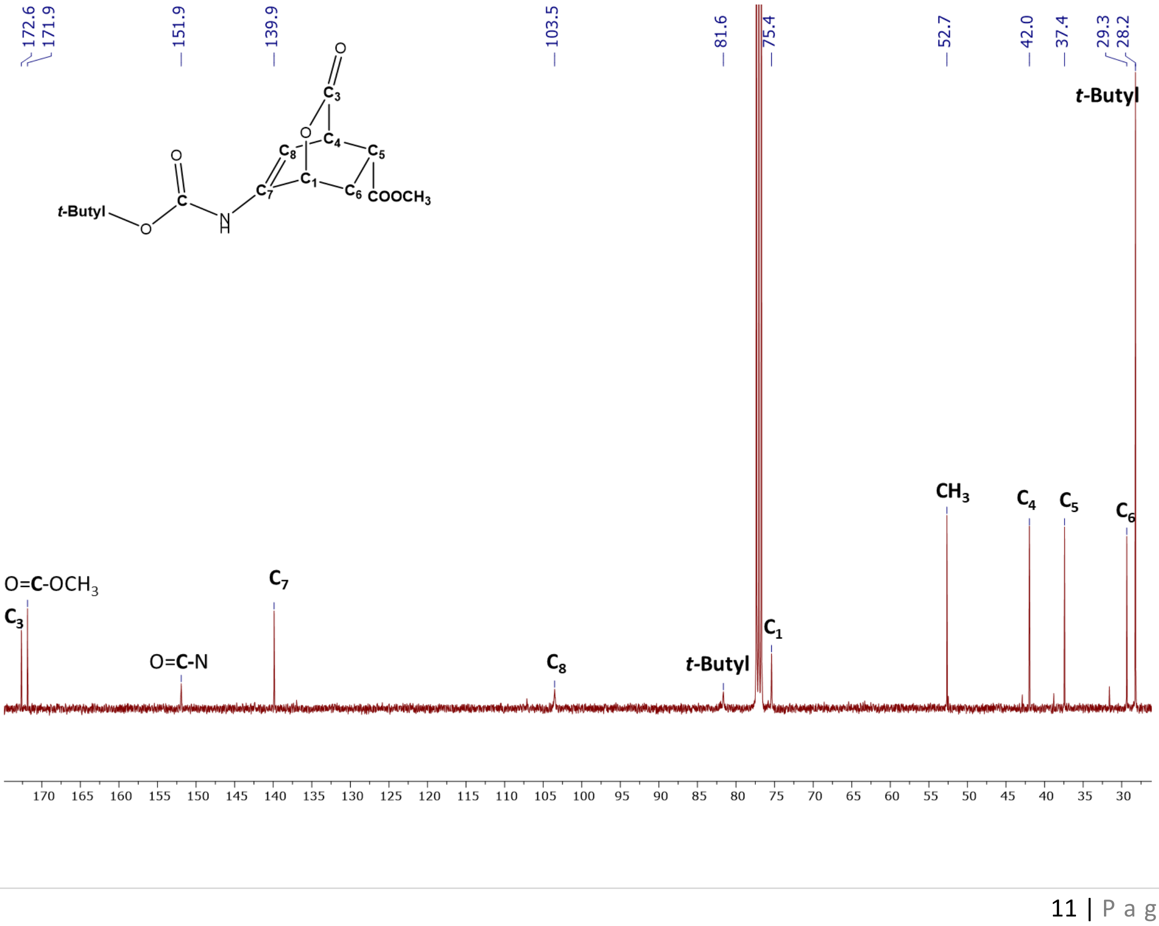

COSY & HSQC of **10a**.

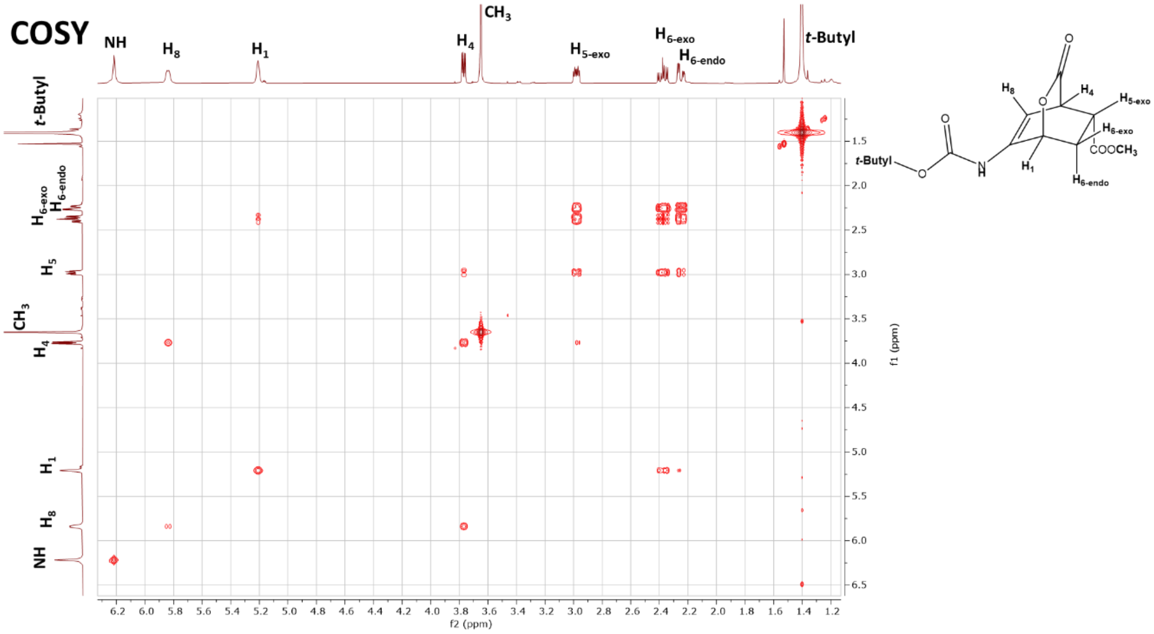

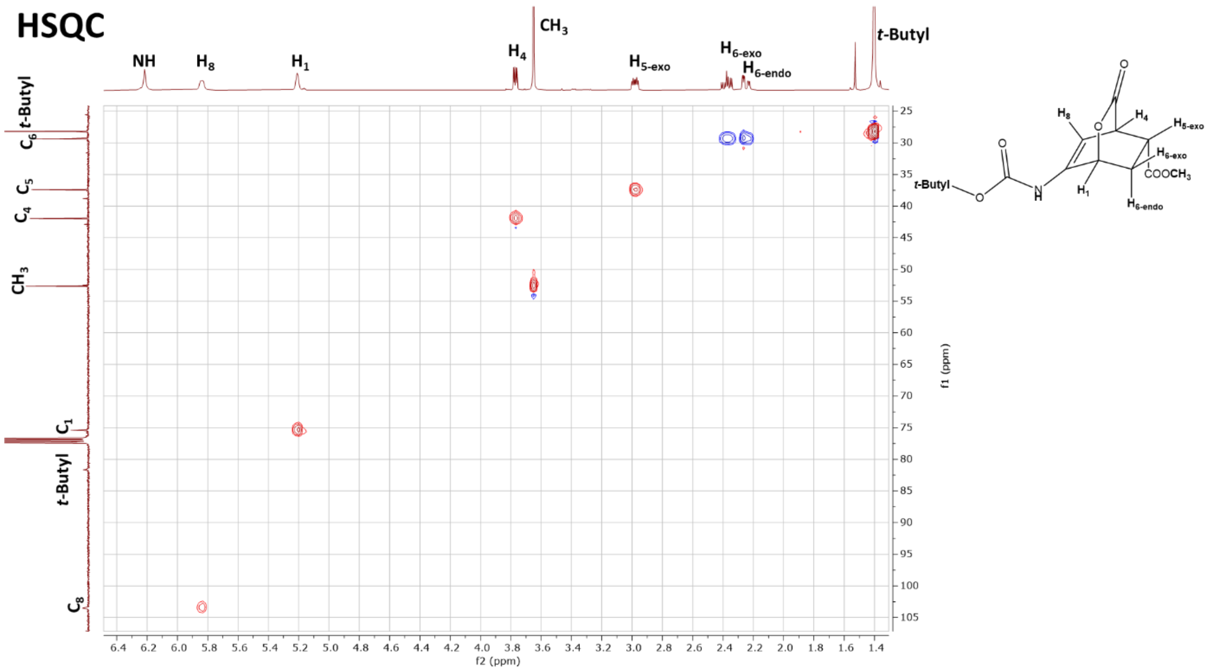

^{1}H & ^{13}C NMR of **10b**.

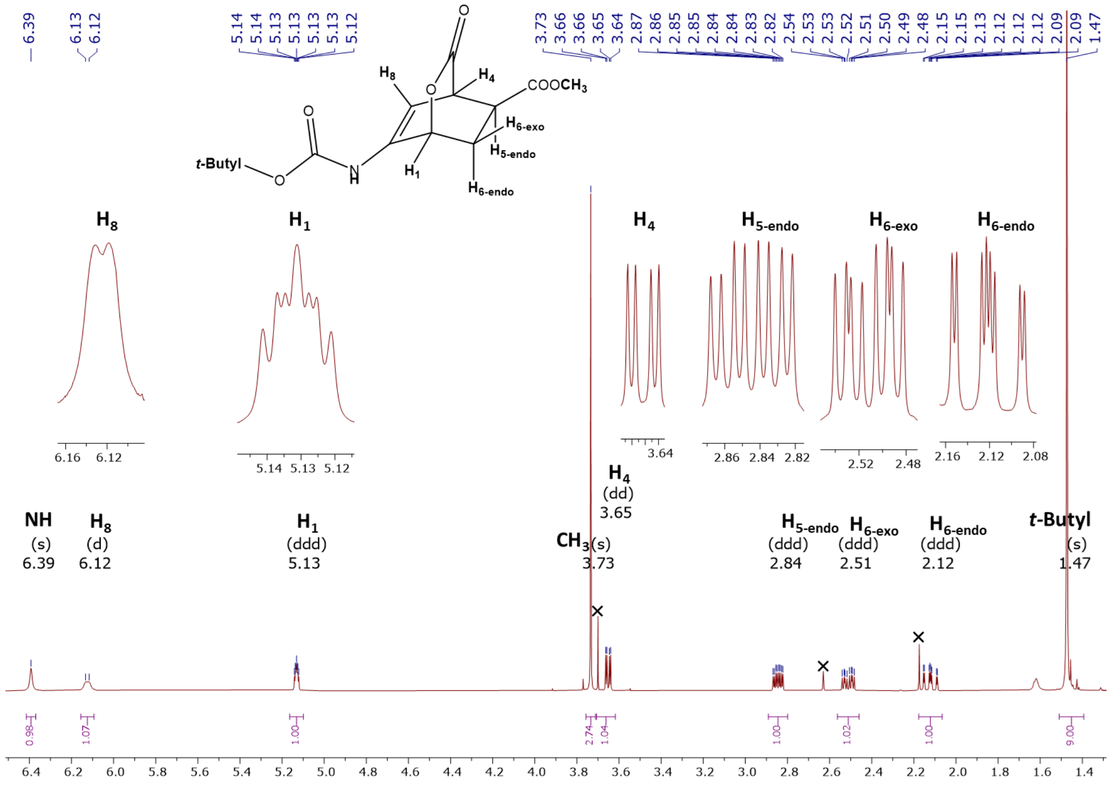

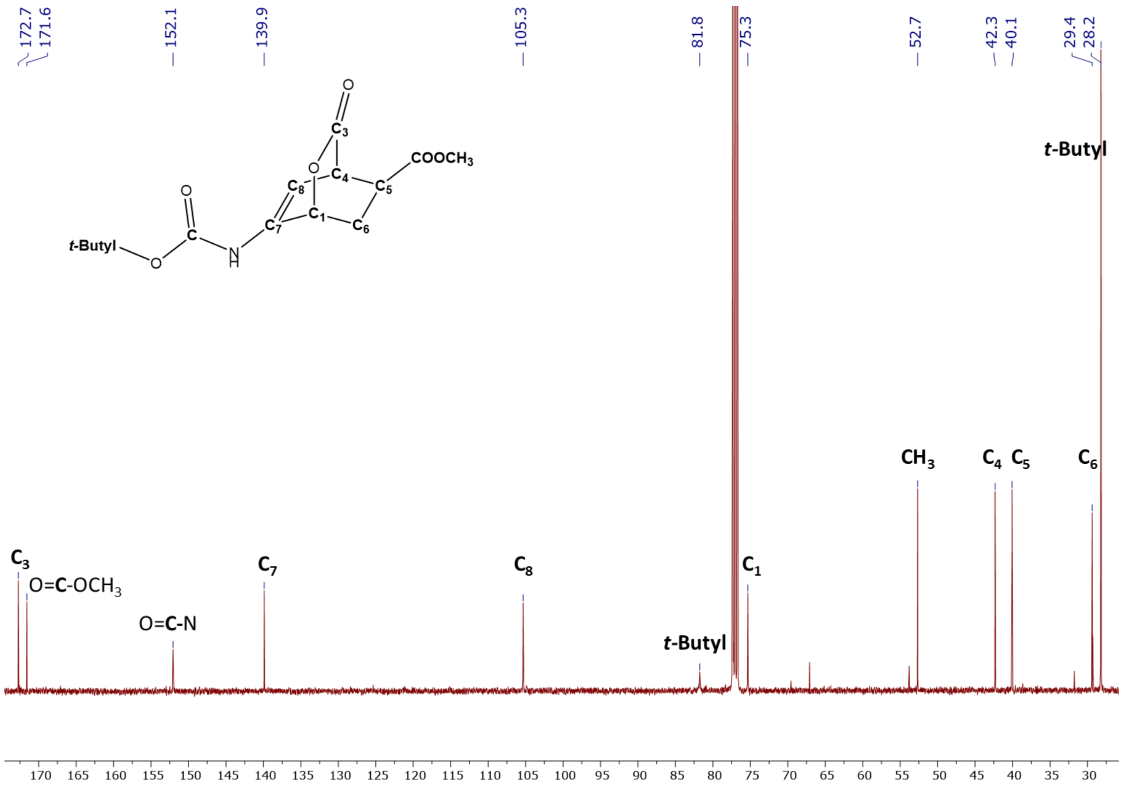

COSY & HSQC of **10b**.

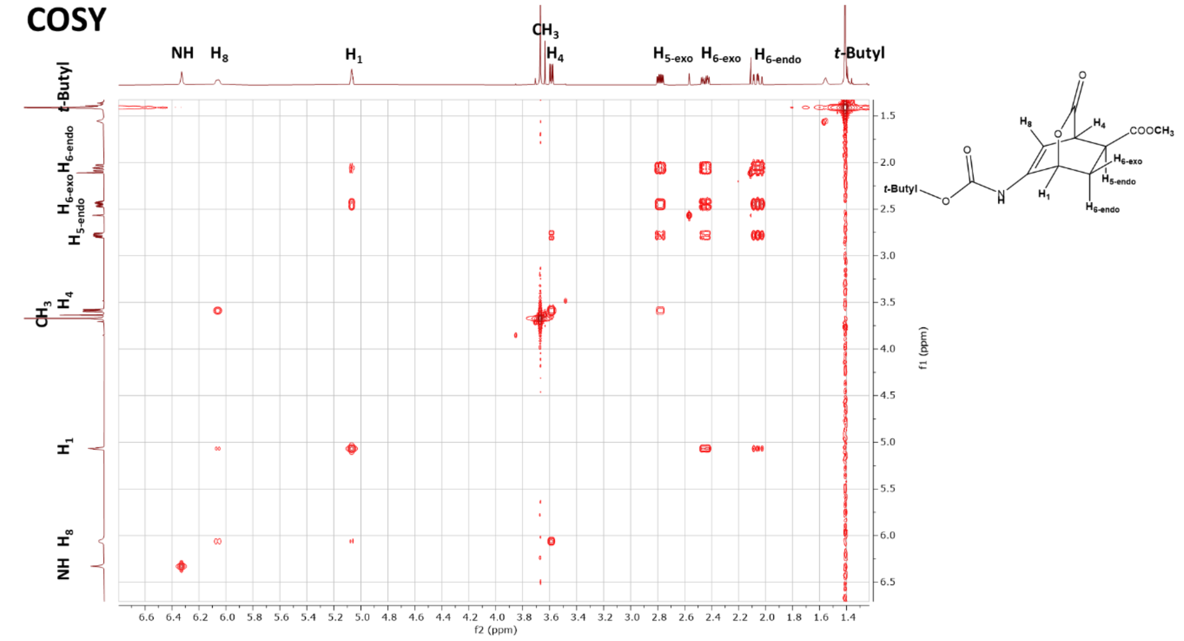

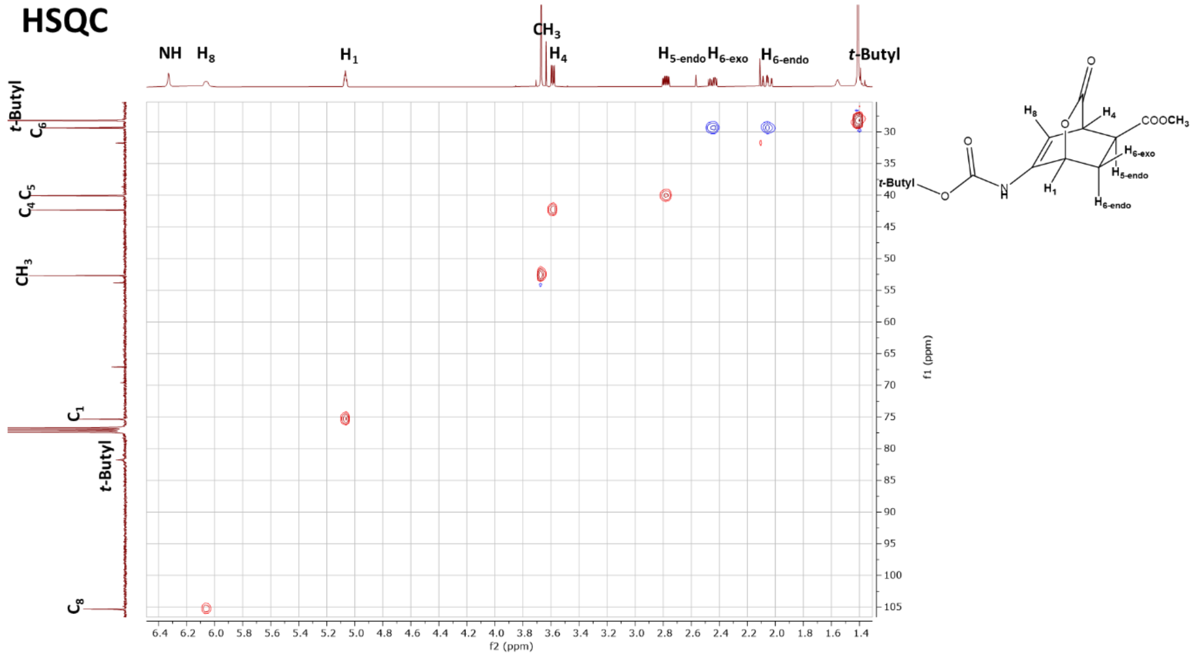

lc traces for **10a** and **10b**

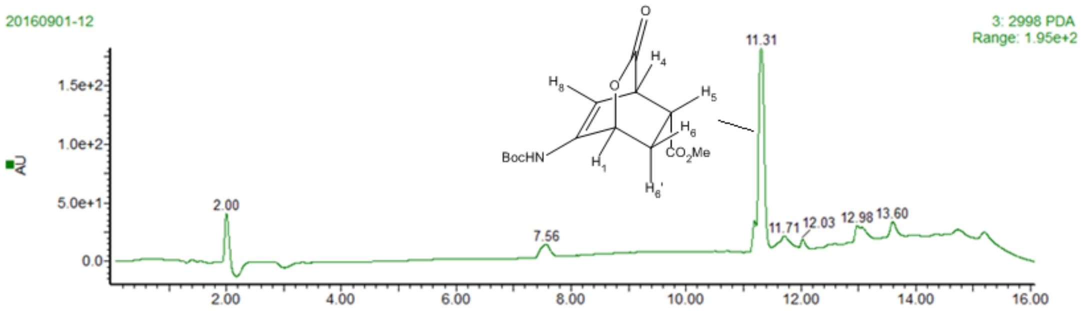

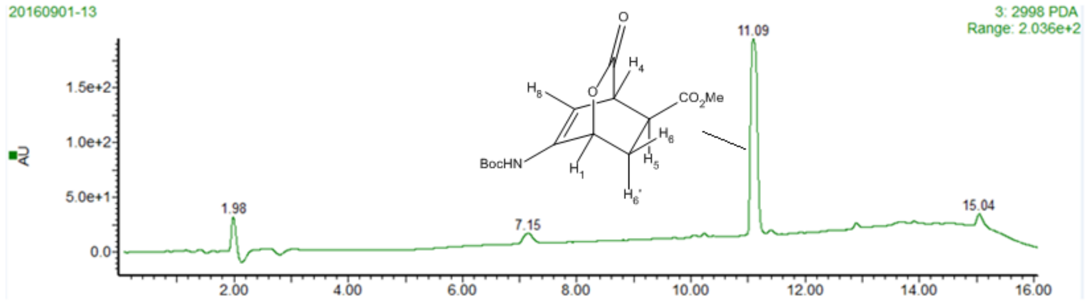

^{1}H & ^{13}C NMR of **11a**.

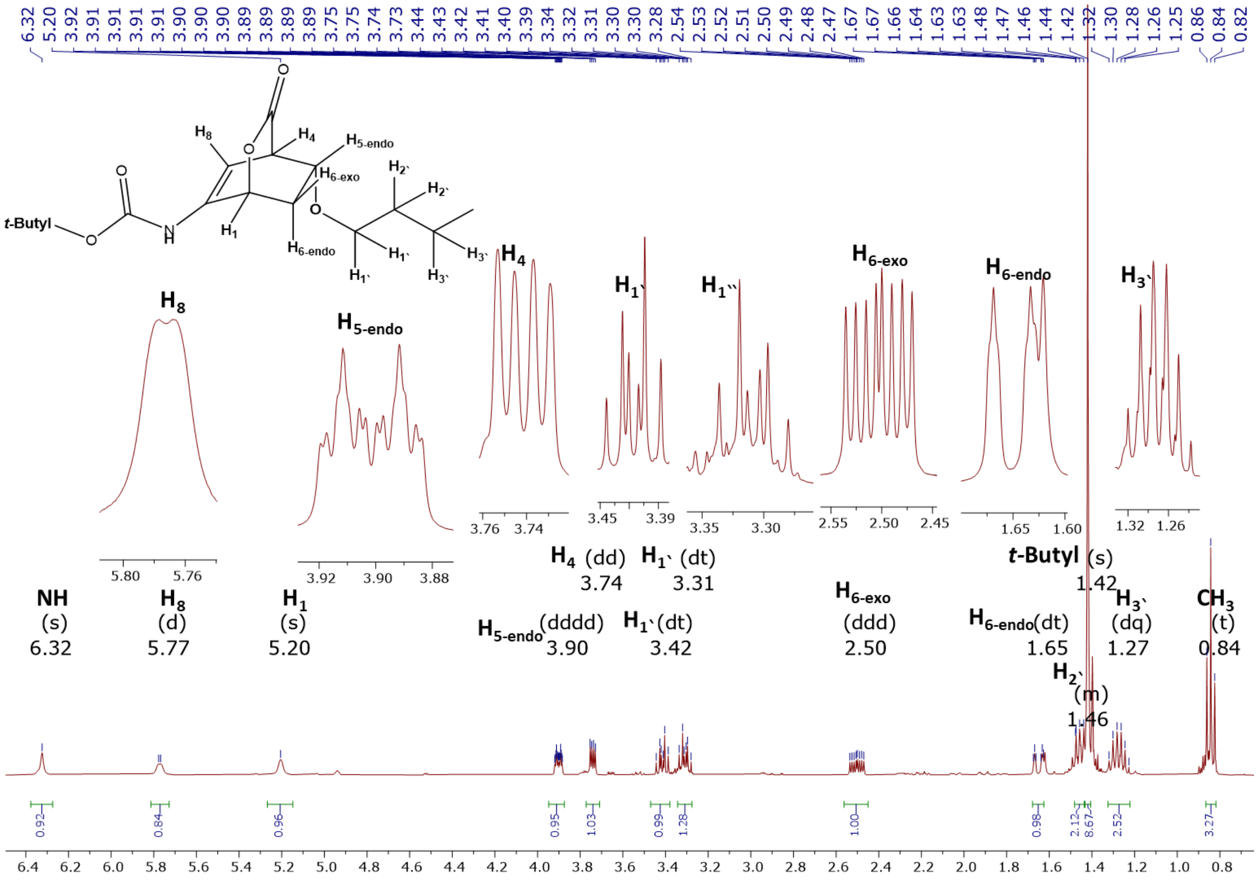

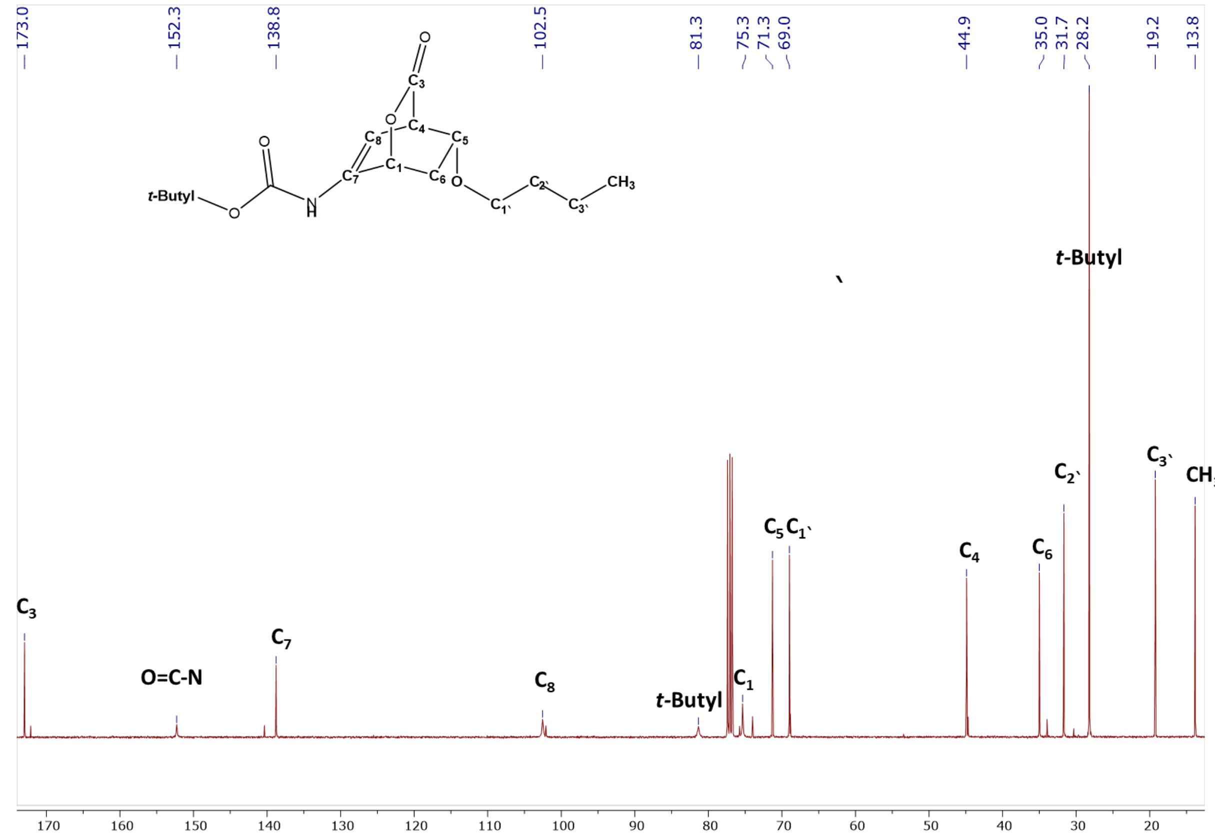

COSY & HSQC of **11a**.

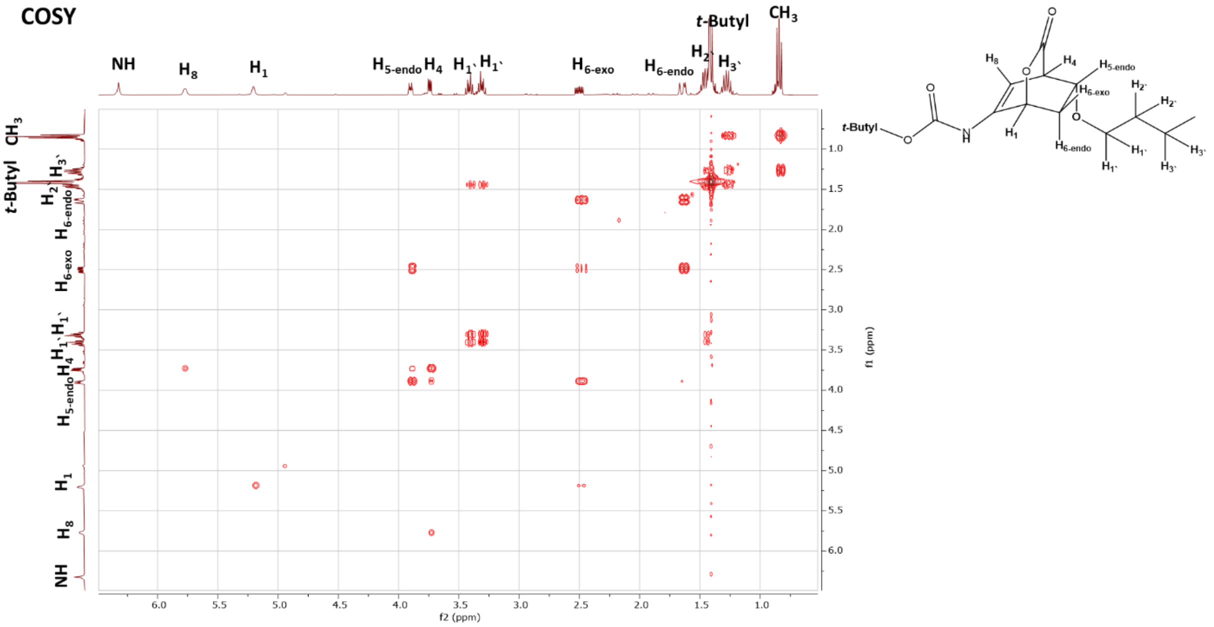

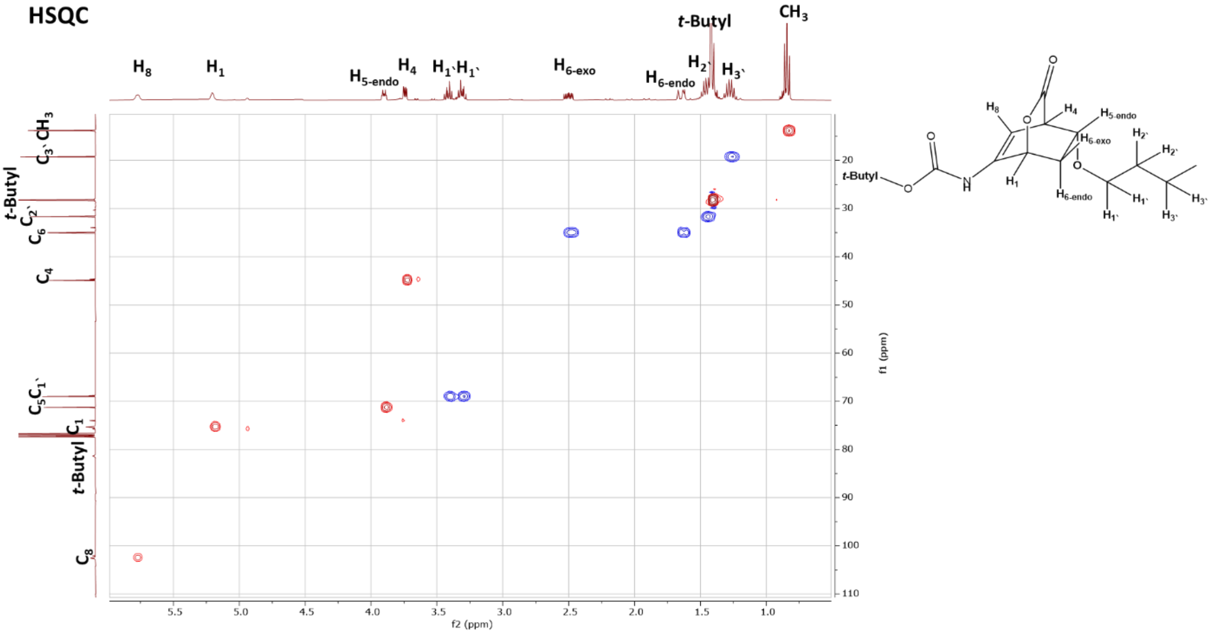

^{1}H & ^{13}C NMR of **11b**.

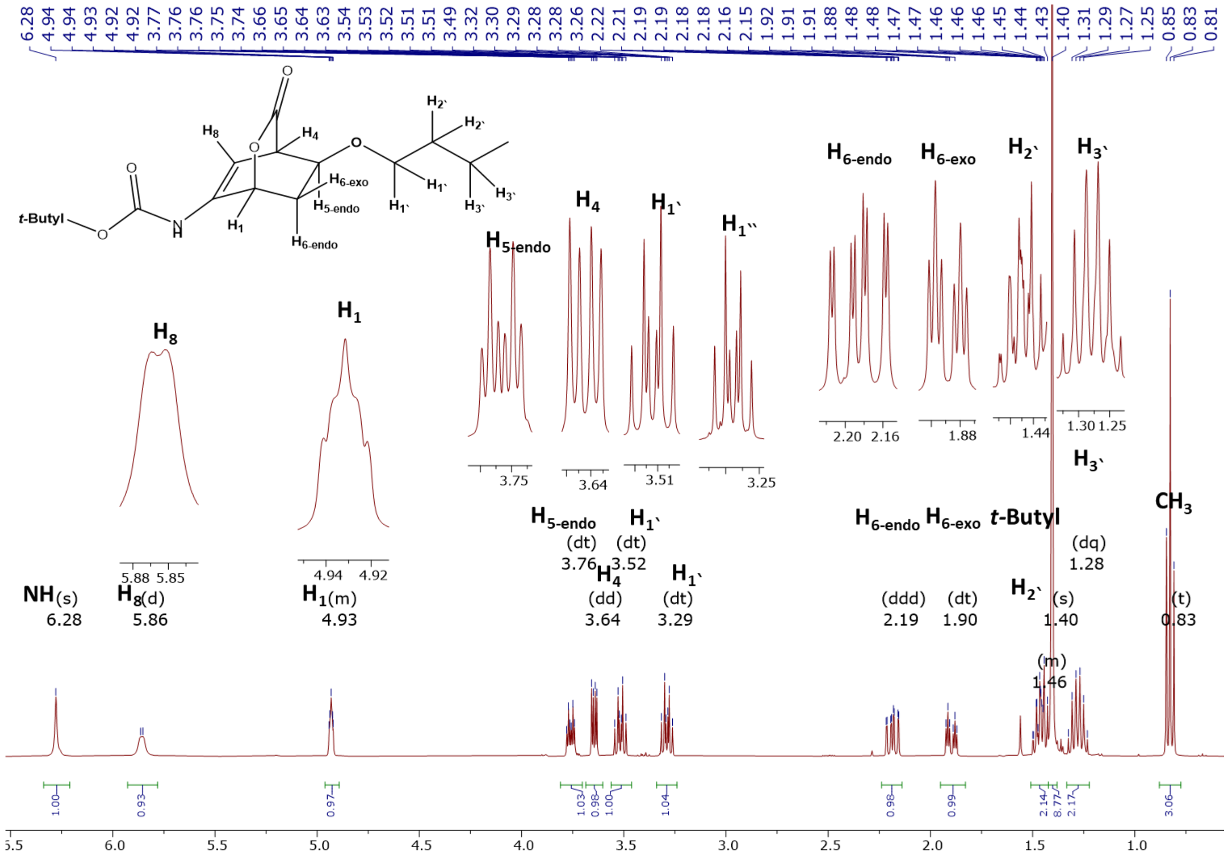

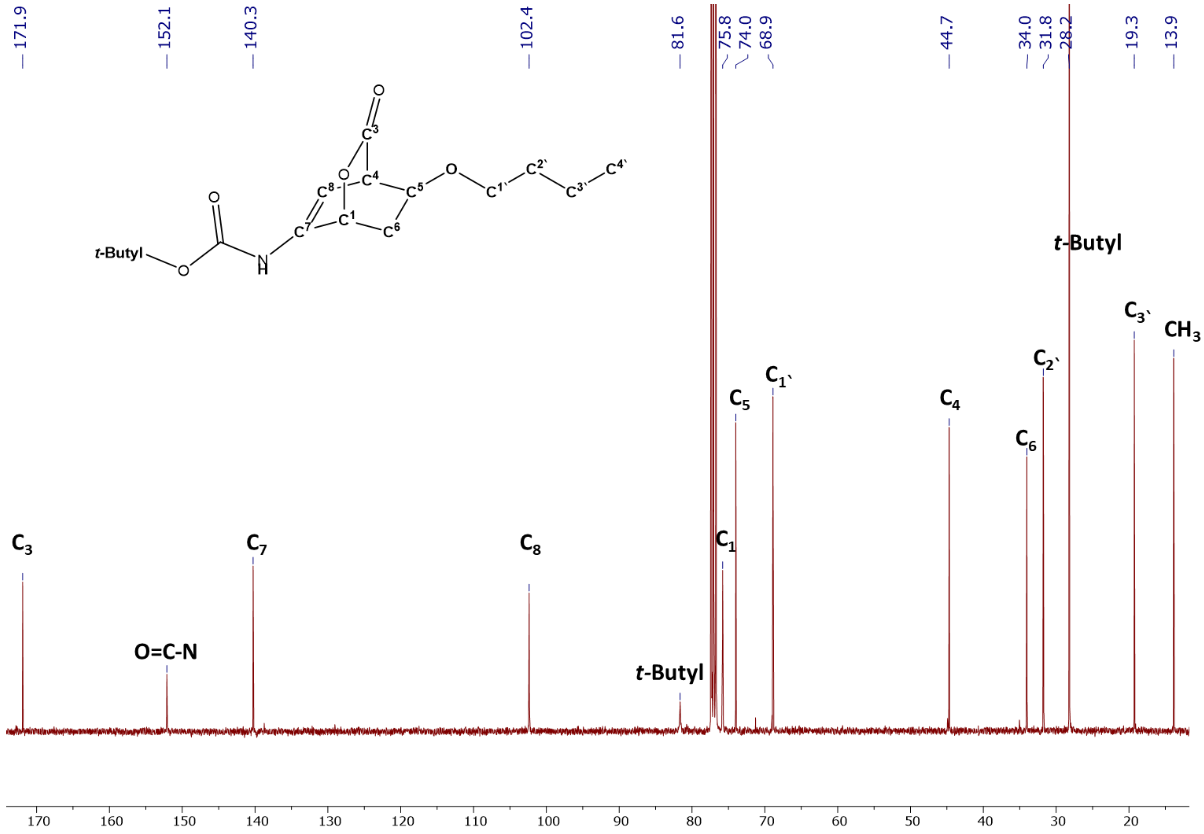

COSY and HSQC of **11b**.

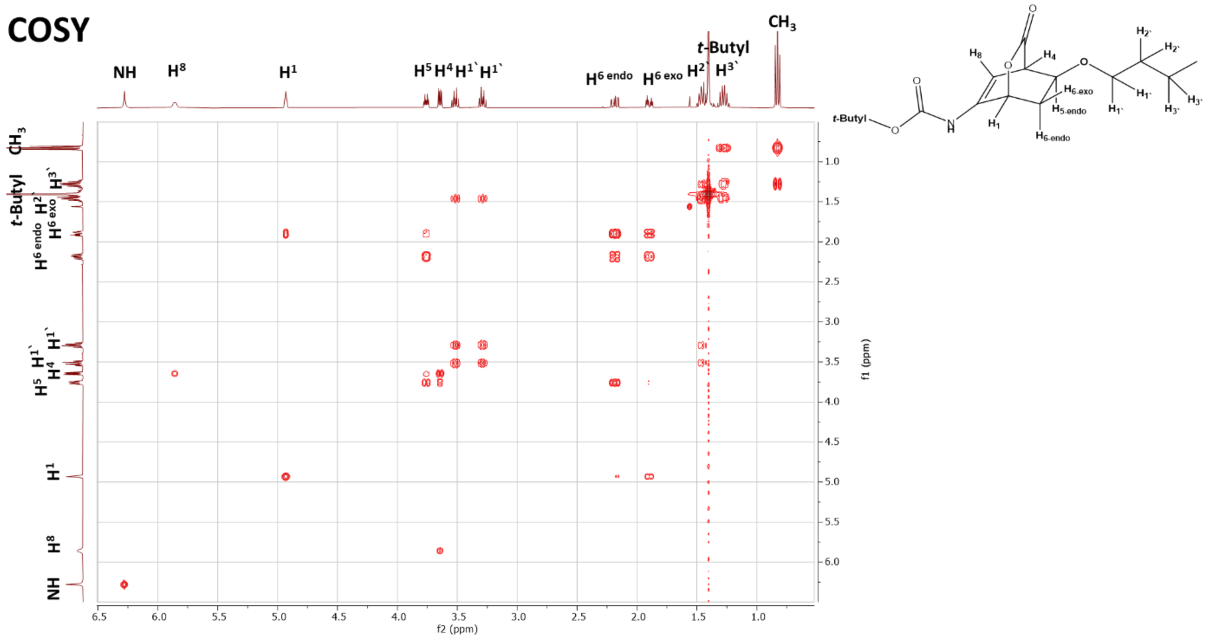

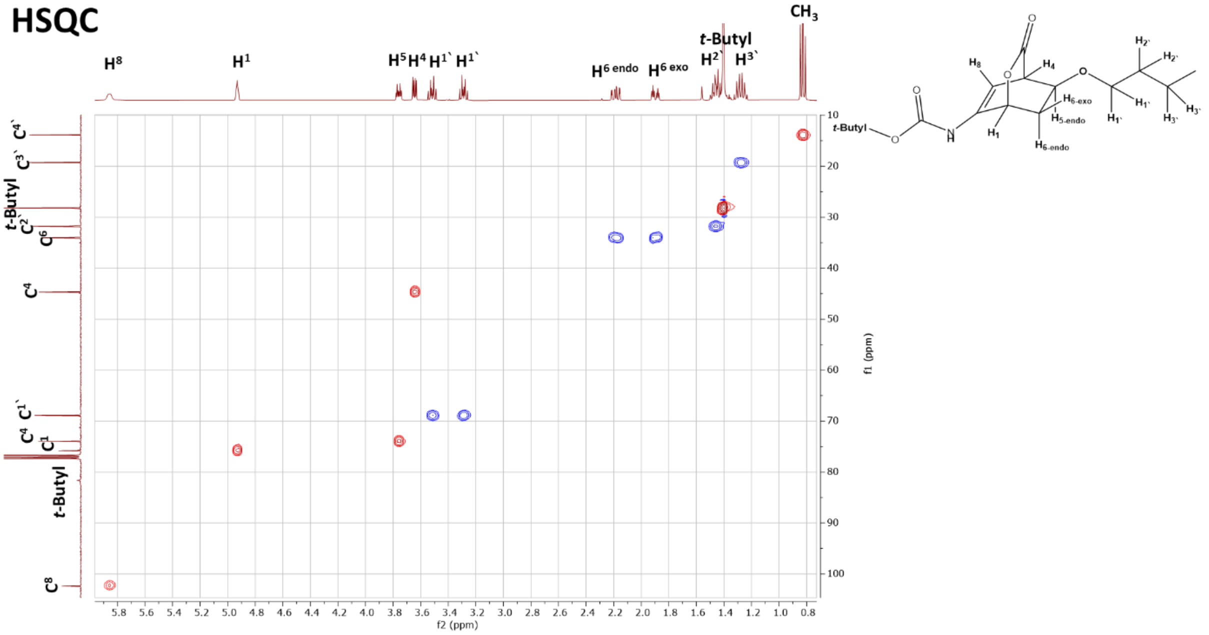

^{1}H & ^{13}C NMR of **12a**.

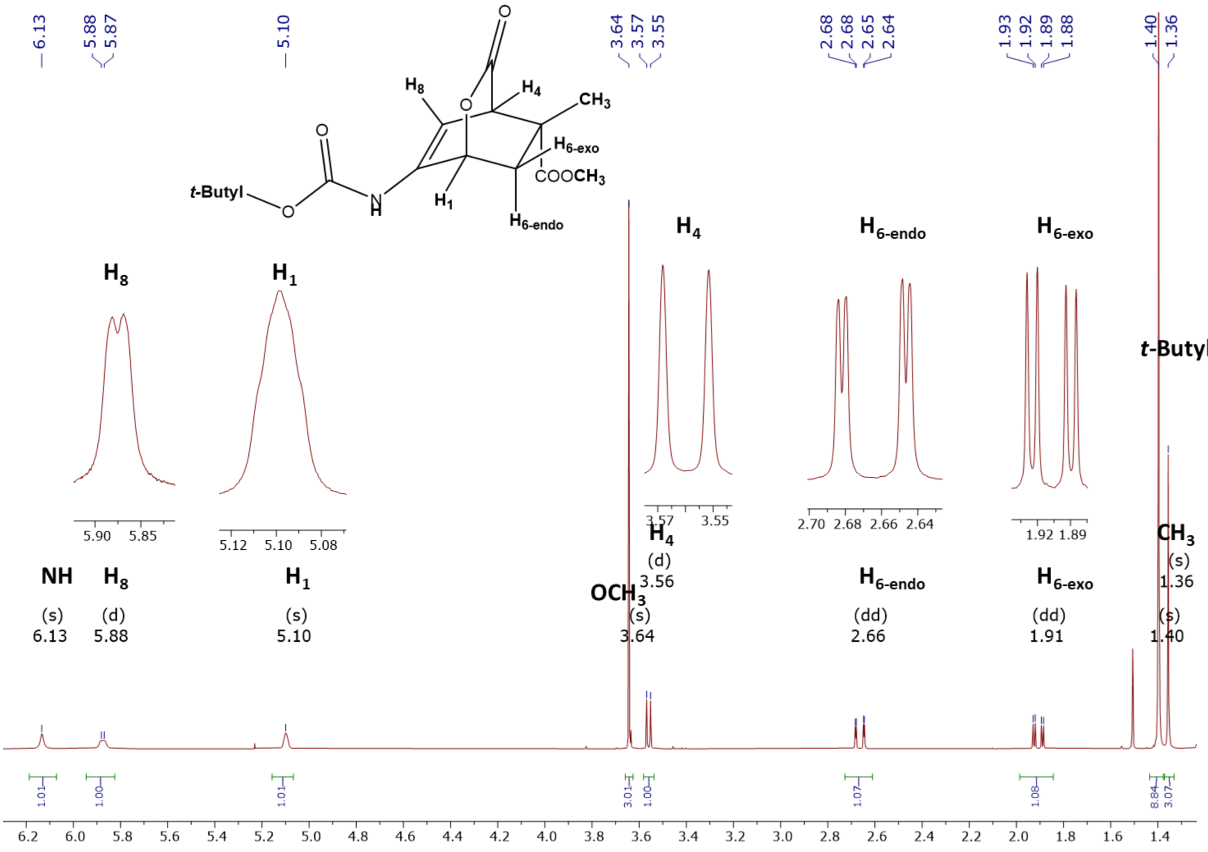

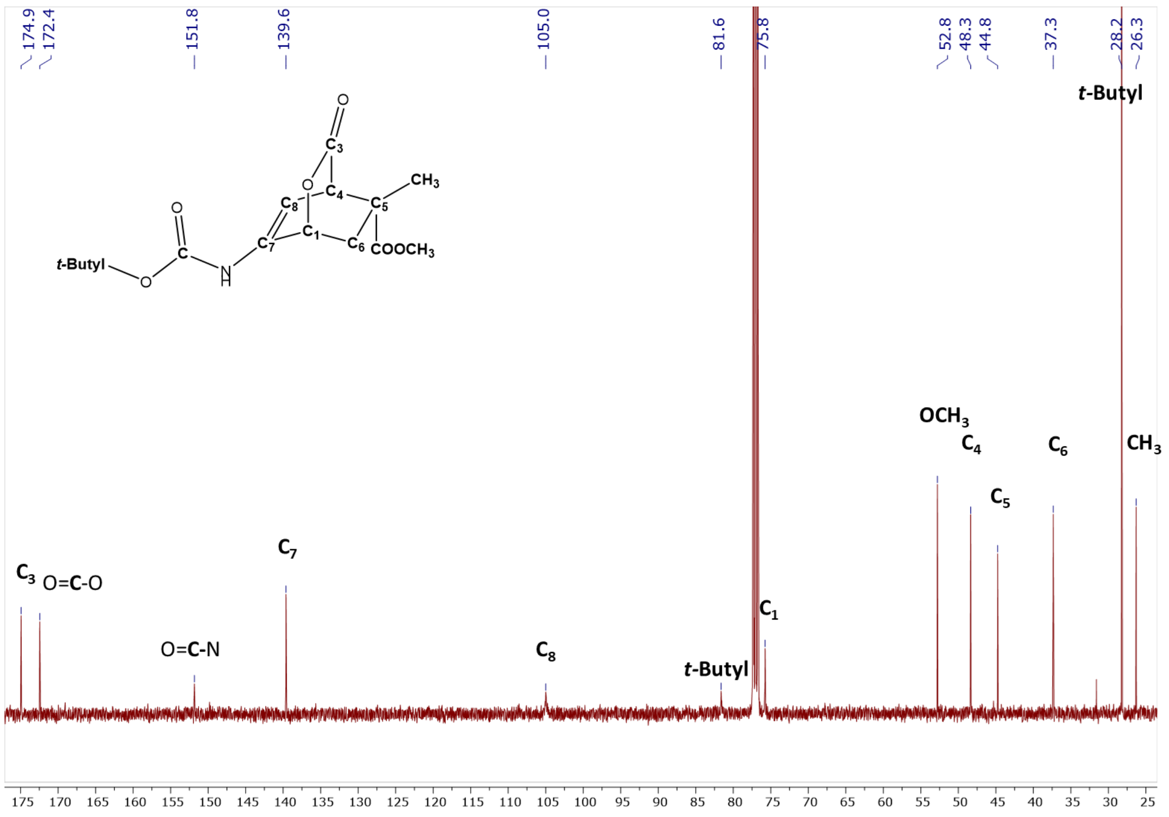

COSY & HSQC of **12a**.

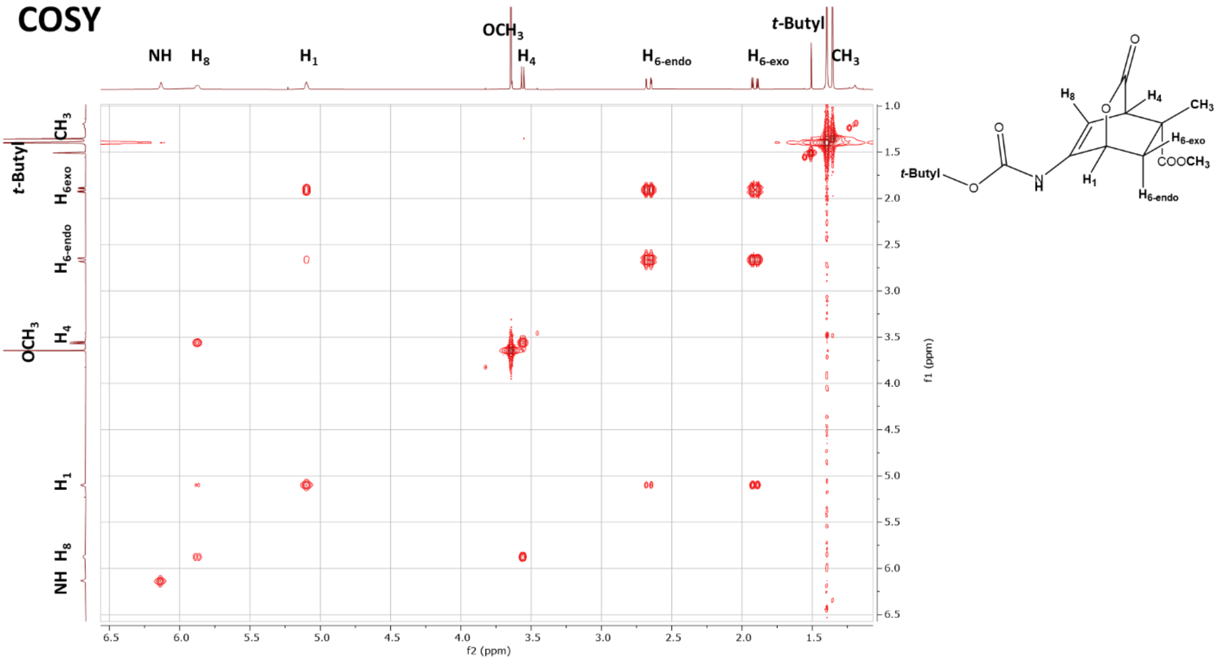

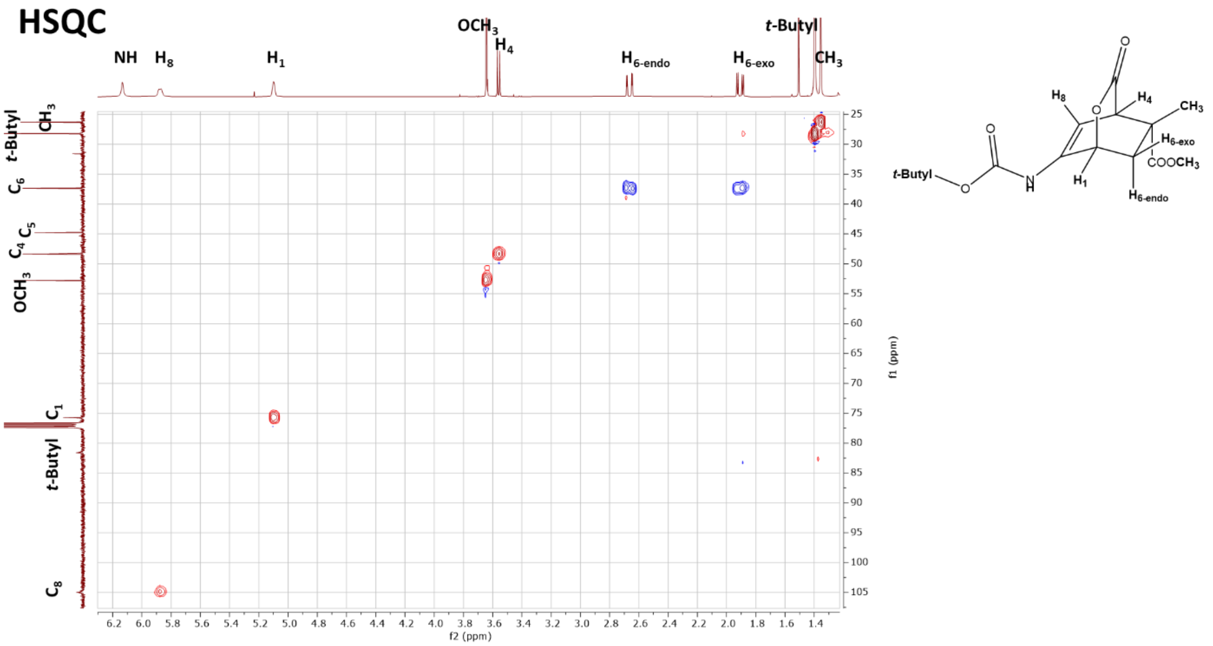

HMBC & NOESY of **12a**.

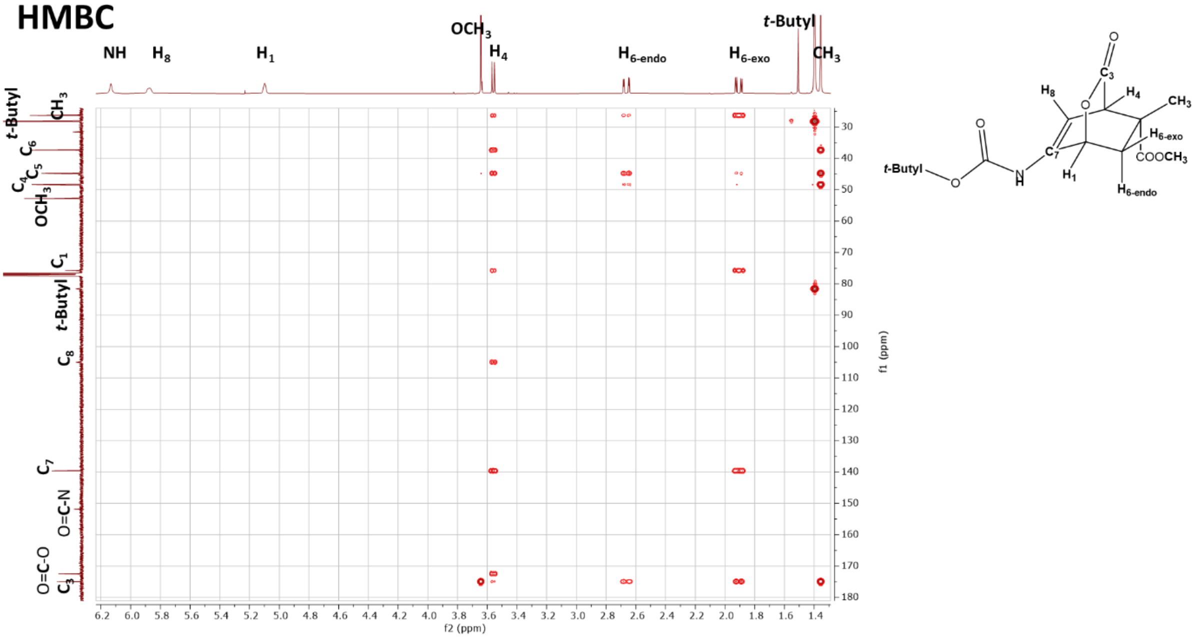

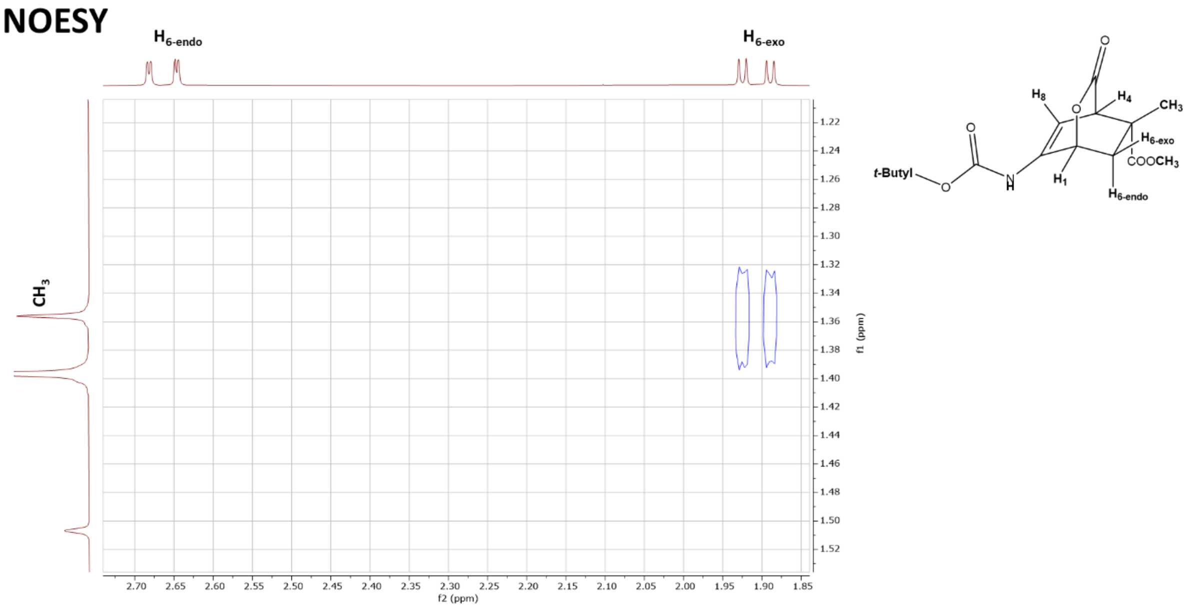

^{1}H & ^{13}C NMR of **12b**.

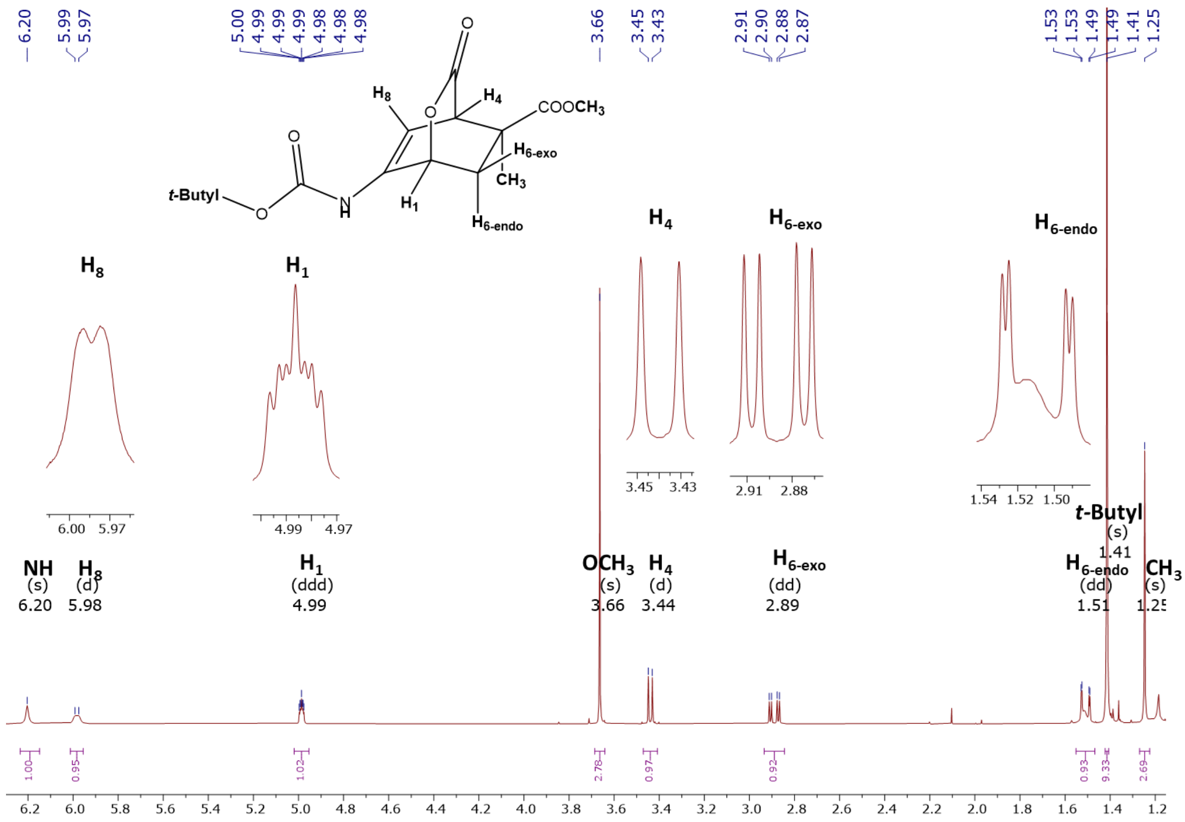

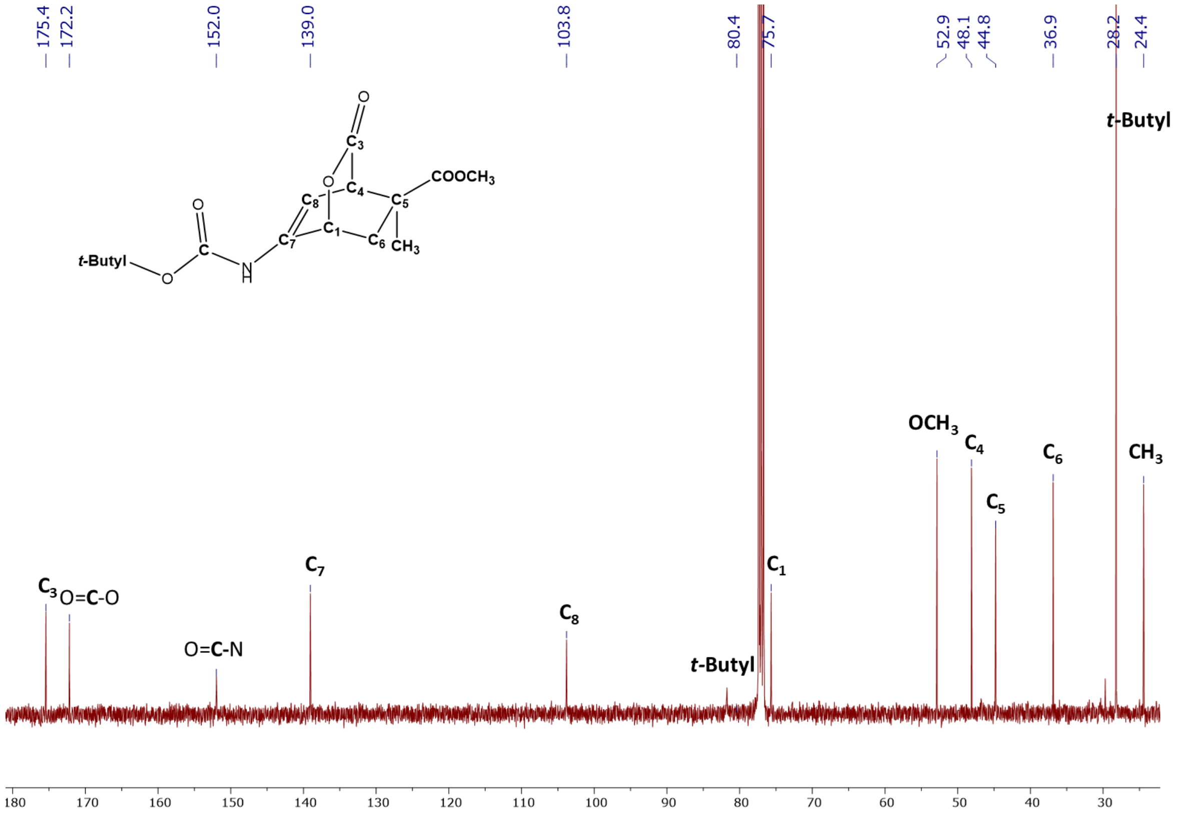

COSY & HSQC of **12b**.

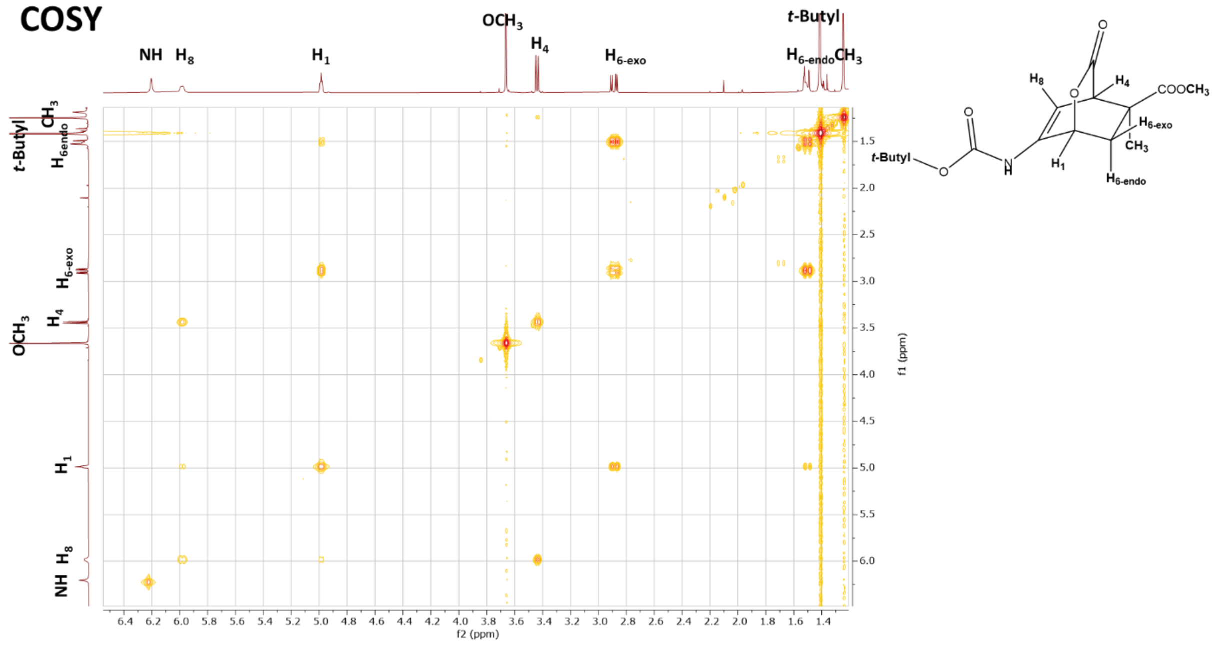

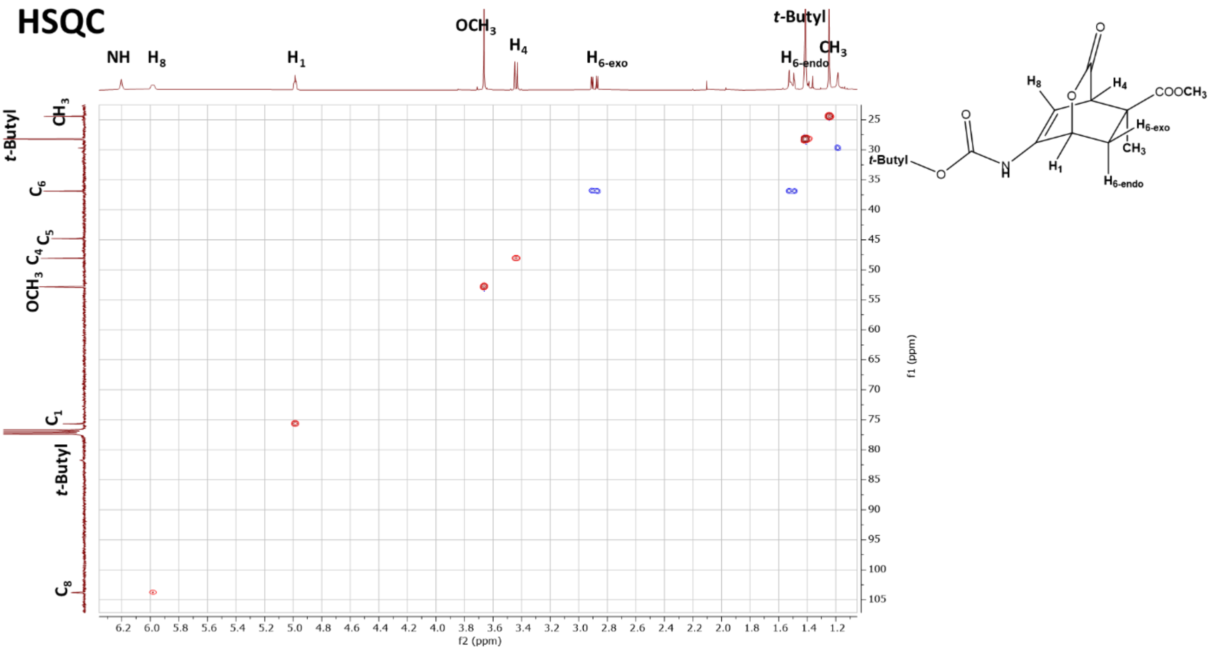

HMBC and NOESY of **12b**.

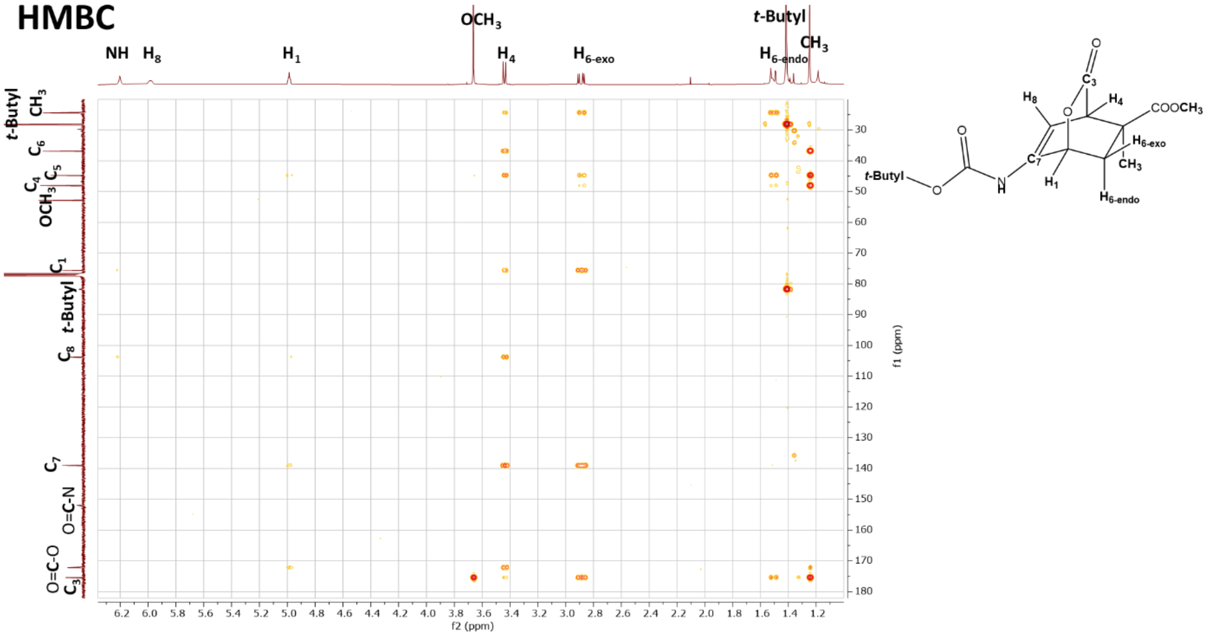

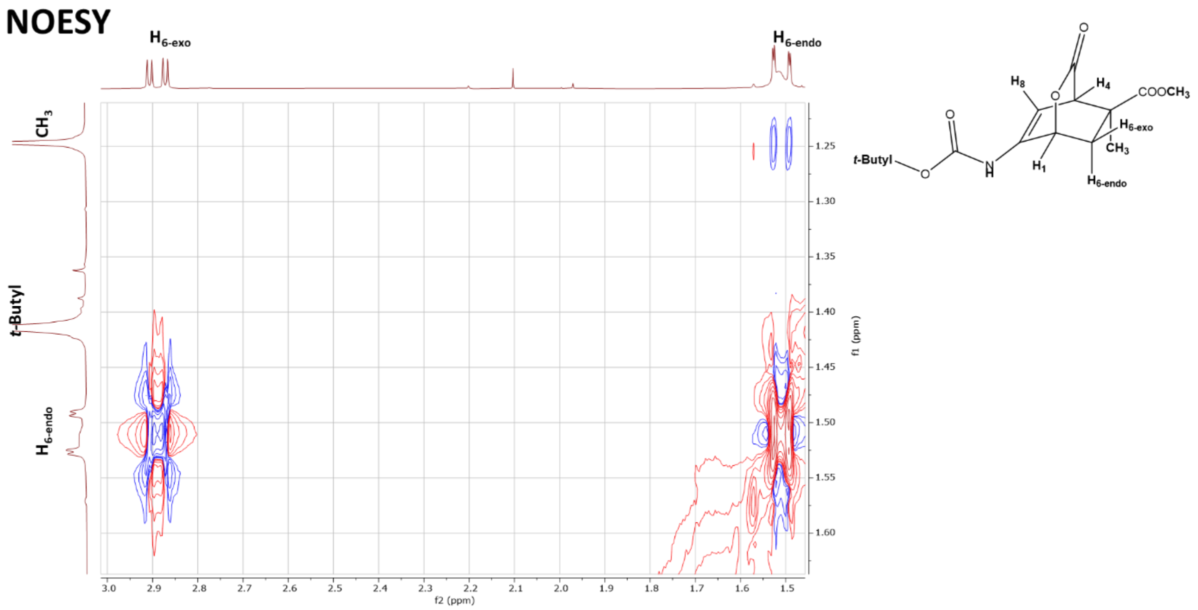

^1H & ^{13}C NMR of **13a**.

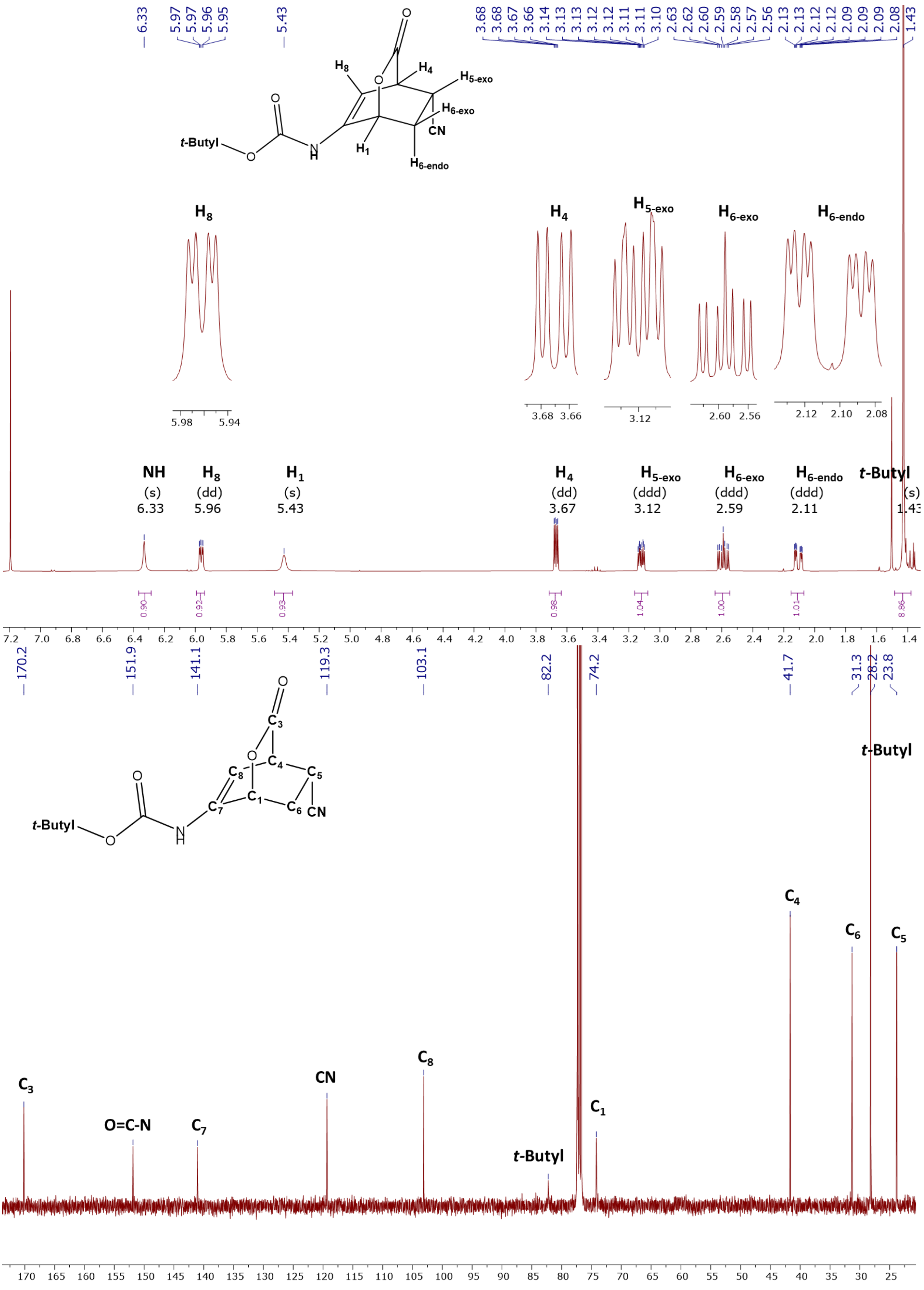

COSY & HSQC of **13a**.

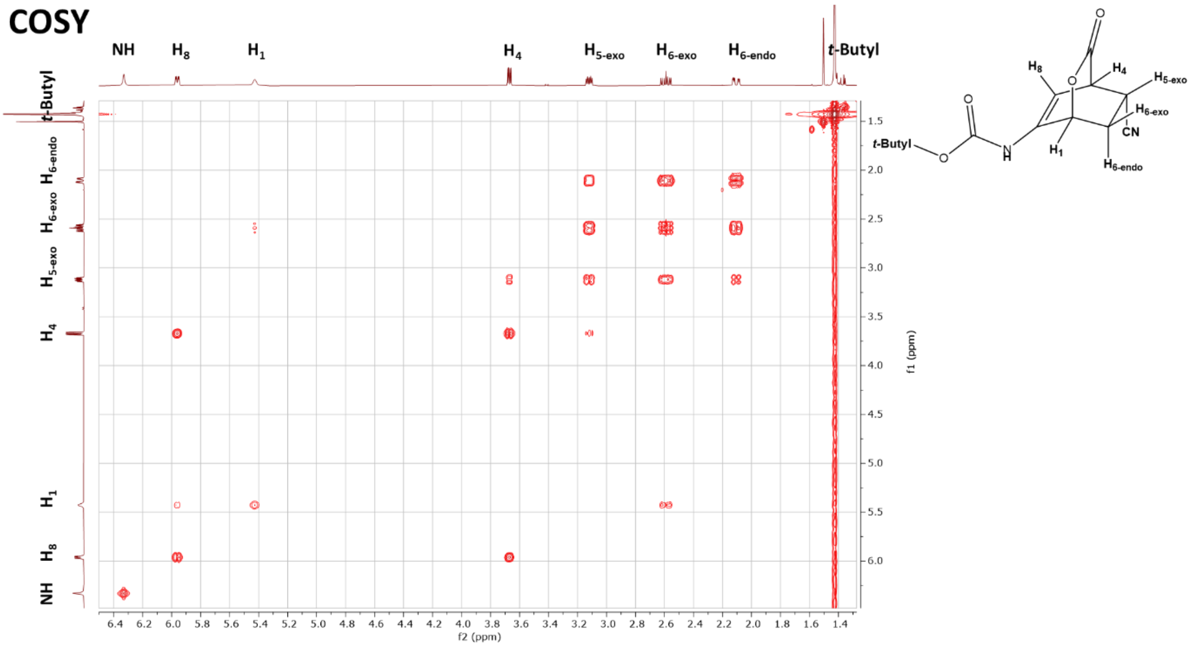

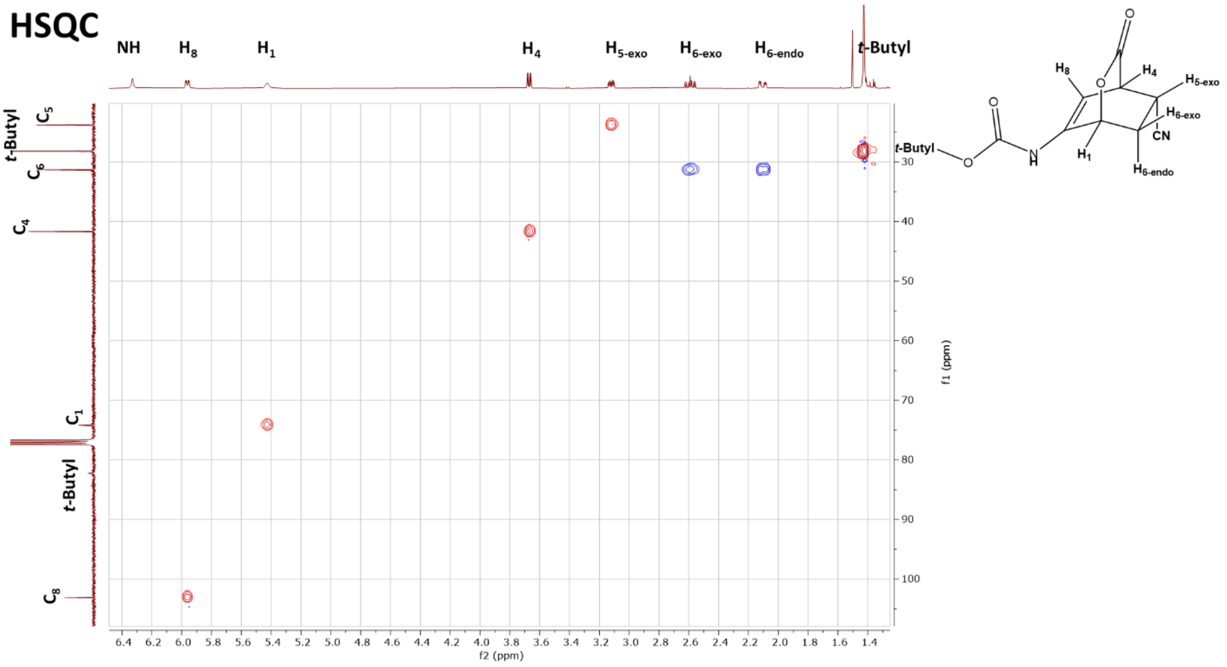

^{1}H & ^{13}C NMR of **13b.**

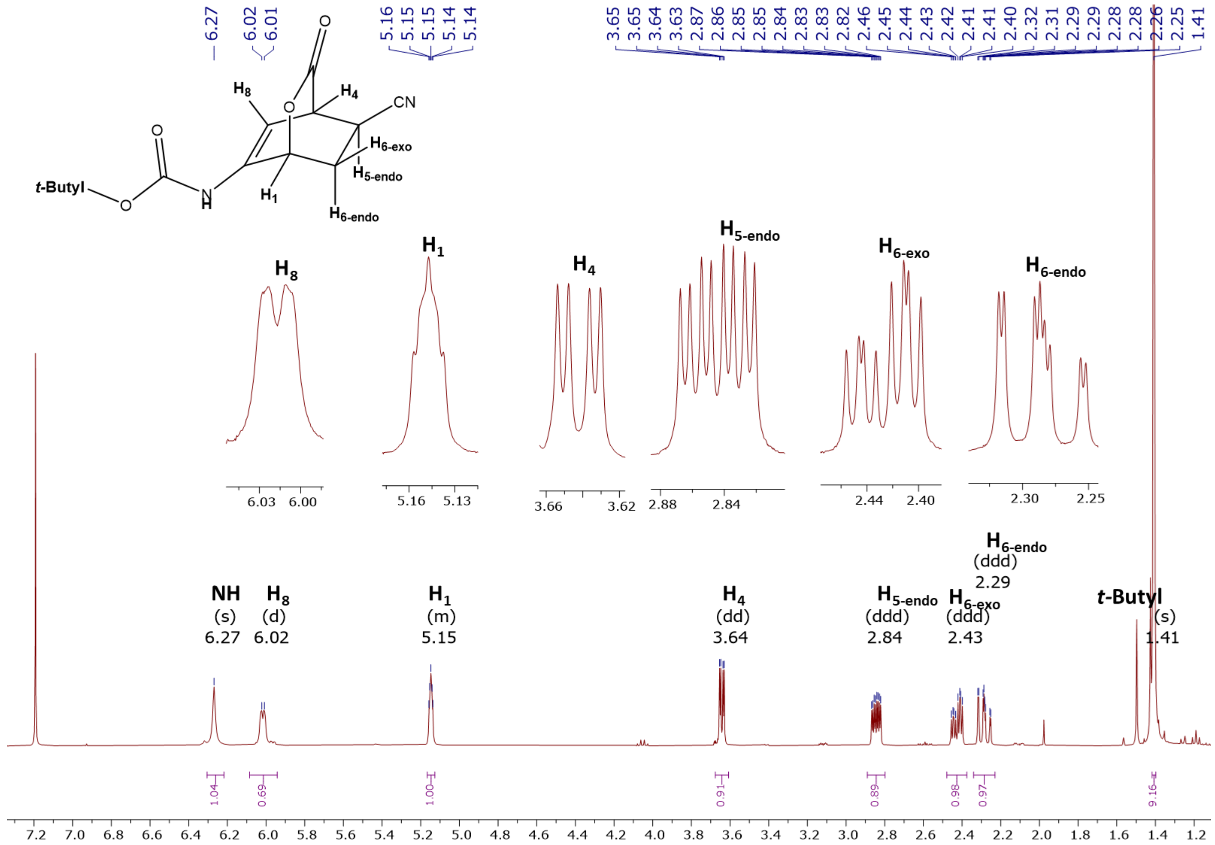

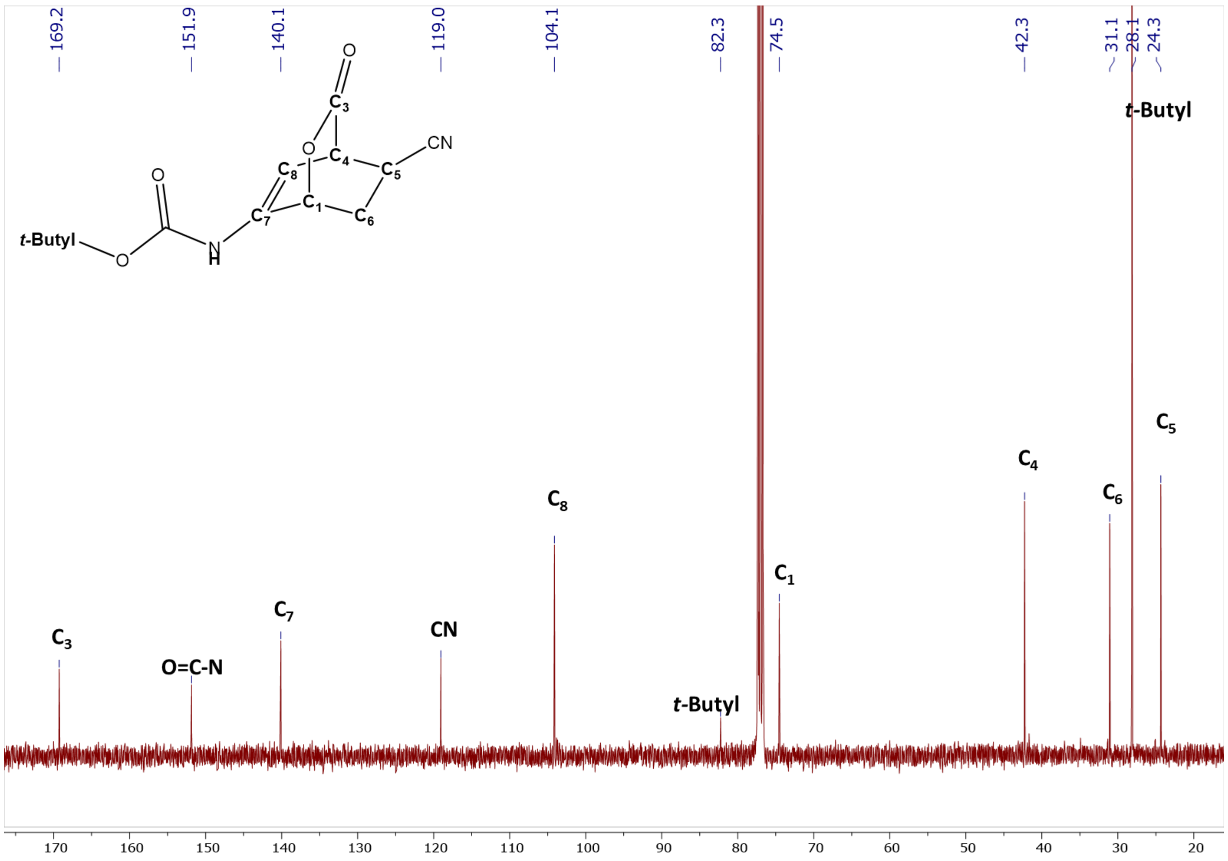

COSY & HSQC of **13b**.

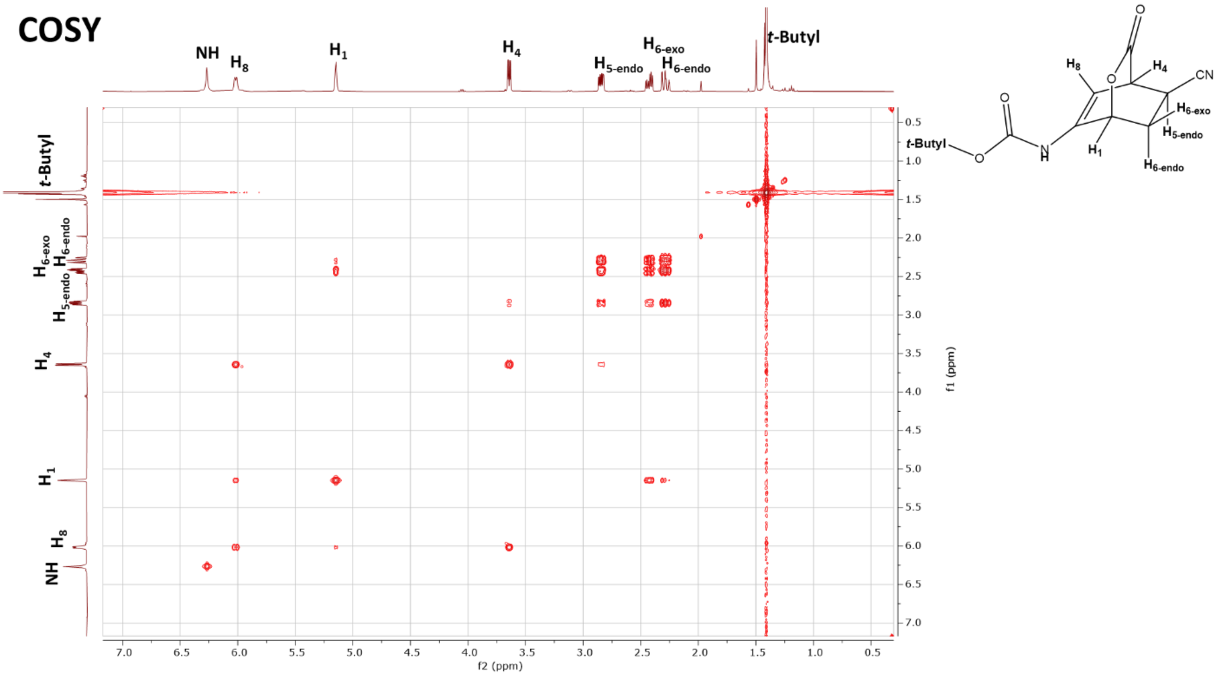

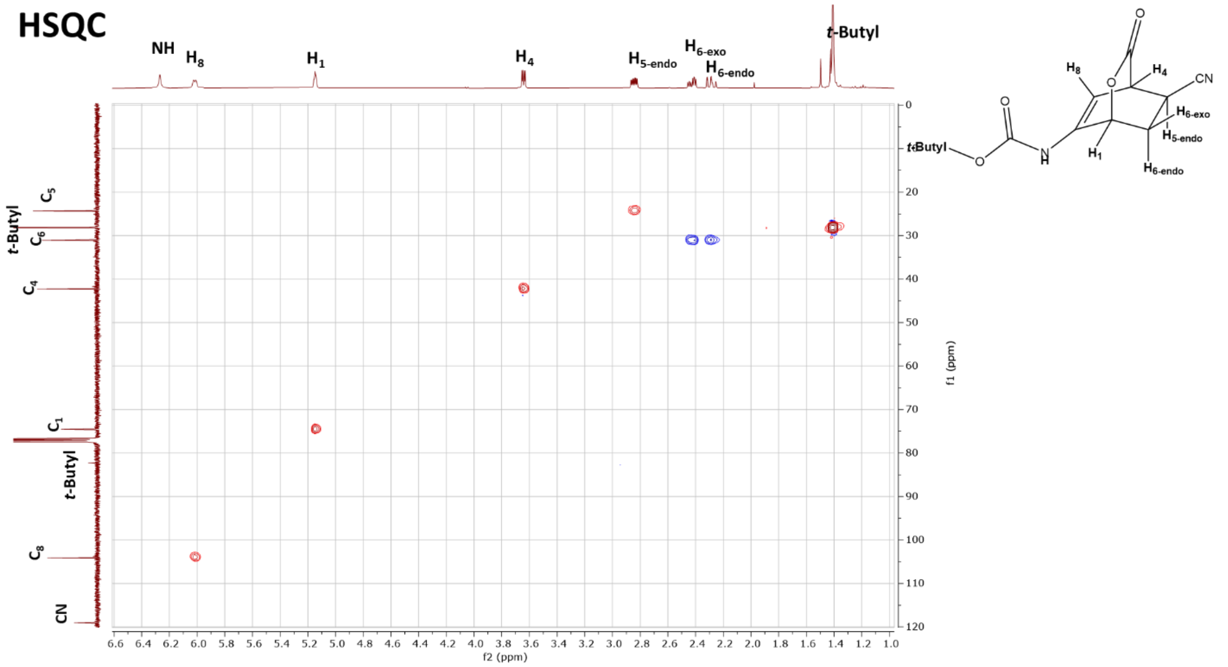

^{1}H & ^{13}C NMR of **14a**.

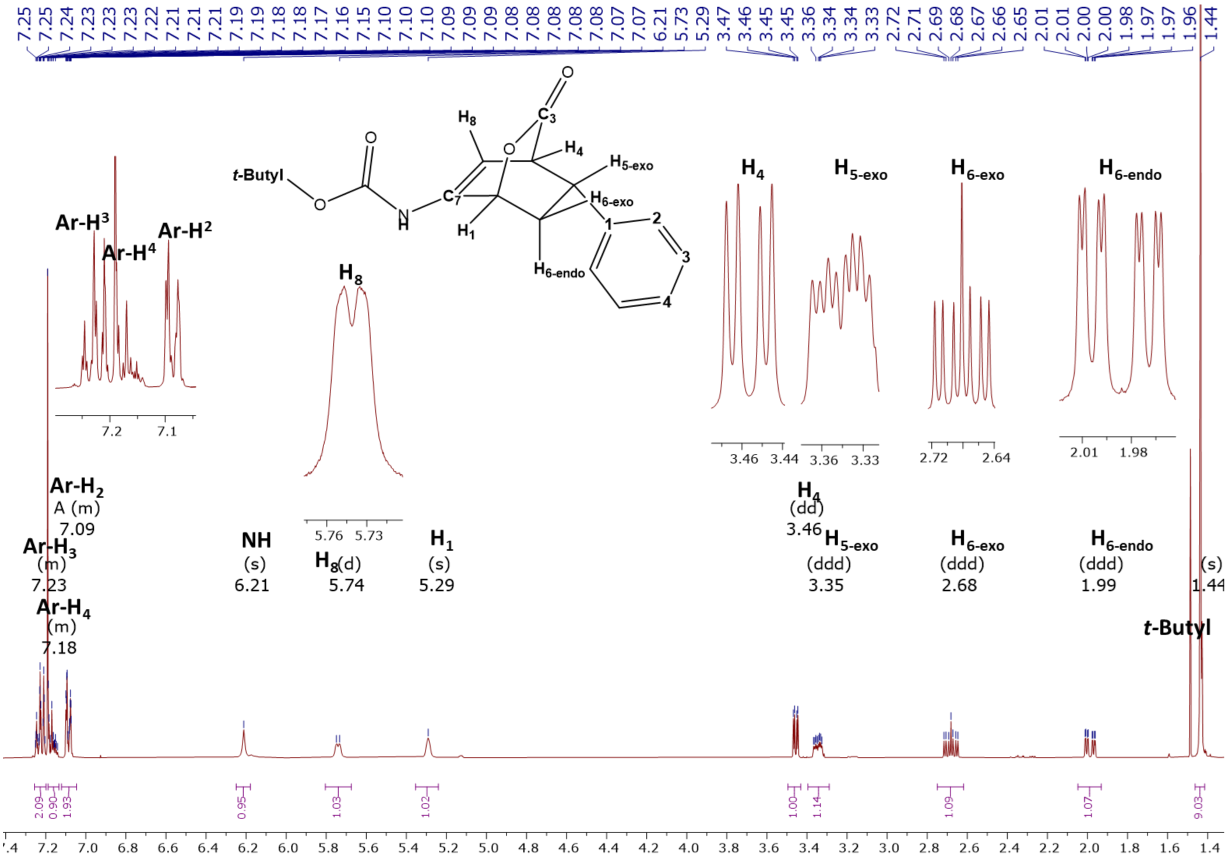

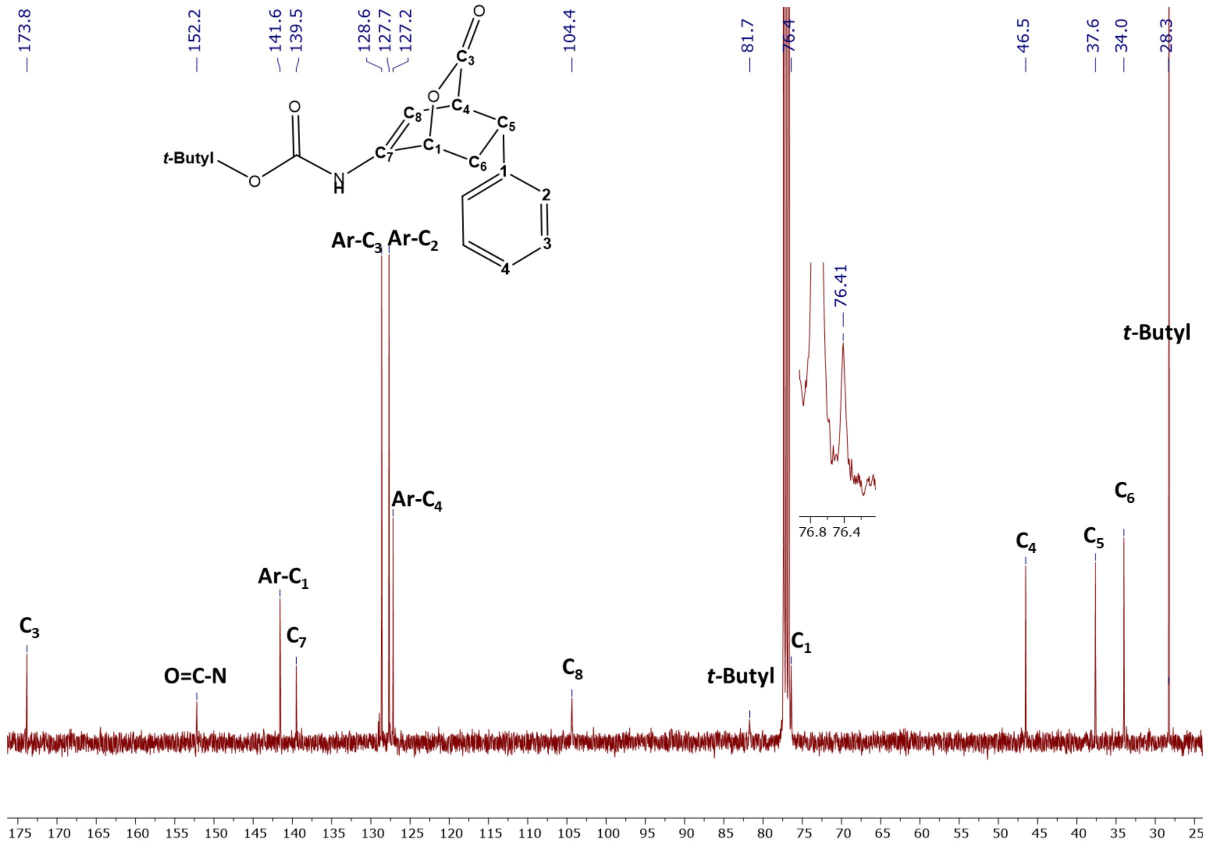

COSY & HSQC of **14a**.

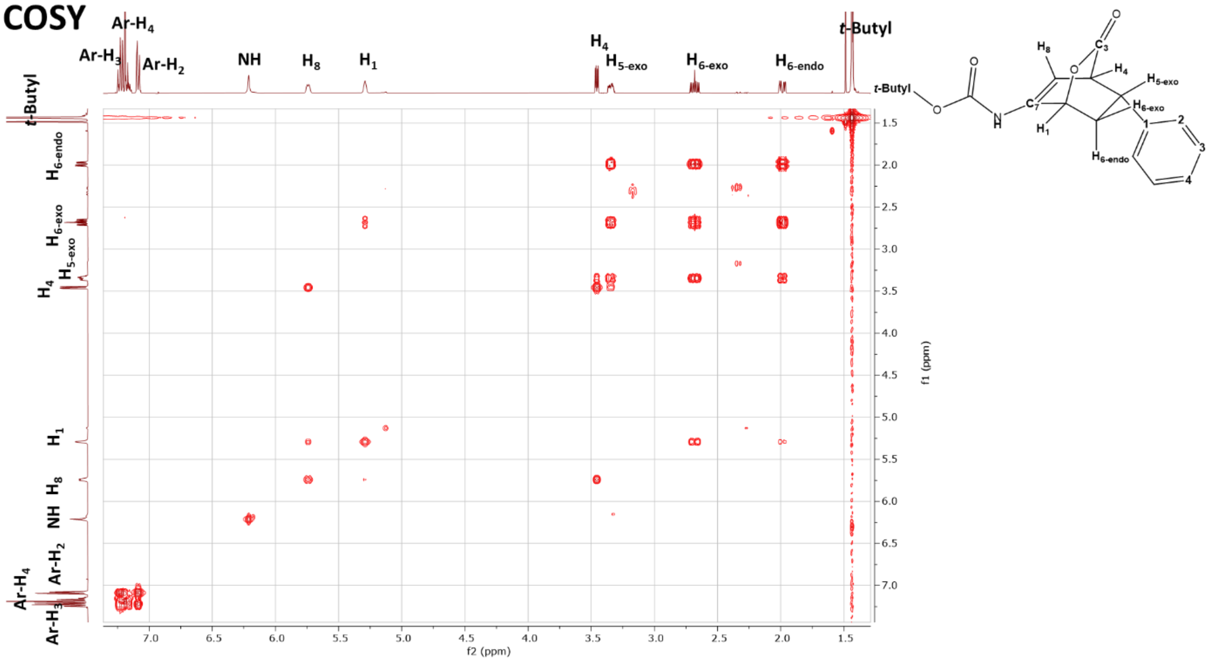

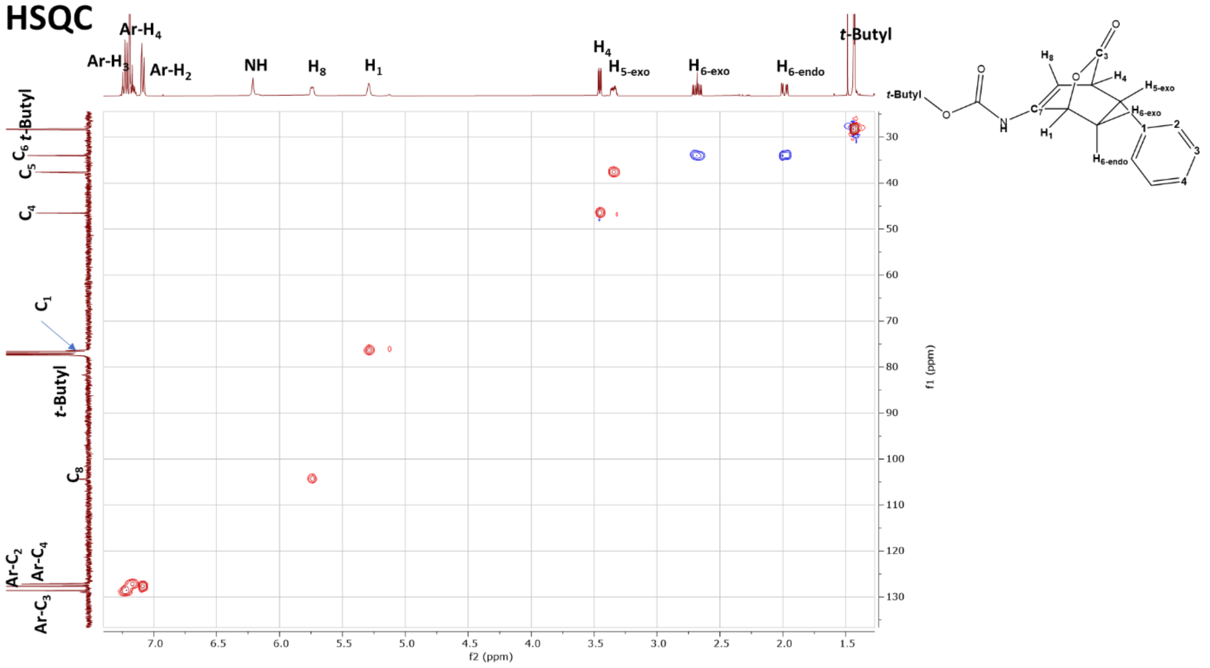

[1]H & of **14b** as a mixture with **14a**

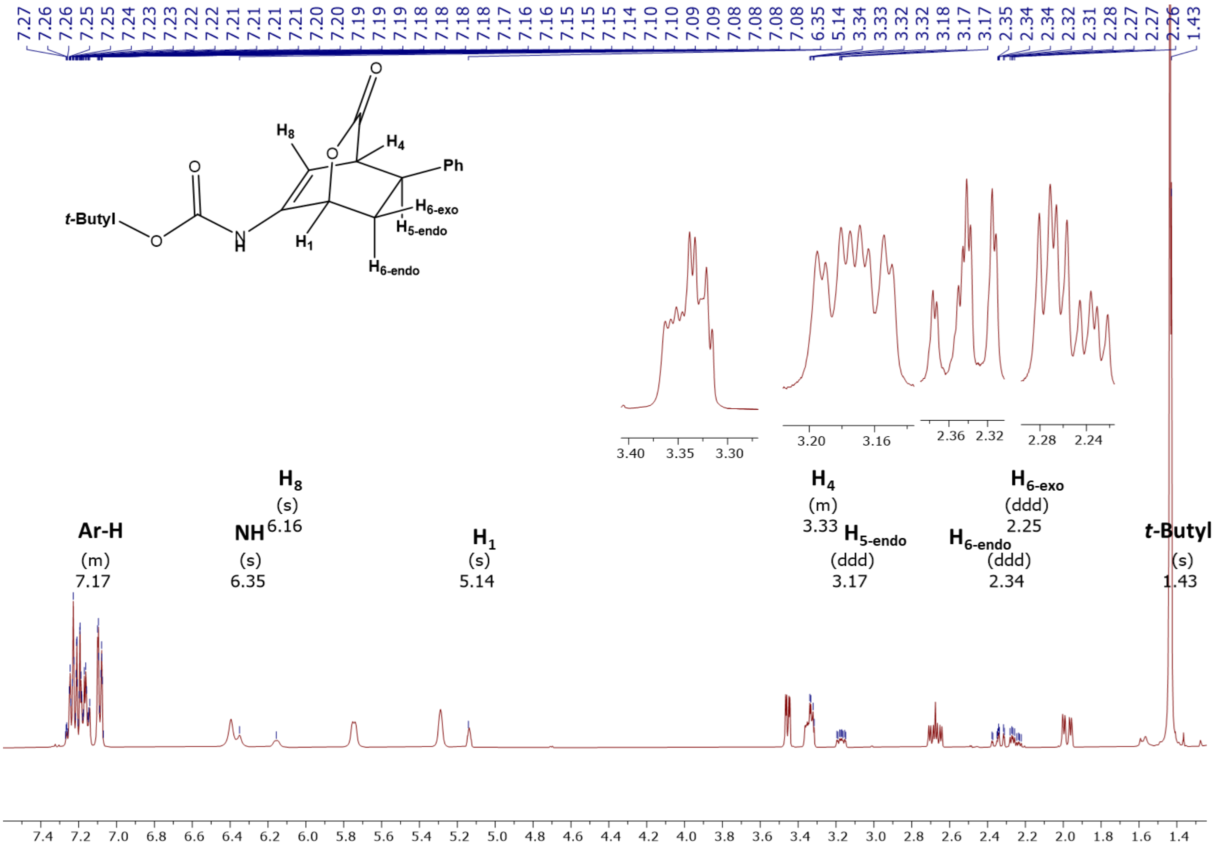

[1]H of **15a** as a mixture with **15b**

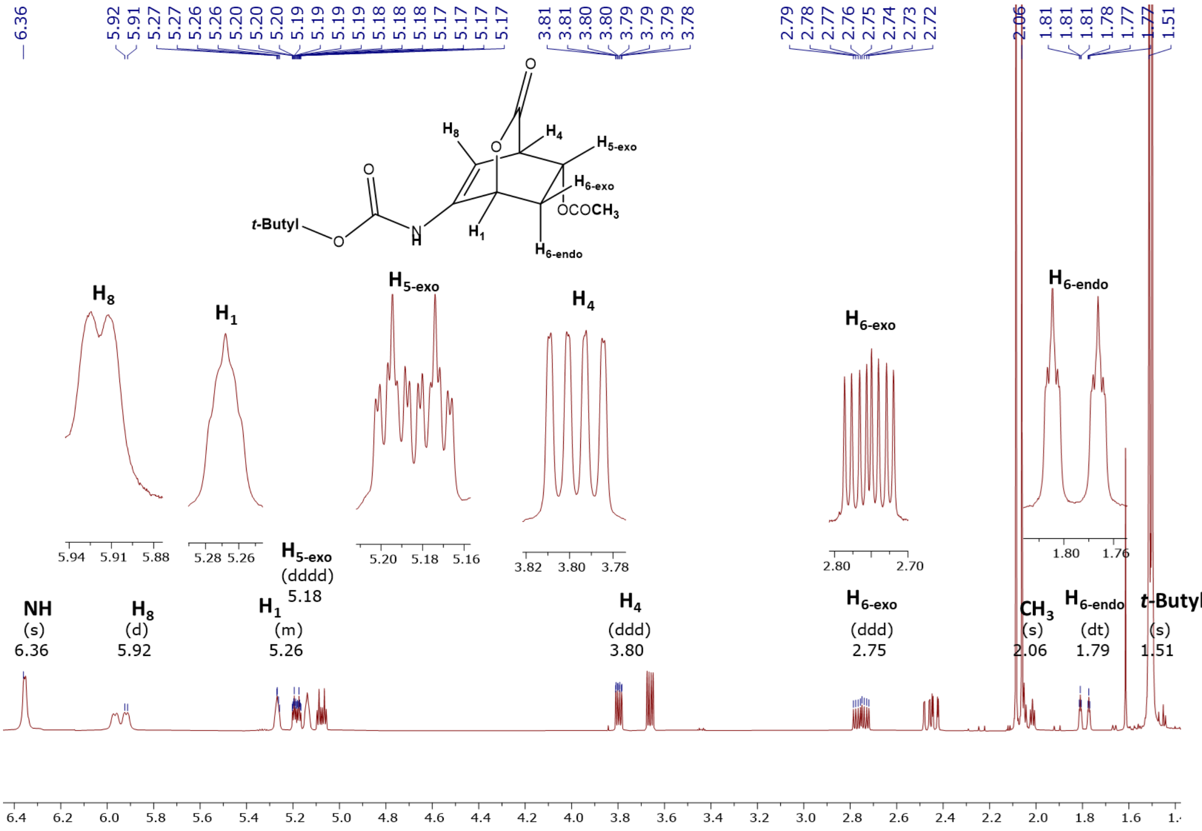

^{1}H & ^{13}C NMR of **15b**.

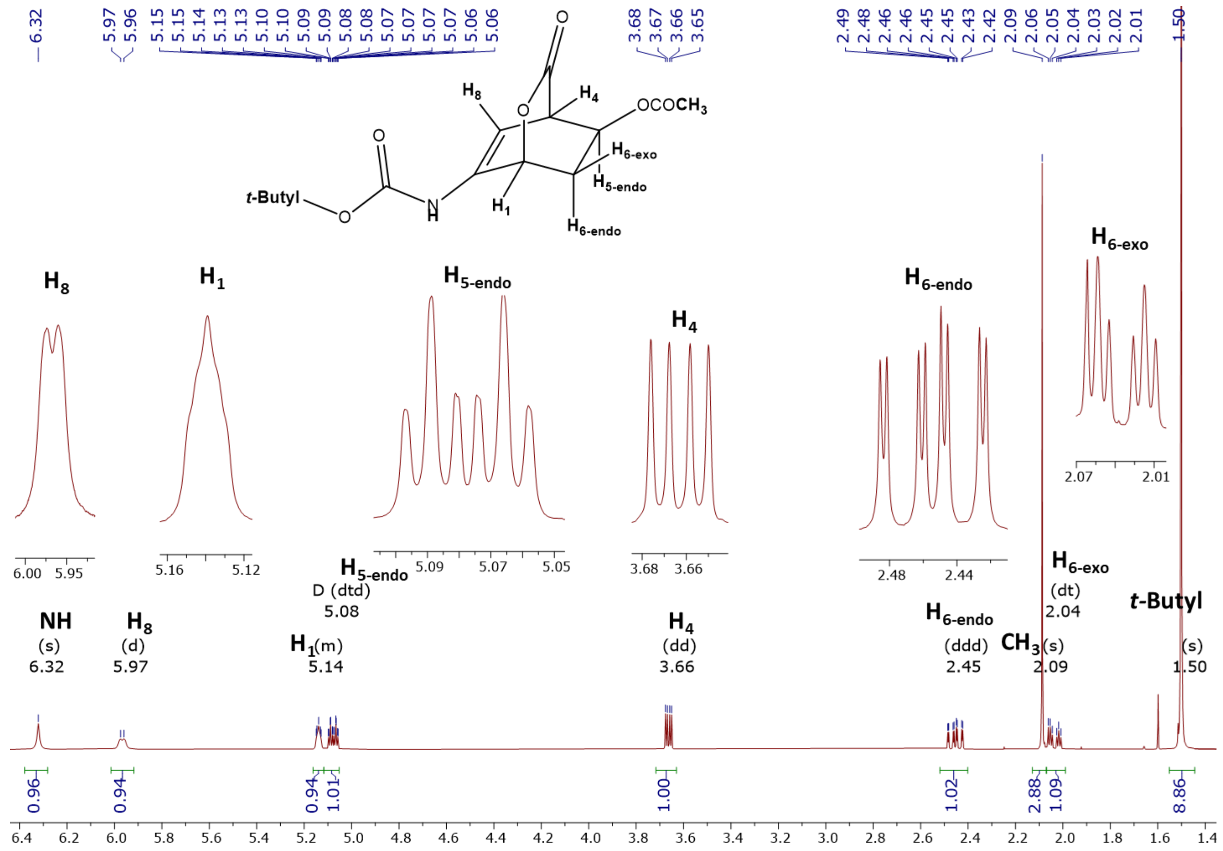

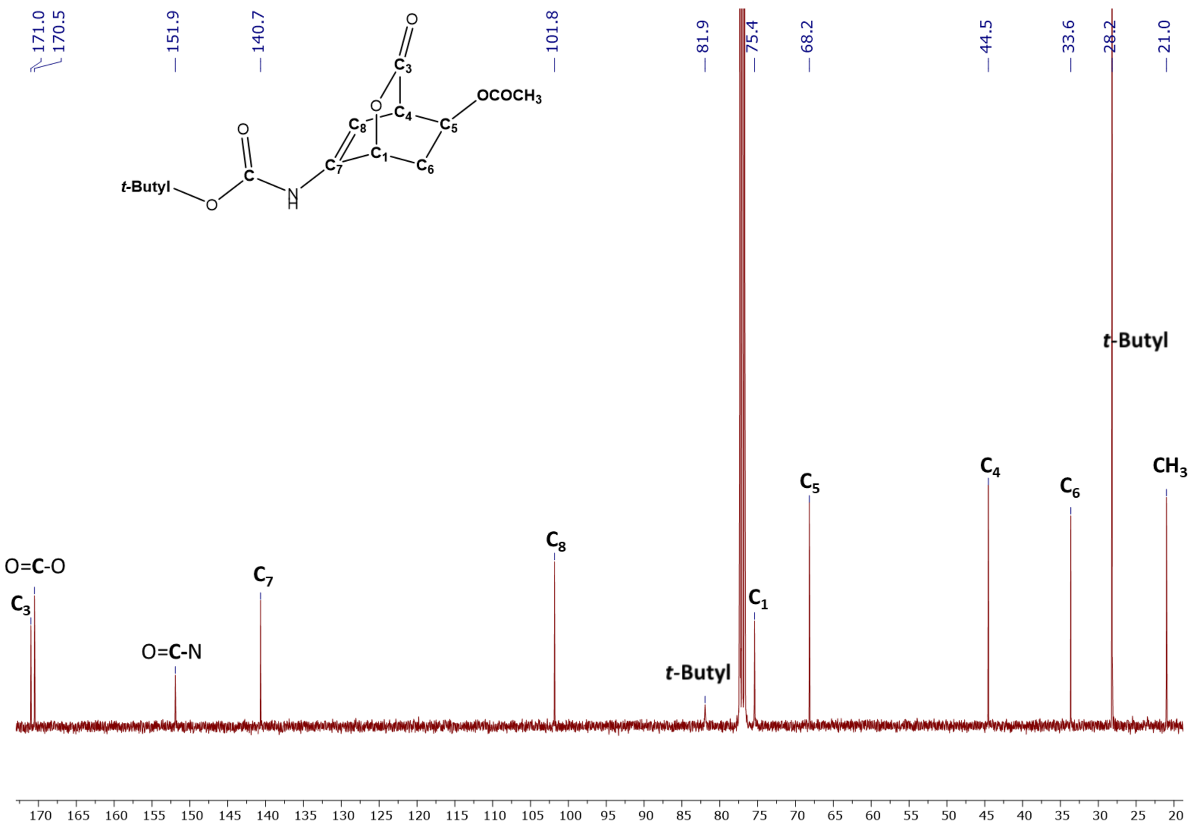

COSY and HSQC of **15b.**

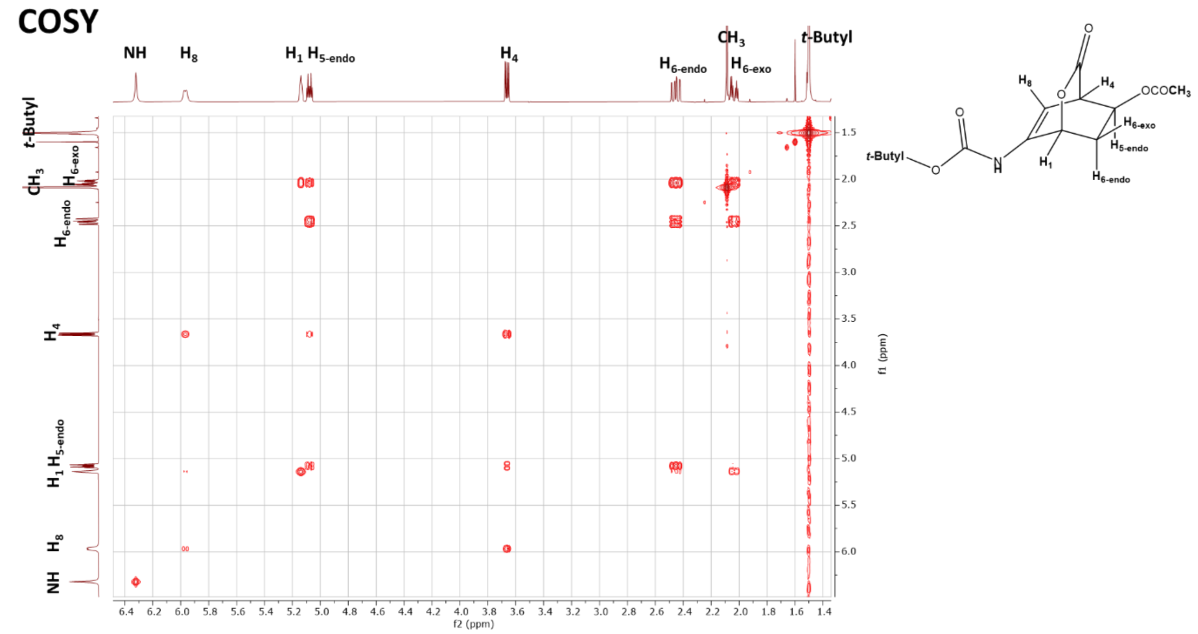

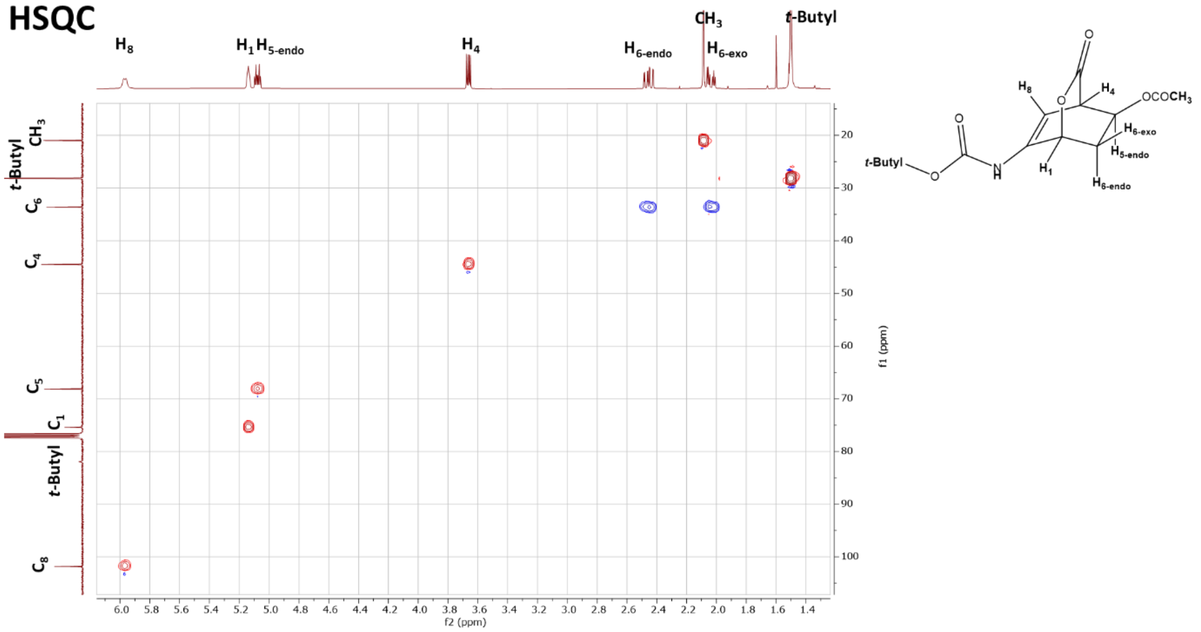

^{1}H & ^{13}C NMR of **15c**.

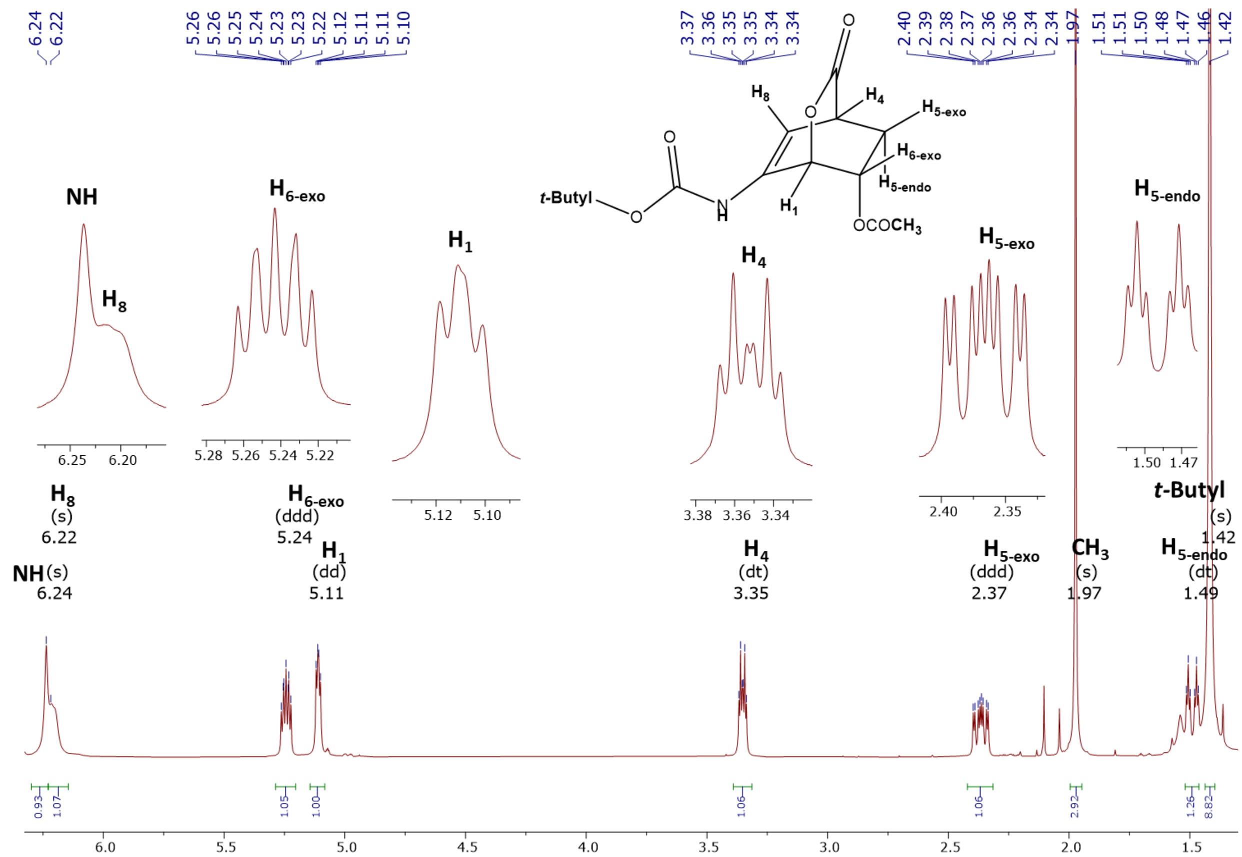

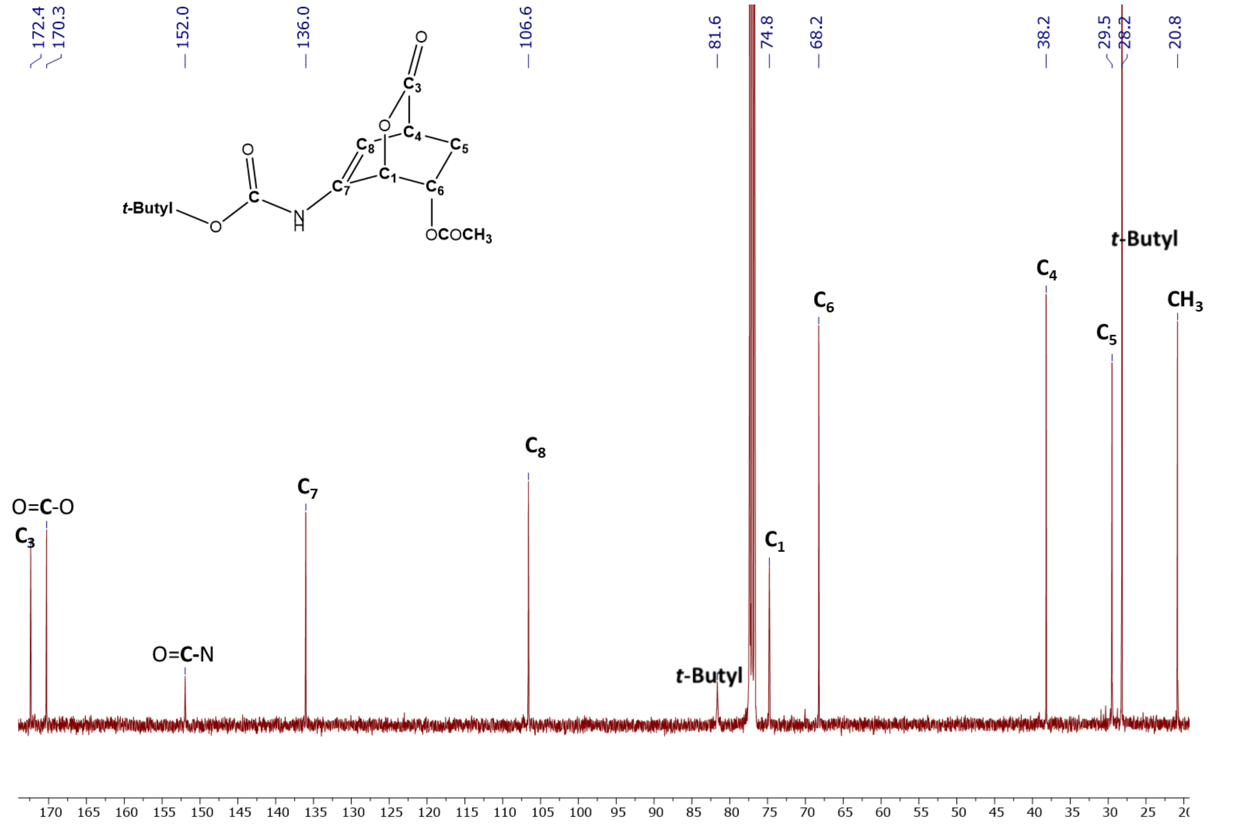

COSY & HSQC of **15c**.

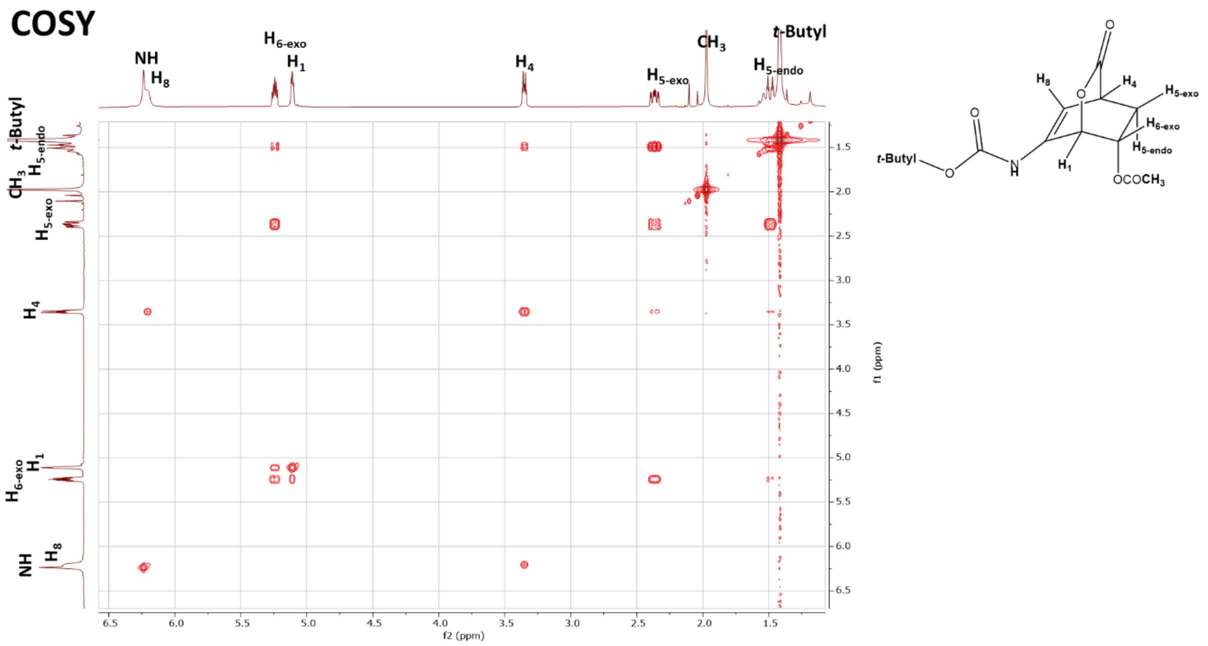

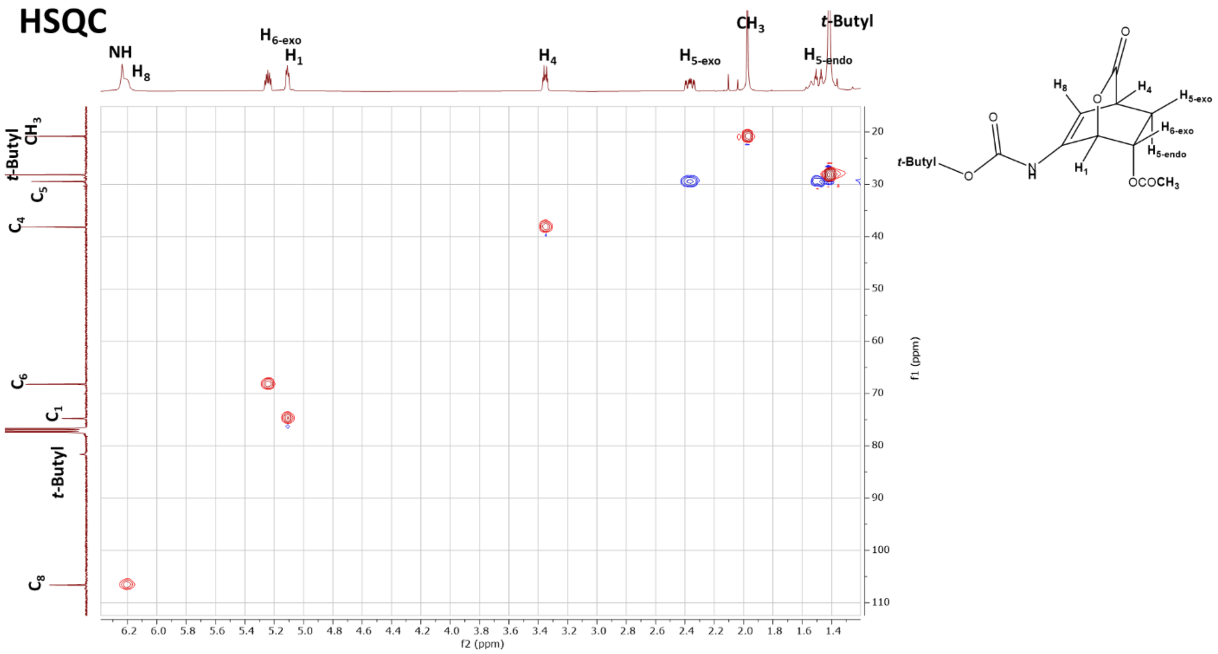

^{1}H & ^{13}C NMR for dimer **16a**

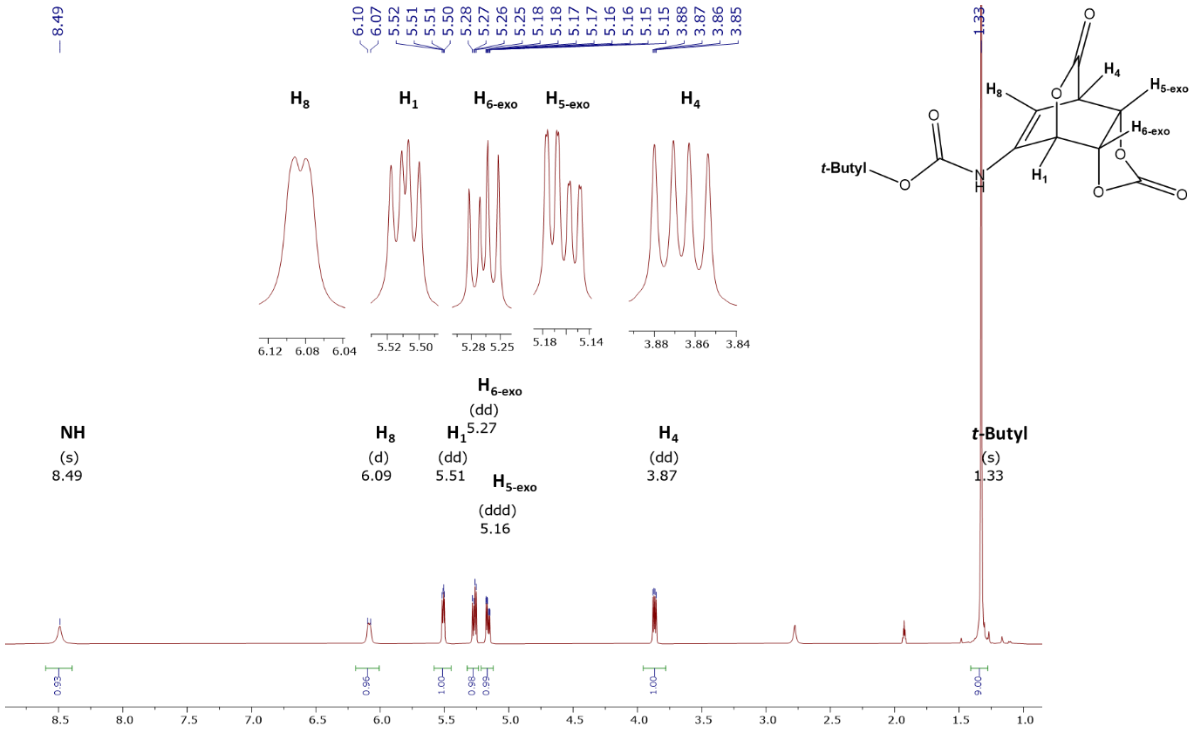

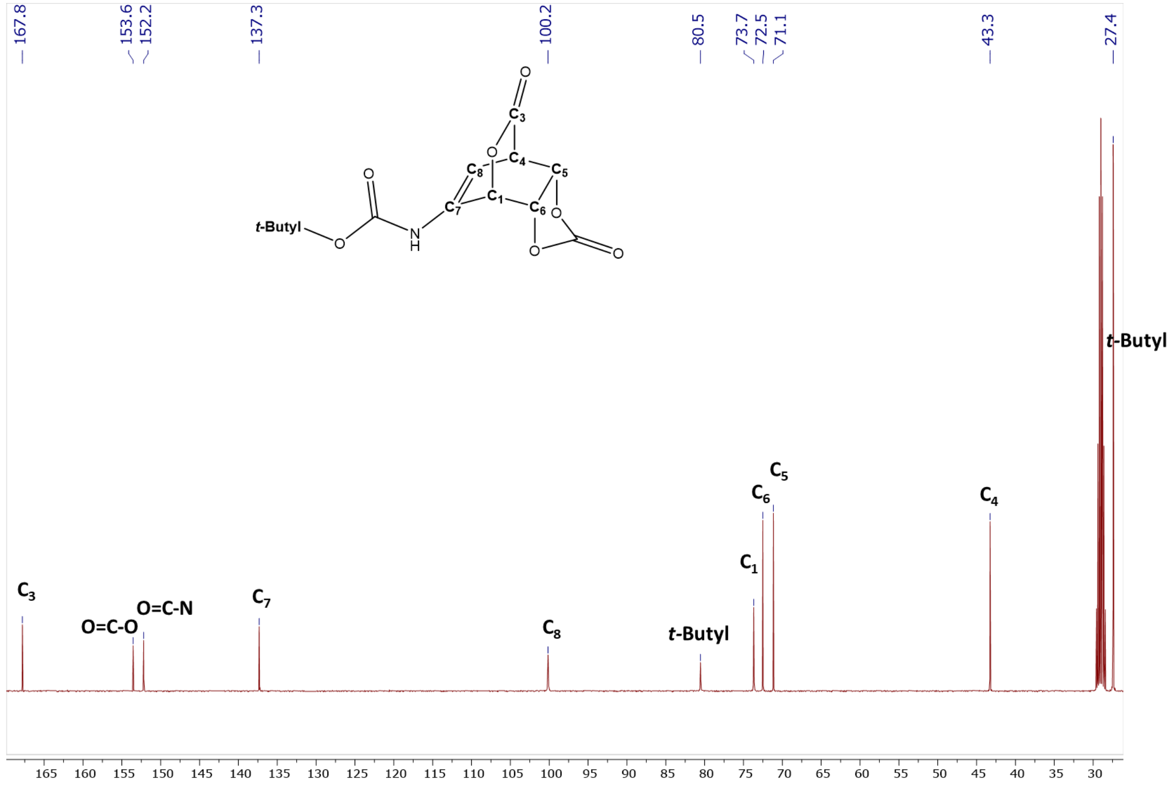

COSY & HSQC of **16a**

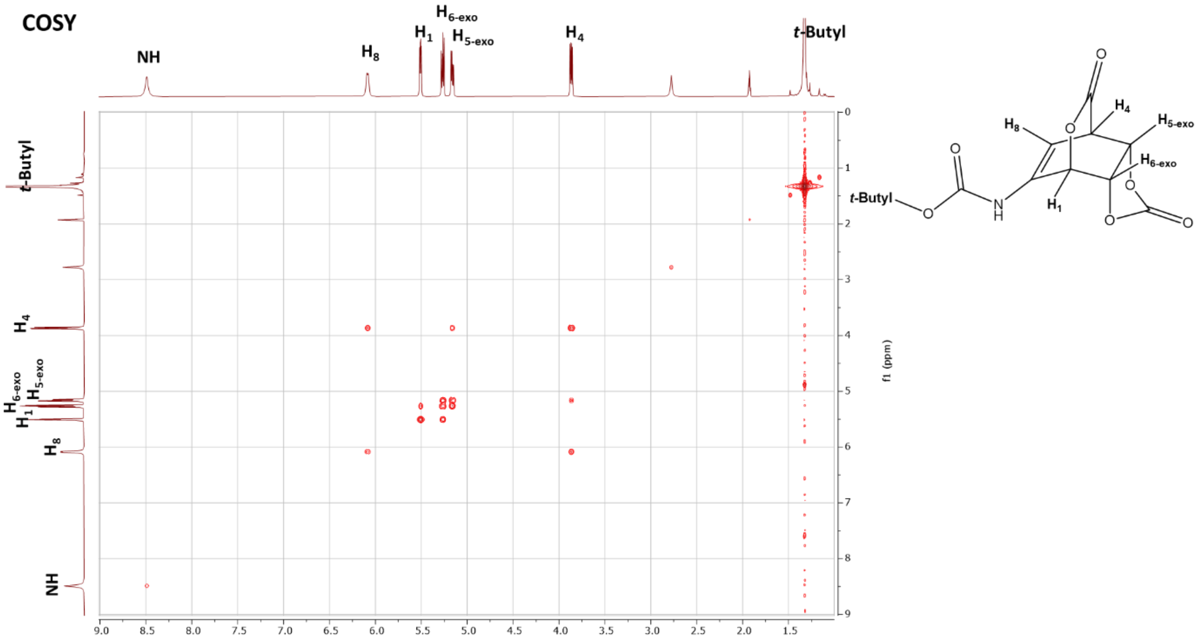

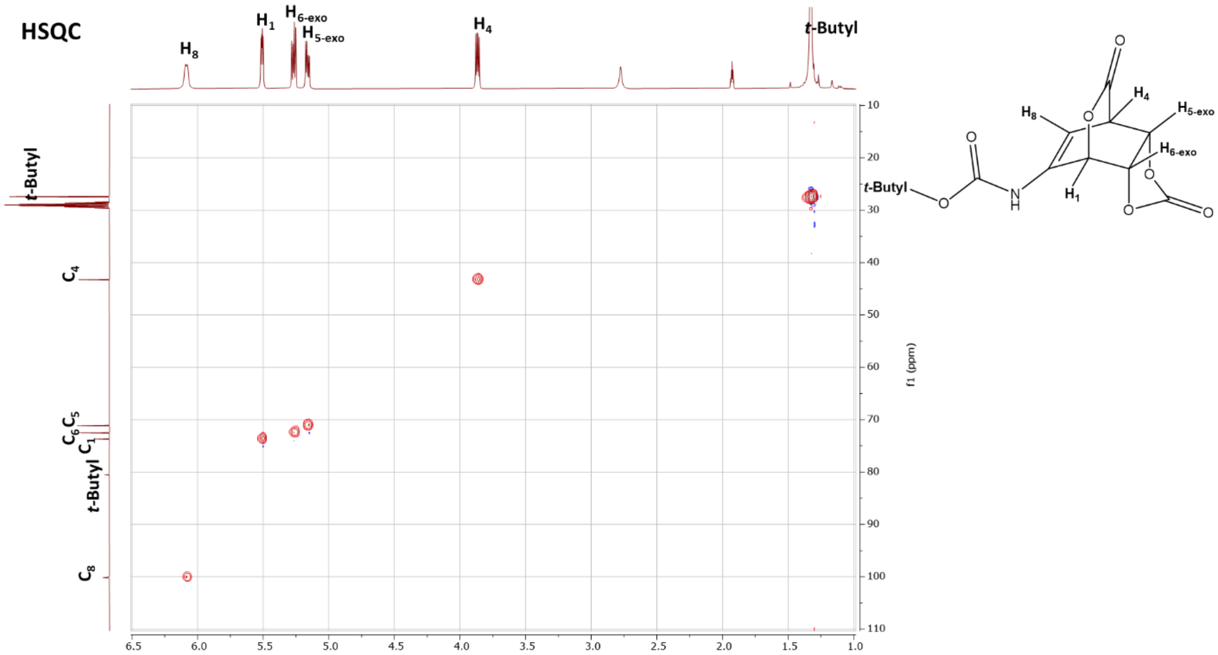

^{1}H & ^{13}C NMR for dimer **16b**

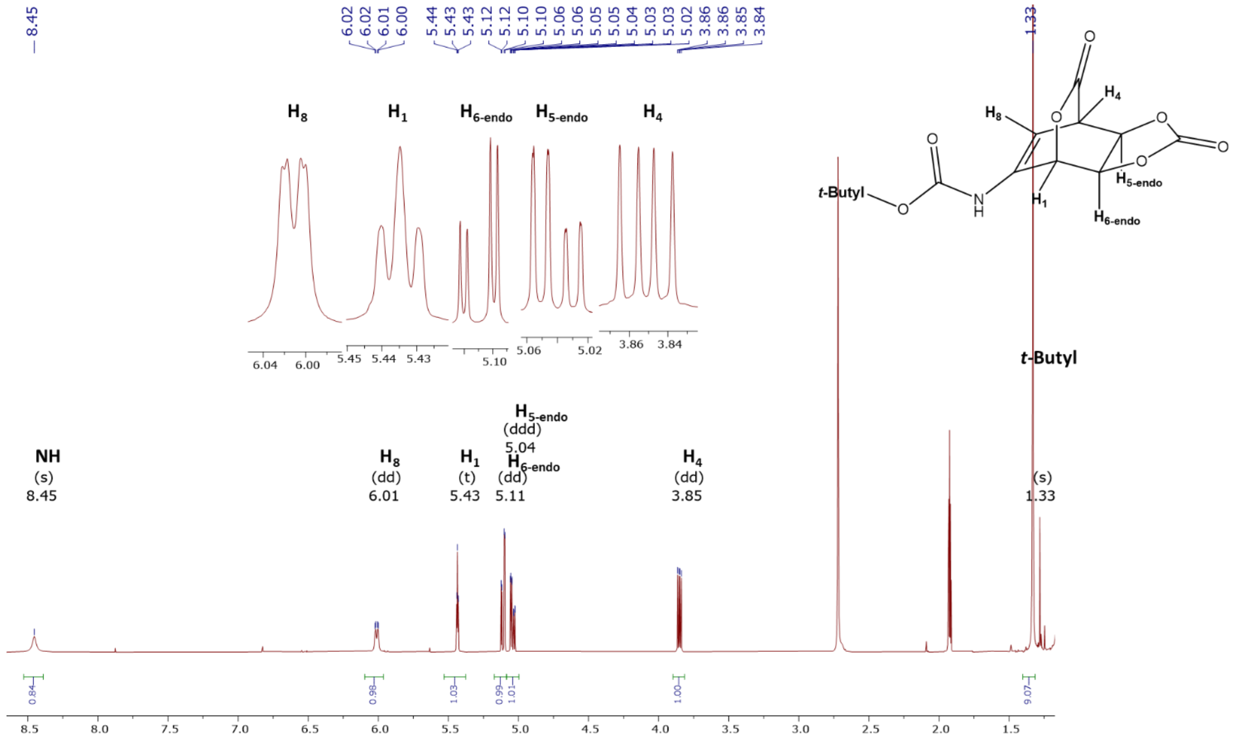

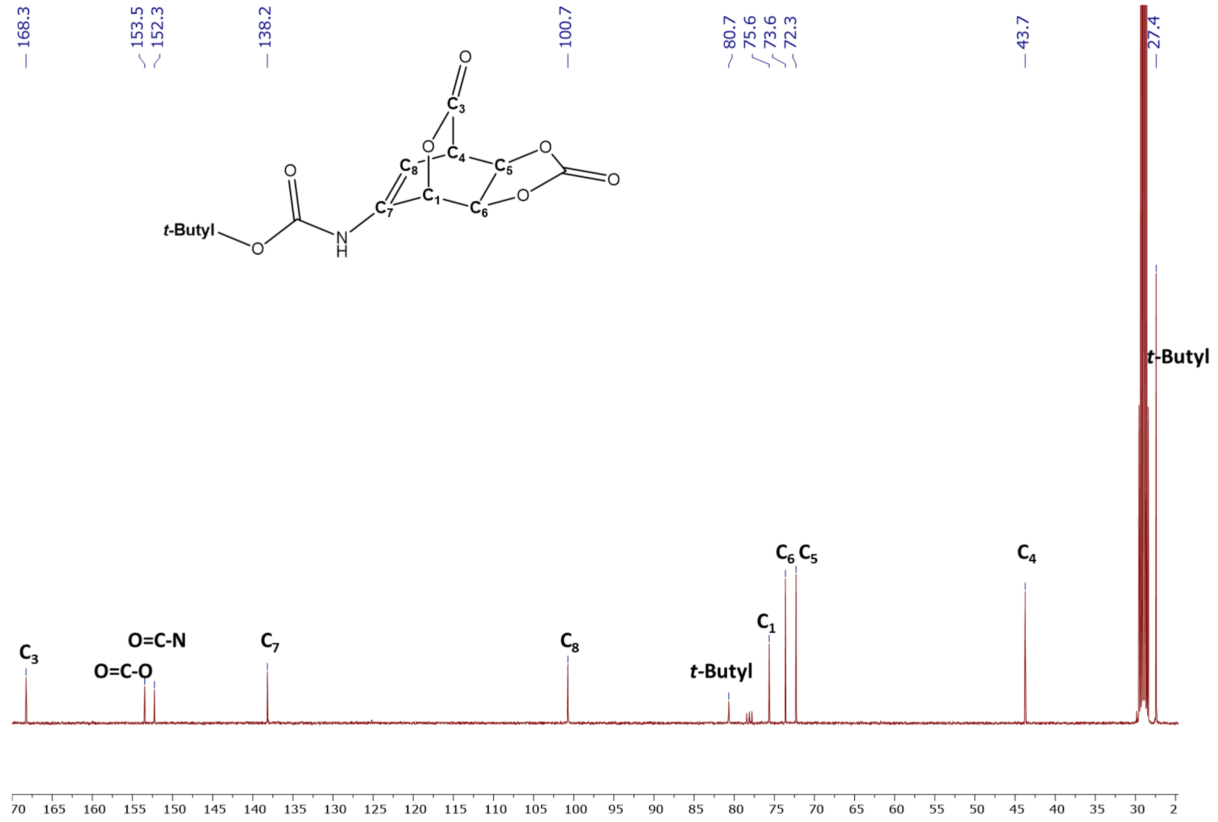

COSY & HSQC of **16b**

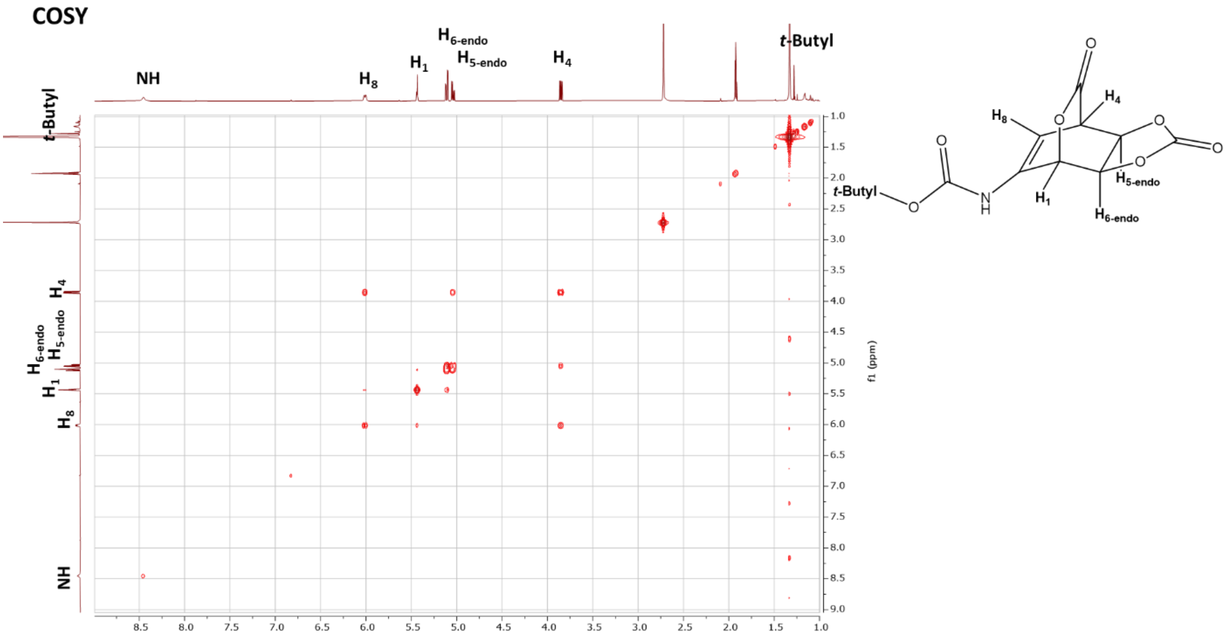

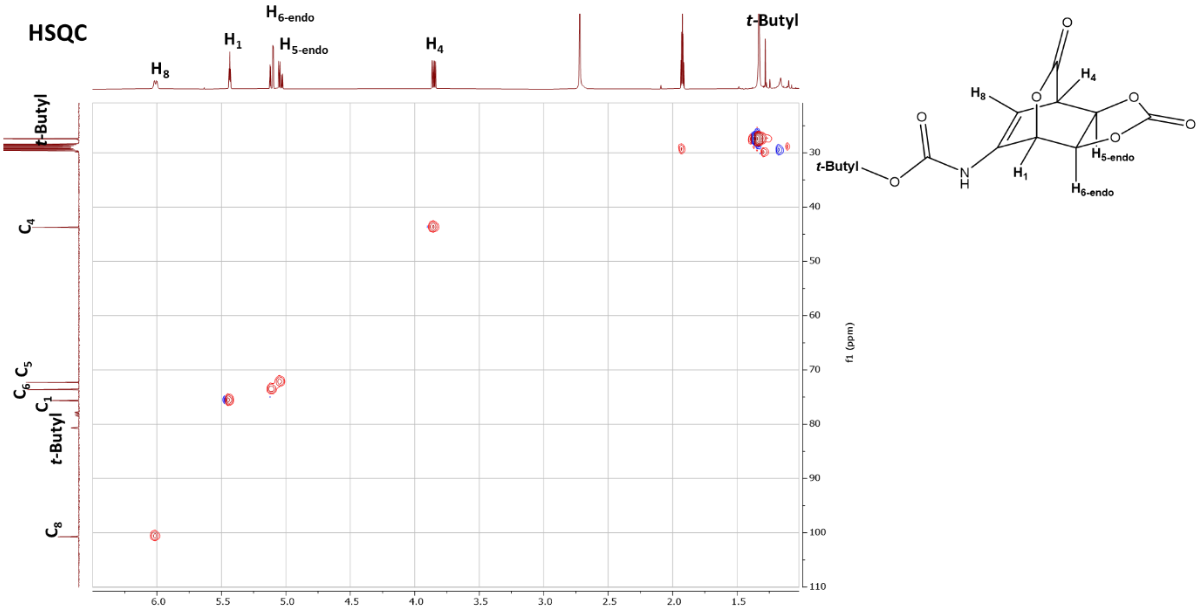

^{1}H & ^{13}C NMR for dimer **17**

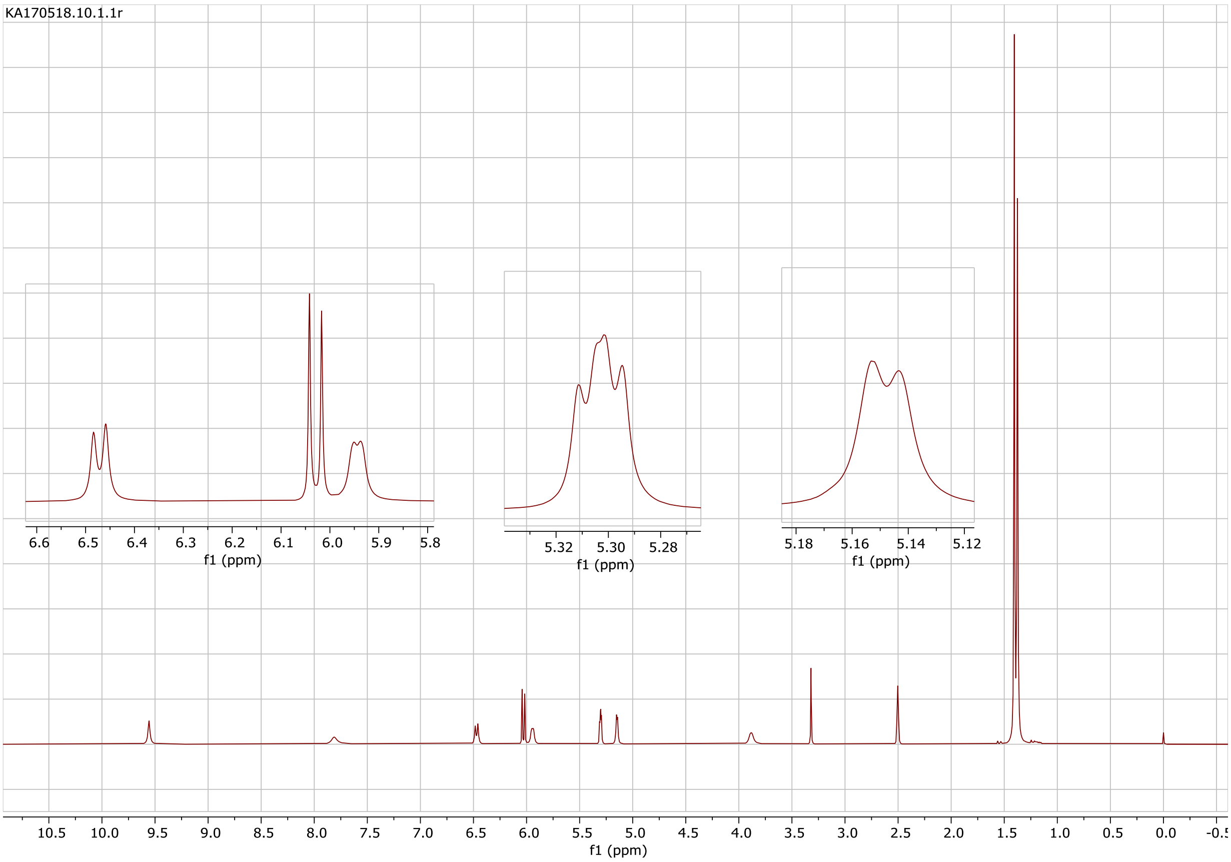

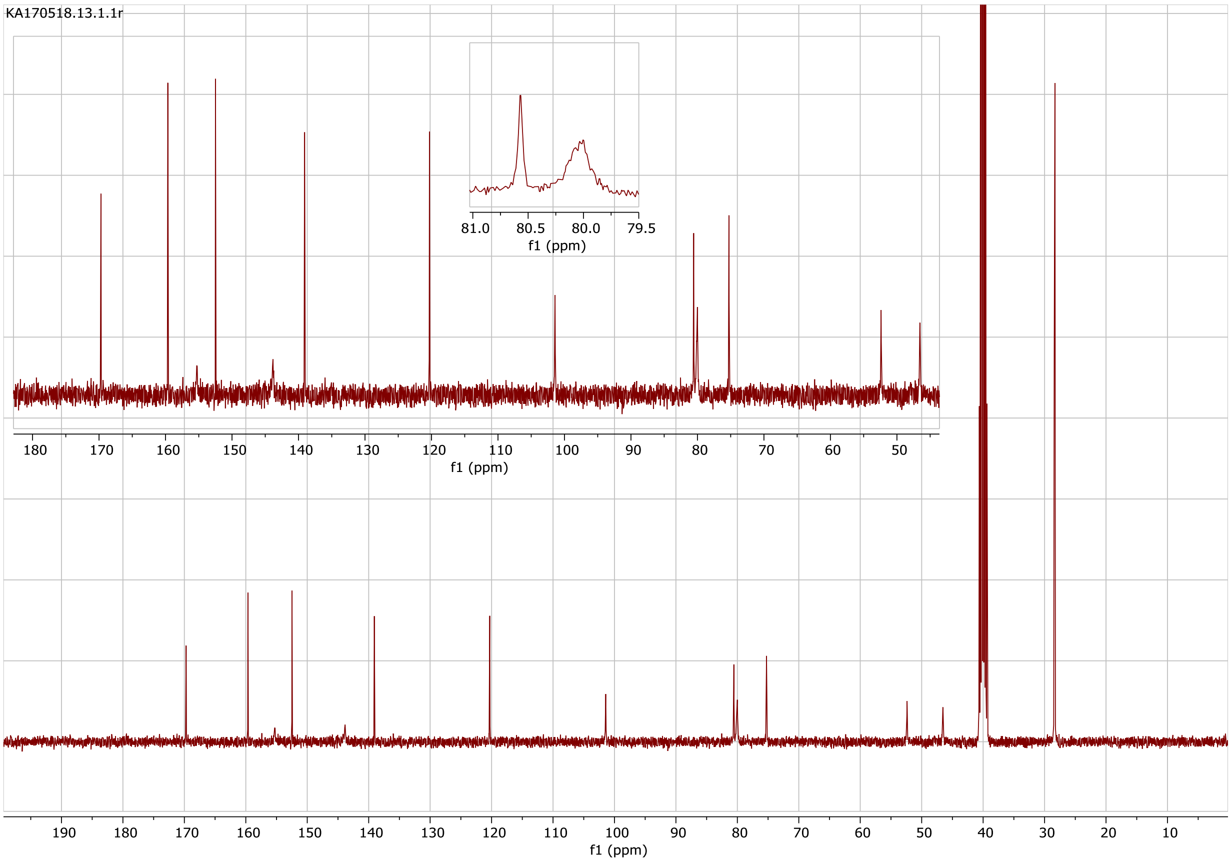

COSY & HSQC for dimer **17**

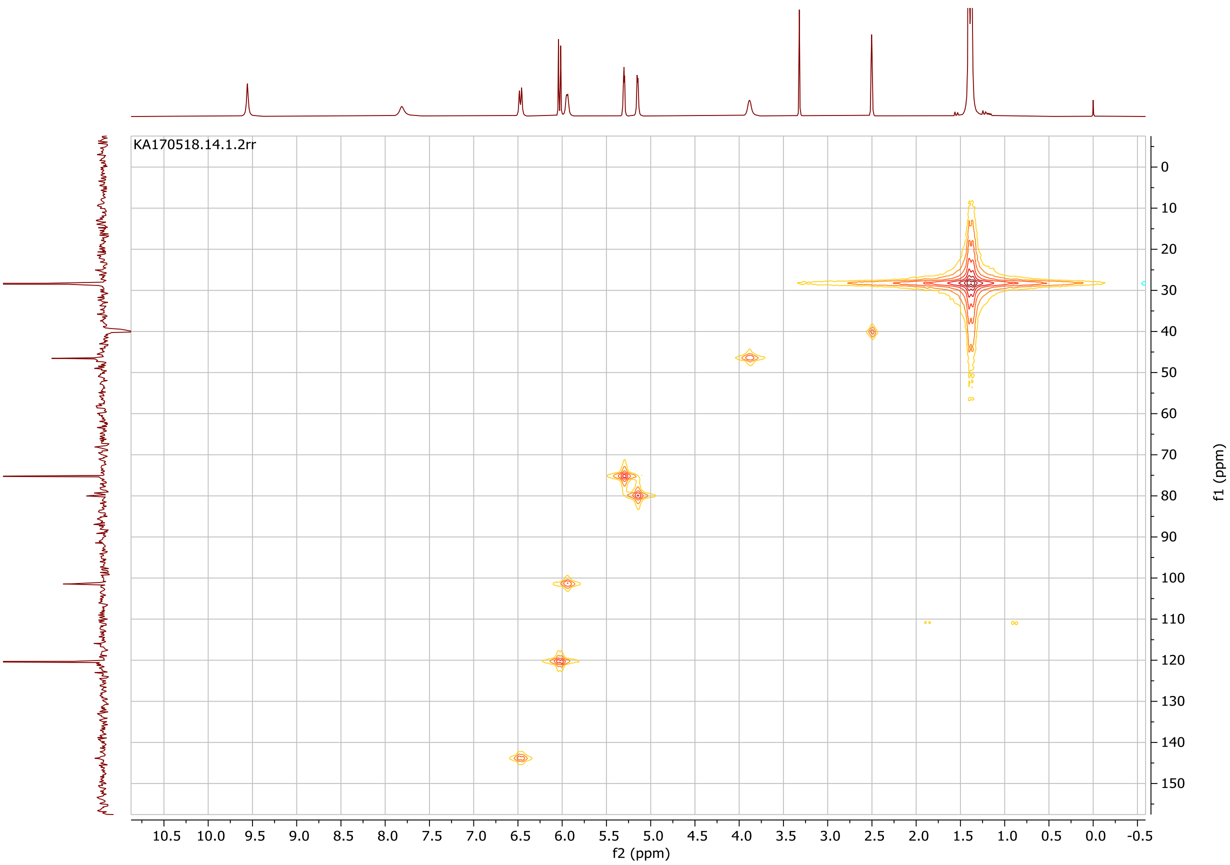

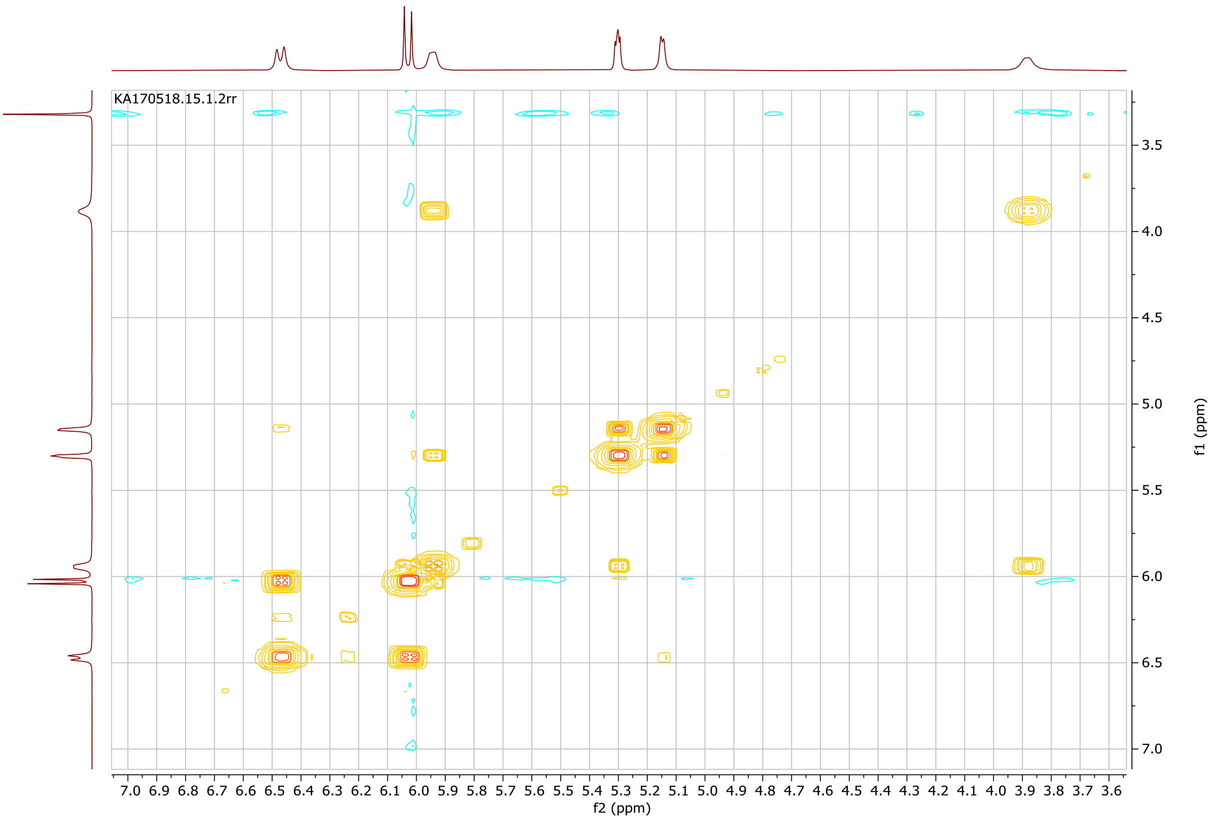

Assignment of ^{1}H and ^{13}C NMR signals for compound 17

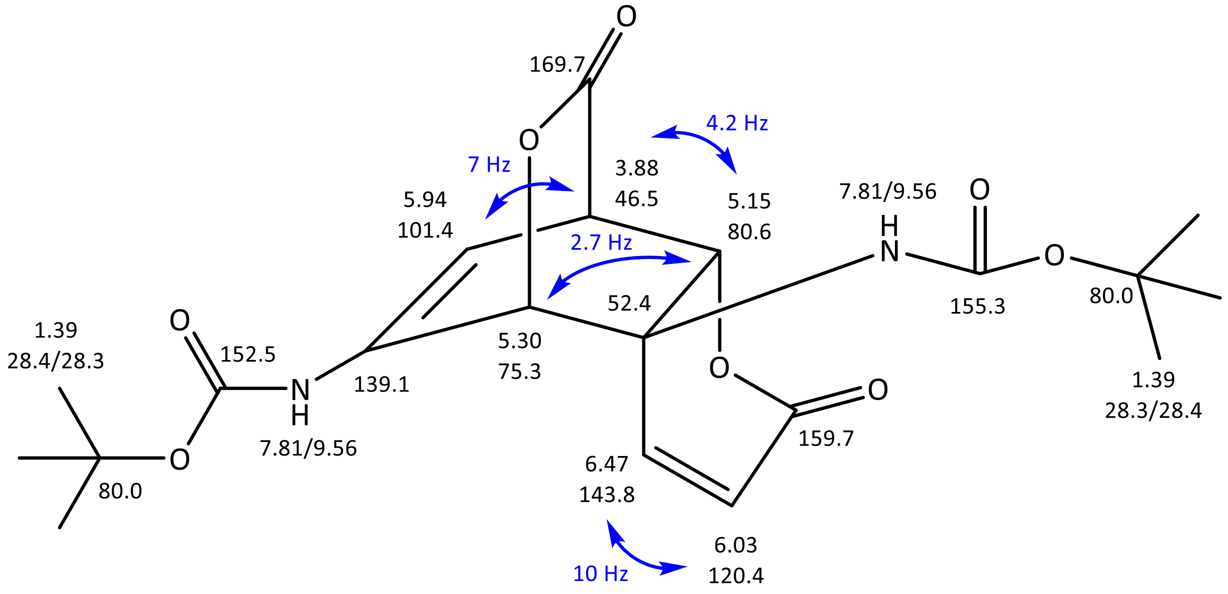

Observed		Predicted[1]		Observed		Predicted[1]	
δ_H	δ_C	δ_H	δ_C	δ_H	δ_C	δ_H	δ_C
1.37	28.4	1.42	28.4	7.81	-	8.48	-
1.40	28.3	1.42	28.4	9.56	-	9.21	-
3.88	46.5	3.67	35.5[2]	-	52.4	-	58.2
5.15	80.6	5.75	86.0	-	80.0	-	79.5
5.30	75.3	5.59	83.5[3]	-	139.1	-	130.0
5.94	101.4	5.02[4]	118.1[5]	-	152.5	-	148.3
6.03	120.4	6.25	119.1	-	155.3	-	155.6
6.47	143.8	6.38	144.9	-	159.7	-	162.1
				-	169.7	-	170.0

[1] We used Chemdraw v20.1 for prediction of chemical shifts

[2] In cycloadducts, this chemical shift is typically around 42 ppm which is closer to the observed value.

[3] In cycloadducts, this chemical shift is typically around 75 ppm which is closer to the observed value.

[4] In cycloadducts, this chemical shift is typically around 5.8 ppm which is closer to the observed value.

[5] In cycloadducts, this chemical shift is typically around 105 ppm which is closer to the observed value.
